# Collaborative Conservation Genetics of Cryptic Reptile Taxa From Northern Australia

**DOI:** 10.1002/ece3.73304

**Published:** 2026-04-09

**Authors:** Bridget L. Campbell, Yirralka Rangers, Yugul Mangi Rangers, Numburindi Rangers, Learning on Country – Laynhapuy Homelands School, Learning on Country – Shepherdson College, Rachael Y. Dudaniec, Craig Moritz, Jéssica Fenker, Emilie Ens

**Affiliations:** ^1^ School of Natural Sciences Macquarie University Sydney Australia; ^2^ Laynhapuy Homelands Aboriginal Corporation Yirrkala Northern Territory Australia; ^3^ South East Arnhem Land Indigenous Protected Area Northern Territory Australia; ^4^ Northern Land Council Northern Territory Australia; ^5^ Galiwin'ku Elcho Island Northern Territory Australia; ^6^ Research School of Biology Australian National University Canberra Australia; ^7^ Museums Victoria Research Institute Melbourne Australia

**Keywords:** *Carlia*, *Ctenotus*, *Diporiphora*, indigenous protected areas, indigenous rangers, reptile diversity

## Abstract

Estimates of global biodiversity are improving with the advent of novel molecular approaches, and this is especially true for reptiles. Across northern Australia, many endemic and cryptic reptile species have been discovered in recent years, and this region remains a priority area for taxonomic research. Collaborations between Indigenous land managers and researchers across remote, poorly surveyed regions can fill gaps in biodiversity knowledge, while simultaneously supporting local aspirations for value‐aligned employment and in contemporary conservation monitoring and science. Here, we apply collaborative sampling and genetic techniques with Indigenous rangers across poorly sampled East Arnhem Land to identify the species and lineages present across three reptile genera containing cryptic species complexes. Using a reduced representation DNA sequencing approach, we identified 13 taxa within *Carlia, Ctenotus*, and *Diporiphora*. We provide fine‐scale species distribution data, including 243 new occurrence records across 93 newly sampled locations, including some range extensions. This new knowledge will aid future collaborative surveys. Although sampling remains sparse, available evidence suggests a potential pattern of genetic divergence in the Blue Mud Bay region, aligning with boundaries of ecogeographic regions and Indigenous management areas. Further sampling is needed to provide greater evidence of population structure in East Arnhem Land. The ethical communication of the genetics results to the community was a key priority for Indigenous research partners. We applied a locally relevant Indigenous research approach to resolve mutual incomprehension and reveal parallel but distinct understandings of the genetic results. Yolŋu social organisation emerged as the key source of metaphor for contextualising the results across knowledge systems, with ‘gurrkurr’ (venous system, and by extension: root system and ‘blood line’) pivotal in communicating the phylogenetic tree results. Our collaborative approach could be applied across other Indigenous‐owned and managed lands of northern Australia and beyond to answer calls for collaborative conservation research practice.

## Graphical abstract

Here we apply collaborative sampling, genetic techniques and a local Indigenous research approach with Indigenous rangers across poorly sampled East Arnhem Land to identify the species and lineages across three cryptic reptile genera. Using a reduced representation DNA sequencing approach, we identified 13 species within Carlia, Ctenotus and Diporiphora genera. We provide fine scale species distribution data, including many new occurrence records and some range extensions to aid future collaborative surveys. Further sampling is required to support evidence for a landscape barrier driving local north–south population structure. A local Yolŋu research approach was used to investigate culturally relevant metaphors to communicate and discuss genetic results with Indigenous research partners.

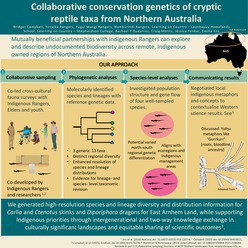

## 1 Introduction

Molecular approaches have redefined Western scientific understanding of biodiversity and revealed limitations of previous biodiversity estimates on the basis of morphological diagnostics. Reptiles are one such group of hyper‐diverse taxa for which global biodiversity is underestimated and in the process of being reevaluated (Pincheira‐Donoso et al. 2013). Australia boasts unique, diverse reptile fauna that is still being described and revised with increasing geographic sampling and use of genetic analyses (Flanagan et al. 2024). Some of this work is being conducted across data‐poor, remote regions of northern Australia that have been identified as priority areas for taxonomic research (Melville et al. 2021; Cremona 2025). Much of northern Australia is managed by Indigenous Ranger groups, setting the stage for mutually beneficial, collaborative research to better understand Australia's unique reptile diversity and biocultural diversity at large (Moritz et al. 2013; Ens et al. 2016). Such research, conducted in partnership with Indigenous Rangers, provides opportunities to meet local aspirations for intergenerational knowledge transmission, cross‐cultural or ‘two‐way’ (here: Indigenous and non‐Indigenous) knowledge sharing and capacity building on Indigenous‐owned lands (see Box 1b, Shaw et al. 2024).

Previous research suggests that northern Australia boasts impressive reptile diversity with high levels of species richness and endemism across numerous lizard taxa as well as non‐reptilian vertebrates (Powney et al. 2010). Genetic analyses have also revealed extensive morphologically cryptic reptile diversity, increasing known diversity and endemism of species and intraspecific lineages (Oliver et al. 2012, 2017; Moritz et al. 2016; Prates et al. 2023). High reptile endemism was revealed in the Kimberly region of northern Australia through collaborative research with the Bunuba and Gooniyandi Indigenous Rangers (Oliver et al. 2017). Unique reptile diversity was also found across the islands of north East Arnhem Land (Rosauer et al. 2016), a region managed by the Gumurr Marthakal Indigenous Rangers. In addition, Melville et al. (2019, 2021) identified the Kimberley and mesic Top End, spanning many Indigenous owned and managed regions, as high priority areas for targeted sampling to enhance taxonomic research on reptiles.

**FIGURE 1:**
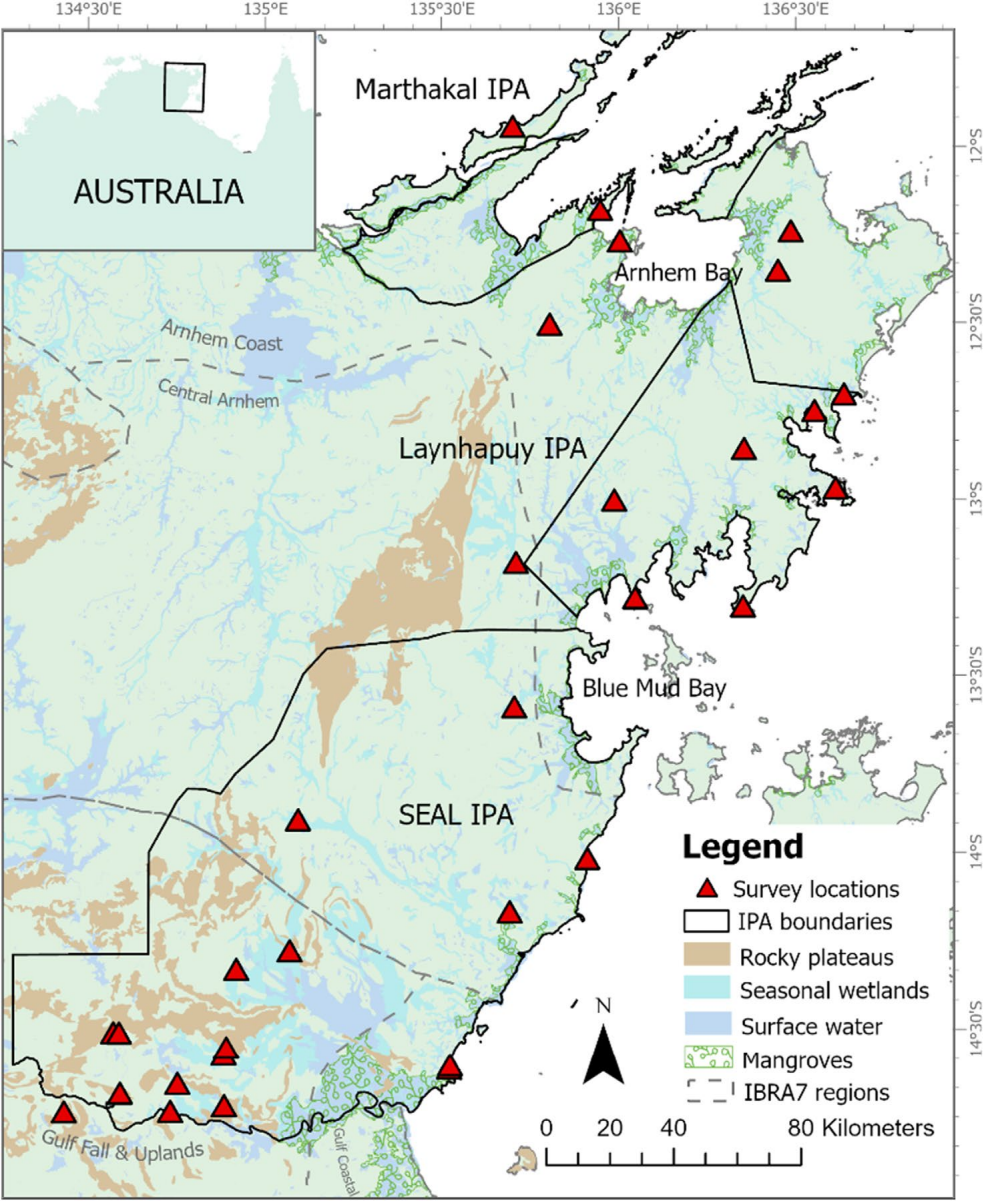
Map of East Arnhem Land indicating the survey sites (2014–2022) across the Marthakal IPA, Laynhapuy IPA and SEAL IPA, Interim Biogeographic Regionalisation for Australia (IBRA, v.7) regions, and major landscape features.

East Arnhem Land, in the mesic Top End of the Australian Monsoonal Tropics (AMT) (Figure 1), is a particularly under‐sampled region for terrestrial fauna diversity compared to other regions of the AMT. This area was declared Aboriginal land under the *Aboriginal Land Rights Act* (*Northern Territory*) (*1976*), which requires a permit for entry and is very remote, with limited infrastructure and service delivery, and low human population density. This has resulted in a paucity of scientific species occurrence records (e.g., Ens et al. 2016; Russell et al. 2023) and genetic research (Rosauer et al. 2016; Cremona et al. 2025). Currently, multiple Indigenous Ranger groups manage vast areas across East Arnhem Land as part of the Australian Indigenous Protected Area (IPA) program. The IPA program was established in 1997 and allows Indigenous groups to enter into voluntary agreements with the Australian government to protect their ancestral estates (National Indigenous Australians Agency 2023). IPAs and Ranger groups, which span > 50% of Australia's National Reserve system (92 million hectares) (Department of Climate Change, Energy, the Environment and Water (DCCEEW) 2024), have a strong history of collaborative research projects with external stakeholders that combine Indigenous and Western scientific knowledge and practices to monitor and manage local biodiversity (Kennett et al. 2004; Ens et al. 2016; Skroblin et al. 2022; Shaw et al. 2024). Here, the opportunity for mutual benefits from collaborative biodiversity monitoring and genetic sampling between researchers and Indigenous Ranger groups is large (Moritz et al. 2013; Ens et al. 2016; Russell et al. 2023).

Outcomes for Indigenous communities from collaborative conservation practice are enhanced when stakeholders pursue respectful partnerships that allow ample time to build trust, explore mutual benefits and local aspirations for cultural maintenance and cross‐cultural knowledge and skill transfer (Ens et al. 2016; Box 1b in Shaw et al. 2024). However, supporting Indigenous aspirations might require scientists to compromise on or adapt sampling design and methods and align with an inductive approach rather than a priori hypotheses‐driven research (Berkes 1999; Yunupingu and Muller 2009; Ens et al. 2016; Lilleyman et al. 2022), as we demonstrate here. It might also require engaging with social science and Indigenous research methods to ensure ethical and respectful partnerships (Lilleyman et al. 2022; Rayne et al. 2022; Shaw et al. 2024). This might leave collaborative research open to criticism, especially when the multiple benefits and layers of ‘biocultural’ protocols (Cooke et al. 2022) of their research practice is not considered. Furthermore, as Western scientific knowledge of diversity in this region was so sparse, this research naturally followed an exploratory approach, not in conflict with protocols of collaborative co‐design.

The study described here is part of a project between university researchers and the Yirralka Rangers, ‘Warrakan'puy djäma: Towards a Biocultural Approach to Conservation’, funded by the Australian Research Council, the Laynhapuy Homelands Aboriginal Corporation (who adminster the Yirralka Rangers) and The Nature Conservancy. This study builds on a decade long partnership between university researchers and the Yirralka Rangers of the Laynhapuy IPA and the Yugul Mangi Rangers of the South East Arnhem Land (SEAL) IPA that sought to foster cross‐cultural capacity building and biodiversity knowledge through on‐ground week long cross‐cultural fauna surveys in remote East Arnhem Land, Australia, following requests from local Elders and ranger groups and concern for native fauna (Ens et al. 2012, 2016; Ens 2012; Daniels et al. 2022; Campbell 2025). These surveys brought together Indigenous rangers, Elders, and youth participating in the Learning on Country (LoC) (Northern Land Council 2024) program, collaborating alongside university researchers, to survey sites of local interest chosen by Traditional Custodians. Tissue samples were collected from vertebrate terrestrial fauna trapped during surveys. In‐depth discussion of local aspirations, the co‐adapted cross‐cultural fauna survey methods and mutual benefits are presented in Ens et al. (2016) and Campbell (2025).

Although this article largely presents the results from a Western science‐focused part of this broader cross‐cultural project (focusing on the genetic analysis of the tissue samples collected from lizards during the surveys), involvement of Indigenous Rangers was not supplementary and extended beyond data collectors to decision‐makers and mutual benefactors in the larger project (see Campbell 2025; Campbell et al. 2024, 2025). Further, in line with best practice guidelines to decolonise Western science (Australian Institute of Aboriginal and Torres Strait Islander Studies (AIATSIS) 2020; Mc Cartney et al. 2022), this study had a negotiated exploratory research approach, where research questions were not defined a priori by Western scientists but co‐developed together during the research practice.

A key part of the East Arnhem Land cross‐cultural fauna surveys was to identify and record fauna using Yolŋu and Balanda (Western scientific) nomenclature, while negotiating meanings across the two systems as part of the survey process. Attempts by Western scientists to identify certain taxa, in this case small lizards, in terms of species, became a point of mutual incomprehension and thus interest within the cross‐cultural research team (Campbell 2025; Campbell et al. 2025). This was true for three types of Yolŋu‐recognised lizard: gunydjuḻu (small skinks); guḏutjurrk (large two‐lined skinks); and dhakarraŋbi (small dragons), within which university researchers attempted to identify species of the following genera: *Carlia* (within gunydjuḻu), *Ctenotus* (within guḏutjurrk) and *Diporiphora* (within dhakarraŋbi).

From a Western science perspective, *Carlia, Ctenotus*, and *Diporiphora* are species‐rich reptile genera with many small‐range and widespread species that have strong spatial genetic structuring across the AMT. Field identifications using morphological keys are often difficult for these genera because of subtle morphological differences. Recent genetic studies have revised traditional taxonomies or examined species delimitations using genetic analyses and discovered morphologically cryptic species complexes, highlighting the complexities of field identifications on the basis of morphology alone (*Carlia*: Potter et al. 2016; Bragg et al. 2018; Potter et al. 2019; Fenker et al. 2021), (*Ctenotus*: Rabosky et al. 2014; Singhal, Huang, et al. 2017; Prates et al. 2022; Prates et al. 2023), (*Diporiphora*: Melville et al. 2019; Fenker et al. 2021; Fenker et al. 2024). These studies primarily assessed genomic data that redefined species boundaries that were previously based on morphology or sequencing of just a few loci. Several of these studies used reduced‐representation sequencing to screen single nucleotide polymorphisms (SNPs) across the full geographic range of species, which provided valuable context for the current study. As Yolŋu and Western classification did not map onto each other for these groups (Campbell 2025; Campbell et al. 2025) and current Western classification is itself inadequate, Western scientists' taxonomic uncertainty in the field was not only a point of mutual incomprehension but remained unresolved (from a Western scientific perspective) without genetic analysis.

This study aimed to extend surveys in poorly sampled areas of East Arnhem Land and then apply genetic analyses to (1) improve knowledge of *Carlia, Ctenotus* and *Diporiphora* taxa and their distributions in East Arnhem Land; (2) detect any undiscovered species or genetic lineages that may be locally endemic to this region; (3) analyse the genetic variation and structure of four more thoroughly‐sampled species, *C. amax, C* *. munda* , *Ct. quirinus* and *D. bilineata* (as identified by aim 1) and investigate local patterns of population genetic structure and identify potential barriers to gene flow; and (4) feedback the results to Indigenous Rangers, Elders and youth in an accessible and meaningful way. Our research showcases how collaborative conservation practice between university researchers and Indigenous Ranger groups in under sampled, remote Indigenous owned regions can increase scientific understanding of biodiversity whilst simultaneously upholding Indigenous priorities to engage in cross‐cultural conservation practice, knowledge and skill sharing.

## 2 Methods

### 2.1 Surveys and Sample Collection

We conducted cross‐cultural broadscale surveillance fauna surveys across East Arnhem Land in collaboration with local Indigenous Ranger and Indigenous school Learning on Country (LoC) groups from the SEAL (2014–2019), Laynhapuy (2018–2022) and Marthakal IPAs (2022) (Figure 1) following modified Northern Territory Government terrestrial fauna surveys (see Ens et al. 2016). Ethics approval was granted by Macquarie University Animal Research Authority (Ref# 2016/017, 2017/040–7, 2017/040–8/9) and ANU Animal Ethics Committee (Ref# A2013_08, A2019_15). Northern Territory Parks and Wildlife Permits were granted (Ref# 70944, 54,202). Information on the study region is provided in the Text S1. For greater detail on the cross‐cultural design and benefits of the fauna surveys, see Campbell (2025) and Ens et al. (2016).

The fauna survey sampling design was co‐developed with Indigenous Ranger and LoC groups, and survey locations were selected in negotiation with Indigenous research partners who sought permissions from Traditional Custodians. Rangers selected locations of cultural significance, prioritising ancestral clan lands that had not been accessed or visited in years or decades. They aspired to keep connection and knowledge of these places strong and share this connection and knowledge with school students. Rangers also considered regions that were culturally significant for certain species of interest (see Campbell et al. 2024) or considered where these species were hunted for commonly in the past.

Across 31 locations (Figure 1), three sites in key habitat types (open woodlands, swamplands/billabongs, rocky escarpments, and coastal dunes) were sampled for 3–4 trapping nights per site. Two pitfall lines (1 × 10 m drift fence (1 × 20 L bucket), 1 × 20 m drift fence (3 × 20 L buckets)), funnel traps (*n* = 8), spotlighting (at least 20 min; adjacent to site), and opportunistic searches (approx. 10–20 min per site) were also used to trap small terrestrial fauna. Cross‐cultural field guides (co‐developed with the SEAL IPA Rangers (Ens et al. 2020) and the Yirralka Rangers (Yirralka Rangers, Macquarie University and Australian National University *unpub. data*)) were used alongside traditional field guides to identify organisms to the species or genus level (when species‐level identification was uncertain). Tissue samples (tail snips ~2–5 mm^3^ approx. volume) were preserved in 70% ethanol.

Species‐level identification of common *Carlia* and *Ctenotus* skinks and *Diporiphora* dragons was challenging in the field and unresolved for many individuals. As these genera had recently undergone taxonomic revision using genetic analyses (*Diporiphora*) (Melville et al. 2019; Fenker et al. 2024), or undergone phylogenetic analyses (*Ctenotus* and *Carlia*) (Rabosky et al. 2014; Potter et al. 2016; Prates et al. 2023) and were well represented in our sampling, we decided to focus our study on investigating the genetic diversity of these three genera.

### 2.2 Study Taxa

#### 2.2.1 *Carlia ~* Four‐Fingered (Rainbow) Skinks *~* Gunydjuḻu

*Carlia* is a species‐rich genus of 27 currently recognised small, diurnal four‐fingered rainbow skink species, with a broad distribution across the Australian continent and Papua New Guinea (Jolly et al. 2023). Seven species occur in the Northern Territory (NT) of Australia and sympatric species with similar morphology can be hard to identify (Jolly et al. 2023). Six of the seven NT species of *Carlia* have ranges that could extend into East Arnhem Land (Jolly et al. 2023), including: *C. amax; C* *. gracilis* *; C* *. munda* *; C. sexdentata; C. rufiliatus;* and *C. triacantha*, and several are known to have multiple, deeply divergent genetic lineages across the Top End, including taxa endemic to the islands off the north cost of East Arnhem Land (Potter et al. 2016, 2018). These lineages, which we regard as candidate species pending formal taxonomic revision, have been defined by their geographic distribution and include: ETE (Eastern Top End); WTE (Western Top End); GULF (Gulf of Carpentaria); KIM (Kimberley region); and ECI (English Company Islands) (Potter et al. 2016, 2018; Fenker et al. 2021).

#### 2.2.2 *Ctenotus ~* Comb‐Eared Skinks ~ Guḏutjurrk

*Ctenotus* (‘comb‐eared skinks’) is the most speciose taxon of scincid lizards in Australia, and most diverse reptile genus in the NT with 52 species recorded, including 25 across the Top End (Rabosky et al. 2014; Jolly et al. 2023). *Ctenotus* species have long been considered difficult to identify in the field because of ambiguous morphological characteristics used in species delimitation (e.g., dorsal colouration patterns, scalation and body proportions) and consequently they have uncertain range boundaries (Bush et al. 2007; Jolly et al. 2023). Under current taxon delimitations, three species groups have distributions across East Arnhem Land: the *Ct. arnhemensis, Ct. essingtonii* and *Ct. inornatus* groups. From the *Ct. arnhemensis* group *Ct. astictus* has been described from samples across west and south east Arnhem, and the Wessel Islands (Horner 1995). From the *Ct. essingtonii* group *Ct. vertebralis* occurs on the Arnhem plateau in central Arnhem Land (Rankin and Gillam 1979) and *Ct. quirinus* is reported to occur across northern and central Arnhem Land (Horner 2007) and was genetically identified on Groote Eylandt as one lineage within the highly structured *Ct. essingtonii* species complex (Rosauer et al. 2016). Lineages of *Ct. essingtonii* itself (1,2 and 3) as far as is known have non‐overlapping geographic locations in the north and west of the Top End (CM unpublished data; Prates et al. 2022). The *Ct. inornatus* species group is considered a ‘taxonomic disaster zone’ with multiple species complexes and ‘extreme intraspecific morphological variation’ (Rabosky et al. 2014; Prates et al. 2023). The Australian Society of Herpetologists (ASH) recommended broad spatial and genomic sampling to better understand the diversity of this group (Australian Society of Herpetologists 2022). The *Ct. inornatus* group contains three species complexes (*Ct. inornatus*, *Ct. superciliaris* and *Ct. robustus*), each of which contains lineages, regarded as candidate species, that could occur in East Arnhem Land (inornatus‐N (north), robustus‐NW (north west), spaldingi‐NE (north east), superciliaris‐E (east); Prates et al. 2023).

#### 2.2.3 *Diporiphora ~* Two‐Lined Dragons ~ Dhakarraŋbi

*Diporiphora* is a species‐rich genus of 21 small, morphologically conservative, diurnal agamid lizards (Melville et al. 2019). Recent taxonomic revisions revealed two *Diporiphora* ‘species groups’ ( *D. bilineata* and *D. bennettii* ), which have broad distributions across northern Australia and are similar in ecology and morphology (Melville et al. 2019). *Diporiphora* have diversified extensively and have strong spatial structuring of intraspecific lineages across northern Australia, some of which could represent cryptic species (Fenker et al. 2024). Two species from the *D. bilineata* group ( *D. bilineata* , *D. magna* ), and one from the *D. bennettii* group ( *D. sobria* ) occur across East Arnhem Land (Melville et al. 2019). Lineages for these species have been geographically defined as follows: *D. bilineata* (WTE: Western Top End, and ETE: Eastern Top End), *D. magna* and *D. sobria* (WTE, ETE and KIM: Kimberley region) (Fenker et al. 2021, 2024).

### 2.3 DNA Extractions

DNA was extracted from *Carlia* (*n* = 184), *Ctenotus* (*n* = 91) and *Diporiphora* samples (*n* = 64) using either a DNeasy Blood and Tissue Extraction Kit (QIAGEN) or a NucleoSpin 96 Tissue Core Extraction Kit (Marcherey Nagel). A 3 mm^3^ piece of tissue was homogenised using a sterile mini‐pestle and extracted using the manufacturer's protocol. An Invitrogen Qubit 2.0 Fluorometer (ThermoFisher) was used to quantify extracted DNA from QIAGEN kits with a Qubit Broad Spectrum Assay Kit. DNA extractions from the Macherey Nagel kit were quantified using a Qubit Flex (Broad Spectrum). *Carlia, Ctenotus*, and *Diporiphora* samples from this study were co‐analysed with genus‐level reference samples from previous studies (genus‐level SNP datasets)(Table S8).

### 2.4 Single Nucleotide Polymorphism Genotyping and Filtering

SNPs were identified and genotyped using the proprietary DArTseq method of genome reduction via restriction enzymes (see Jaccoud et al. 2001). The restriction enzyme N1aIII was selected following initial screening for *Carlia* (MyTaq‐EBPCR1 + NlaIII), *Ctenotus* (MyTaq‐EBPCR1 + NlaIIIv4), and *Diporiphora* (MyTaq‐EBPCR1 + NlaIIIv4). DNA fragments were amplified using PCR and single‐end sequenced using Illumina Hiseq2500. Insert length was 69 bp. For further details of SNP genotyping see Georges et al. (2018) and Text S2. The SNPs were called from short‐read sequence data using proprietary DArT analytical pipelines in combination with *Carlia* and *Diporiphora* genus‐level reference samples (Fenker et al. 2021; C.M. unpublished data) (Table S10). Given that prior studies of SNP variation in *Ctenotus* used a different method (ddRAD), we could not combine new and old data directly. A selection of *Ctenotus* samples previously allocated to lineages in Prates et al. (2022, 2023) were here resequenced using DArT technology to allow us to match our samples to previously identified species and lineages (see Table S9 for details).

### 2.5 Data Filtering and Species Identification

The *Carlia, Ctenotus* and *Diporiphora* genus‐level SNP datasets were filtered using a data‐driven approach to threshold selection on the basis of visual inspection of plotted distribution of SNP data for each metric using the *report* function of dartR (v2; Mijangos et al. 2022). Filtering metrics (and initial tested thresholds) were included: monomorphic loci; reproducibility (≥ 90%); read‐depth (8–10 ≥ ≤ 20–60); locus call‐rate (≥ 0.60); minor allele frequency (≥ 0.02) and secondaries (SNPs that share a sequence tag and are likely linked) using the dartR package (v2) (Mijangos et al. 2022) in RStudio Version 2023.06.1 + 524 (RStudio Core Team 2023). Please see the Tables S1–S7 for filtering thresholds selected for each genus and species‐level dataset. Filtering results are presented in Figure S1–S3.

To allocate study samples to species and lineages, we first visually examined genetic clustering from hierarchical principal component analyses (PCA) (DartR package v2). We then conducted phylogenetic analyses following the multispecies coalescent framework using SVDquartets v.1 (Chifman and Kubatko 2014) run via the command line version of PAUP v. 4.0a (Swofford 2016). All possible quartets were sampled, and node support was estimated using 100 bootstrap replicates. Figtree (v1.4.4; Rambaut 2018) was used to edit trees and produce figures. Taxon identifications were inferred from visual inspection of phylogenetic trees and PCAs and checked against known distributions of *Carlia, Ctenotus* and *Diporiphora* species or lineages. For species of *Ctenotus* not previously included in DArT SNP screens, we leveraged multilocus sequence data for this genus generated via target capture (SqCL loci; Singhal, Grundler, et al. 2017 and reported in Title et al. 2024). We included representatives of each genetic group revealed here in a parallel study by the Australian Amphibian and Reptile Genomics initiative of Bioplatforms Australia (J. Torkkola, S. Tiatragul, D. Rabosky, *pers. comm*.) and analysed as described in Brennan et al. (2024). Following their preliminary phylogenetic analyses, we were able to confirm most lineage IDs. Notably, prior sequence data backed by voucher specimens were not available for *Ct. asticus* or *Ct. vertebralis*.

### 2.6 Genetic Diversity and Structure of *C. Amax and C. munda, Ct. Quirinus and D. bilineata*

For population genetic analyses, we created four species‐level SNP datasets for species (or distinct lineages thereof) that had the largest sample sizes and broadest geographic distributions: *C. amax* (Eastern Top End (ETE) lineage; *n* = 50); *C. munda* (*n* = 62); *Ct. quirinus* (*n* = 24); and *D. bilineata* (Eastern Top End (ETE) lineage; *n* = 47). We then re‐filtered the SNP data for each of these species following a data‐driven approach as reported above using dartR (Mijangos et al. 2022) for: monomorphic loci; reproducibility (≥ 90%–5%); read‐depth (8–10 > 20–60); loci call‐rate (≥ 0.75–0.80); individual call‐rate (≥ 0.20–0.50); minor allele frequency (≥ 0.02); and secondaries. Exact filtering thresholds are provided in Tables S2–S3, S5, S7. SNP datasets were pruned for loci under strong linkage‐disequilibrium (LD; *R* ^2^ = 50%) and for loci that significantly deviated from the Hardy–Weinberg Equilibrium (HWE) at a significance level of 0.001 using Plink v.1.9 (Purcell et al. 2007) (Tables S2–S3, S5, S7). Filtering results are presented in (Figure S4–S7).

To visualise genetic structure and divergence across samples, three complementary genetic clustering approaches were used, PCA, sNMF and PopCluster. PCA analysis was performed using the dartR package. Population structure and individual admixture proportions were inferred using sNMF (Frichot and François 2015) and PopCluster v1.2.0.0 (Wang 2022). sNMF estimates patterns of admixture and allele sharing and identifies the most likely number of genetic clusters (K) using ‘sparse non‐negative matrix factorisation’ which is robust against deviations from HWE (Frichot and François 2015). To infer the best‐fitting number of populations (genotypic clusters) we compared *K* = 1–10 with 100 repetitions. The *K* with the lowest cross‐entropy score was used to select the most likely number of populations. The proportion of ancestry to each K cluster for individuals was plotted using lattice v0.22–5 (Sakar 2008) and mapped.

We performed PopCluster analysis using the admixture model and, as with sNMF, *K* values of 1–10 were compared with 100 repetitions. The second order rate of change of the estimate log‐likelihood (DLK2) was used to determine the best number of *K* (Wang 2022). Previous studies on *Diporiphora* and *Ctenotus* have used sNMF (Fenker et al. 2021; Prates et al. 2023); however, PopCluster is considered more accurate than other widely used genetic structure software (e.g., STRUCTURE and sNMF) when sampling is unbalanced, and sample size is small per population (Wang 2022), as is the case here.

The relationship between genetic and Euclidean geographic distance (isolation by distance; IBD) was examined using a Mantel test with 10,000 permutations using adegenet v2.1.10 (Jombart & Ahmed 2011). Inbreeding coefficient (*F*_*IS*_) was calculated for identified populations using the hierfstat package v0.5.11 (Goudet 2005). Pairwise genetic differentiation (*F*_*ST*_ *)* (Weir and Cockerham 1984) was calculated between each genetic cluster using 1000 iterations to generate bootstrapped confidence intervals with the dartR package (Mijangos et al. 2022).

### 2.7 Effective Migration Surfaces (EEMS)

To estimate comparable spatial patterns of connectivity across the study area, we ran Estimated Effective Migration Surfaces (EEMS) (Petkova et al. 2016) for *C*. *amax, C. munda, Ct. quirinus* and *D. bilineata* respectively. EEMS uses a Markov Chain Monte Carlo (MCMC) approach to generate contour plots of estimated effective migration rates (*m*) and diversity (*q*) that are mean‐centred on a log10 scale by identifying regions that deviate from the Isolation by Distance (IBD) null assumption. EEMS generates *m* and *q* by using a triangular grid comprised of ‘demes’ to which georeferenced genetic data are assigned. Bayesian inference is used to estimate *m* for every deme edge and *q* for each deme. Here, *m*, the migration rate, is estimated by analysing the genetic dissimilarities (squared genetic difference across all markers) between demes, and *q*, or effective genetic diversity, is estimated by analysing the genetic dissimilarities between two individuals from the same deme. EEMS assumes an underlying stepping stone model (Kimura and Weiss 1964) of migration and so is best applied to samples from the same historical genetic lineage.

*Bed2diffs* was used to generate a matrix of pairwise genetic dissimilarities between individuals of each dataset required for EEMS. *RunEEMS_snps* was run independently three times to ensure random sampling, with 400 demes, 4 million iterations, 3 million burn‐in iterations, and a thinning interval of 9999. The three independent runs were combined and rEEMSplots v0.0.1 (Petkova et al. 2016) was used to plot *m* and *q*. Multiple analyses were run to test different combinations of deme size (250, 300, 350, 400), iterations (2, 3 and 4 million), and burn‐in parameters (1, 2 and 3 million), as is recommended in Petkova et al. (2016). Visual inspection of the posterior trace plot was used to check convergence of the MCMC (Figure S8a–d).

### 2.8 Cross‐Cultural Knowledge Transfer: Communicating the Results

During this project, university researchers aspired to meet best practice guidelines for working with Indigenous research partners (AIATSIS 2020; Mc Cartney et al. 2023). This included working towards ethical knowledge sharing, involving communication of the results of cross‐cultural fauna surveys and genetics analyses in accessible and contextualised ways through presentations, discussions, and the generation of bilingual community reports and short videos (see Campbell 2025). To feedback the results of the genetics analyses, author BC worked closely with Yirralka Rangers and Ŋaḻapalmi (knowledge holders) and adopted a Yolŋu research approach of ‘dhawurrpunaramirri’ (Yolŋu two‐way negotiation and discussion (Wunuŋmurra, 1989)) to identify conceptual equivalences between Western science genetics concepts and Yolŋu concepts. Author BC engaged in dhawurrpunaramirri with three Yirralka Rangers with whom they had a long and trusting relationship (~5 years). This process involved three key stages: (1) initial non‐structured and informal discussions (presenting of results, discussion of key concepts, planning); (2) more structured organised sessions with Ŋaḻapaḻmi and other Yirralka Rangers involved in the project to verify and seek permission to use Yolŋu 'equivalent' concepts; (3) continued negotiation and discussion of concepts whilst working on a Yolŋu Matha transcript for a video summarising key methods and results. These sessions were held across 4 weeks and lasted ~20–90 min depending on the availability of rangers and Ŋaḻapaḻmi, and to work around other Yirralka Ranger work and obligations and researcher fieldwork constraints. Free and prior informed consent was sought (in English and in Yolŋu Matha) before recording sessions with rangers and Ŋaḻapaḻmi, who selected how they would like their knowledge acknowledged and shared in accordance with Macquarie University Human Research Ethics Committee approval (Ref: 5201800178). Yolŋu knowledge featured in this article is the intellectual property of Yolŋu Yirralka Rangers and Ŋaḻapaḻmi. More detailed methods on the application of dhawurrpunaramirri to contextualise the genetics results are documented in Campbell et al. (2025).

## 3 Results

### 3.1 Species Identification and New Records

**FIGURE 2:**
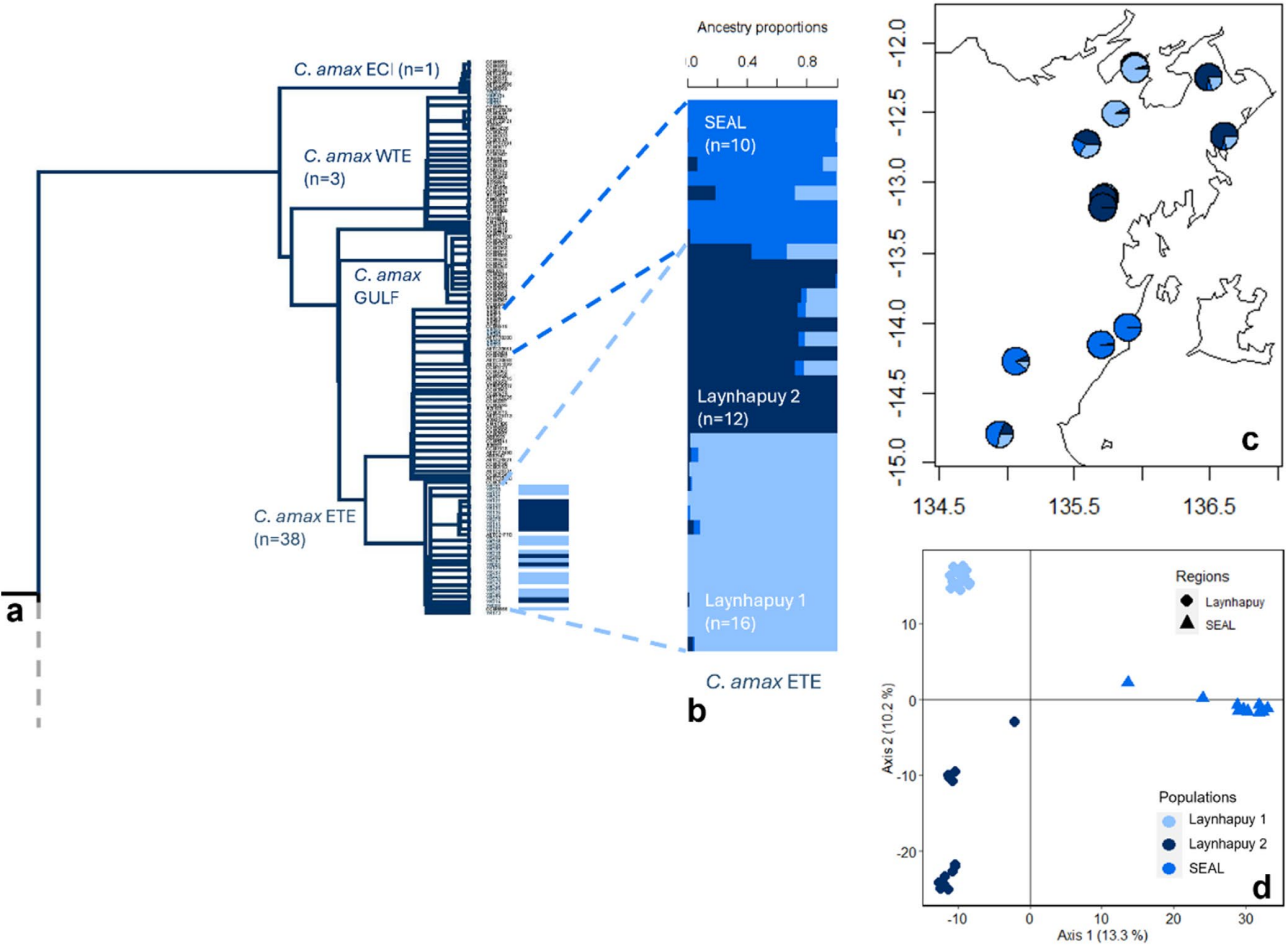
Results of genetic structure analysis for *C. amax* (ETE). (a) the *C. amax* region of the *Carlia* phylogenetic tree, including geographically described lineages (ETE = Eastern Top End, ECI = English Company Islands, WTE = Western Top End, and GULF = Gulf of Carpentaria). Coloured dotted lines and bars indicate *C. amax* ETE samples included in the following analyses and their assignment to populations from: (b) sNMF population structure; (c) mapped individuals with sNMF results, and (d) principal coordinate analysis with colours of individuals reflecting sNMF population allocation Laynhapuy 1 = light blue, Laynhapuy 2 = navy, SEAL = dark blue.

**FIGURE 3:**
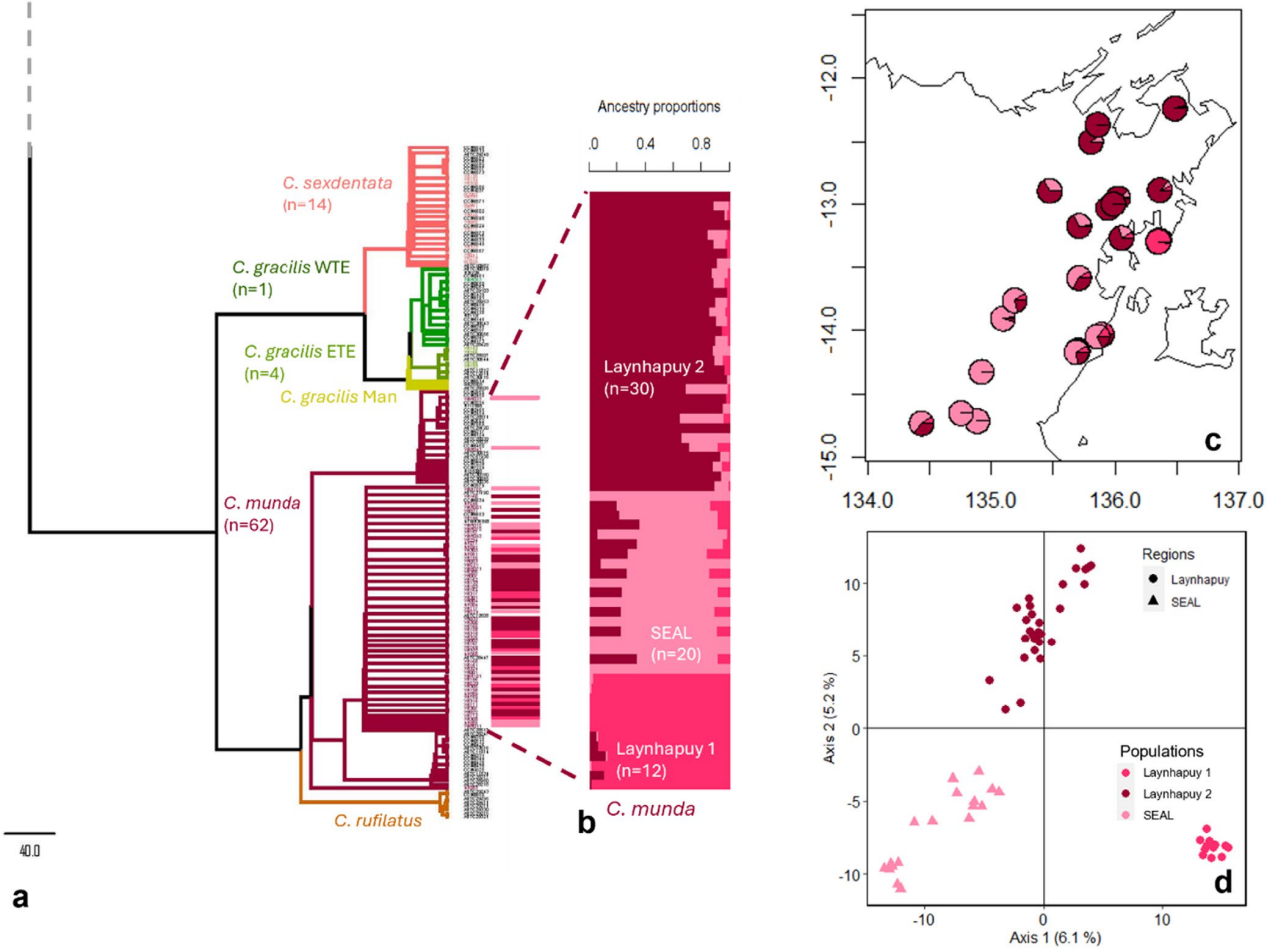
Results of genetic structure analysis for *C. munda* . (a) the *C. munda* region of the *Carlia* phylogenetic tree, coloured dotted lines and coloured bars indicate *C. munda* samples included in the following analyses: (b) sNMF population structure; (c) mapped individuals with sNMF results, and (d) principal coordinate analysis with colours of individuals reflecting sNMF population allocation (Laynhapuy 1 = hot pink, Laynhapuy 2 = dark red, SEAL = light pink).

**FIGURE 4:**
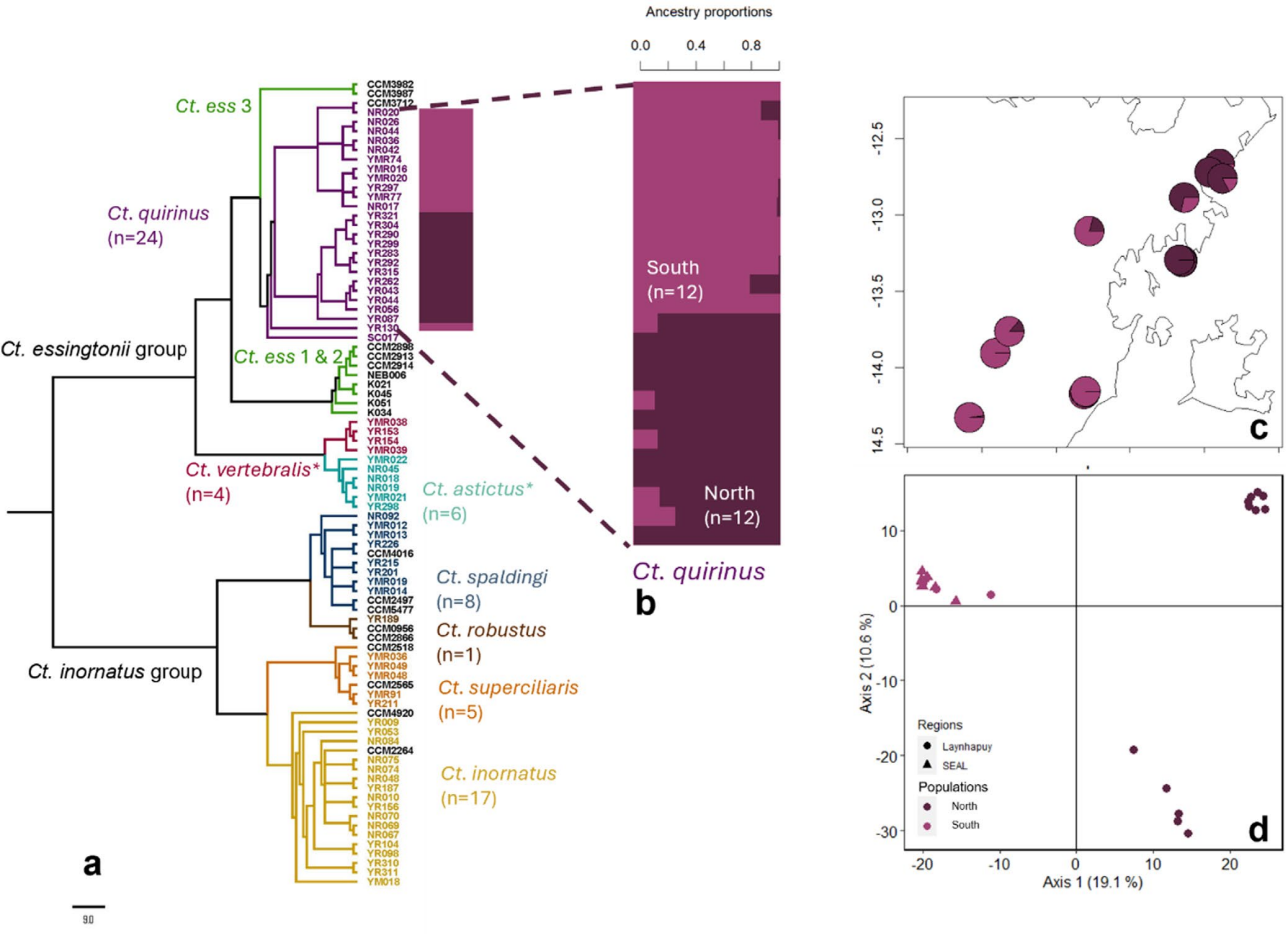
Results of genetic structure analysis for *Ct. quirinus*: (a) *Ctenotus* phylogenetic tree, dotted lines and coloured bars indicate *Ct. quirinus* samples included in the following analyses, *Ct. ess* refers to different lineages of *Ct. essingtonii* (1, 2 and 3) as identified in Prates et al. 2022: (b) sNMF population structure; (c) mapped individuals with sNMF results and (d) principal coordinate analysis with colours matching the sNMF population allocation (North = dark purple, South = light purple).* next to species names indicates putative identification.

**FIGURE 5:**
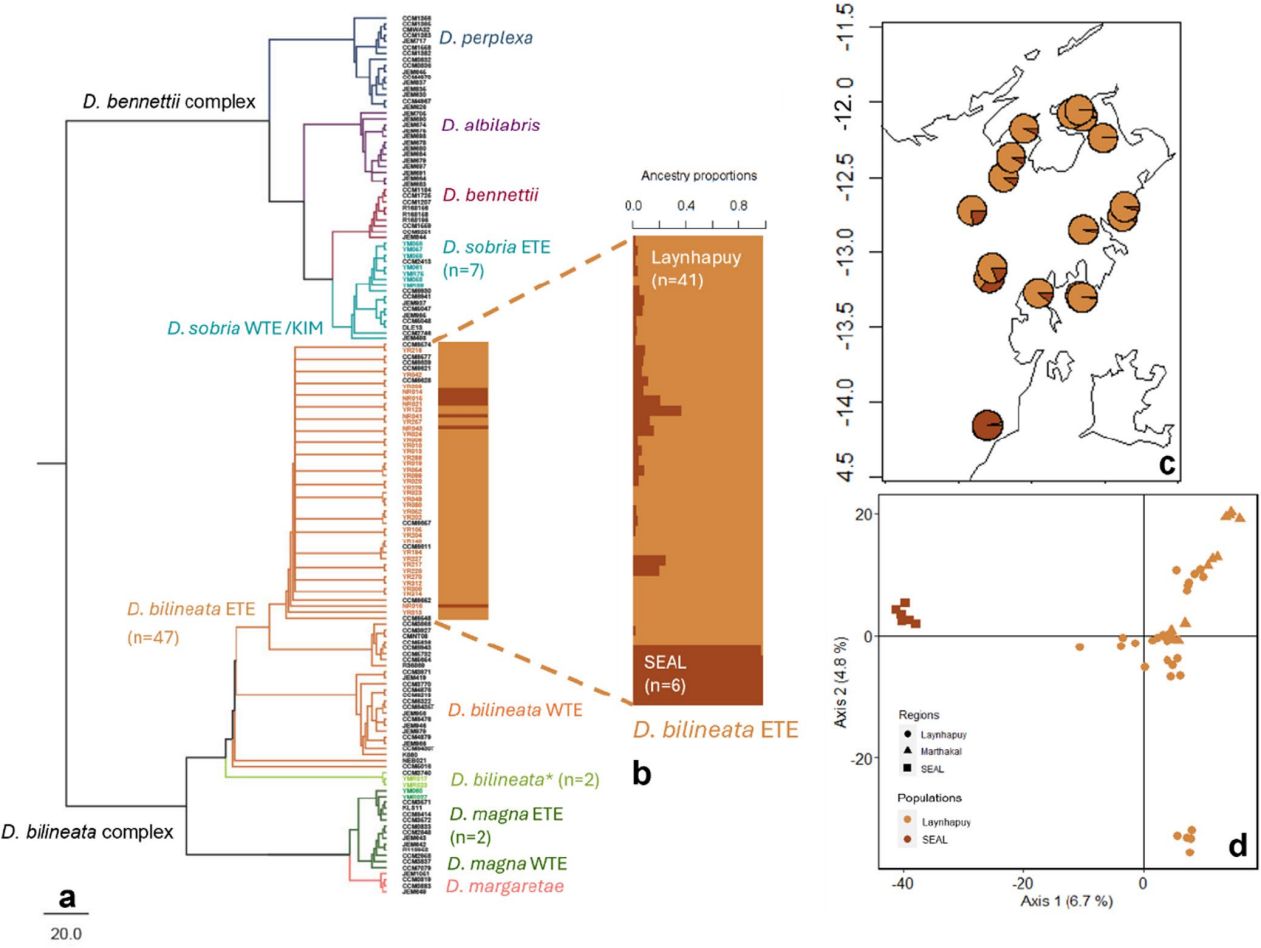
Results of genetic structure analysis for *D. bilineata* (a) phylogenetic tree of *Diporiphora* species, including geographically described lineages (ETE = Eastern Top End, WTE = Western Top End, and Kim = Kimberley region), dotted lines and coloured bars indicate *D. bilineata* samples included in the following analyses: (b) sNMF population structure; (c) mapped individuals with sNMF results and (d) principal coordinate analysis with colours matching the sNMF population allocations (Laynhapuy = light orange, SEAL = dark orange).* following *D. bilineata* (light green) represents individuals of an undescribed lineage.

After filtering SNPs, we retained 5399 independent SNPs in the *Carlia* genus‐level dataset, 12,448 in the *Ctenotus* genus‐level dataset and 1758 SNPs in the *Diporiphora* genus‐level dataset (Table 1). Species and lineage identities were inferred from SNP data for new samples by matching to prior evidence using a combination of hierarchical PCAs and phylogenetic analyses (Figures 2, 3, 4, 5; Figure S9–S13). Overall, 40% of field‐based species identifications were initially ambiguous or were incorrect relative to genetics‐based diagnostics: ~17% for *Carlia*, ~90% for *Ctenotus* and ~27% for *Diporiphora*. Improved identifications were obtained for all genera (Table 2). The field surveys, supported by genetic data, also resulted in 243 new species observations representing 93 new occurrence records for the IPAs, including extensions to range limits for species or lineages. Details are provided for each taxon in Table 2. For mapped occurrence records, see Figure S14–S16.

**TABLE 1:** Rows refer to results pre‐ and post‐filtering metrics of genus‐ and species‐level data.

Genus/species	*N* samples before filtering	*N* samples after filtering	Loc before filtering	Loc after filtering	Data missingness (%)	Av read depth
*Carlia* genus	338	336	59,302	5399	22.47	23.05
*C. amax*	38	38	157,563	5429	8.21	15.5
*C. munda*	63	62	203,759	5361	3.29	20.61
*Ctenotus* genus	88	86	223,037	12,448	25.28	16.25
*Ct. quirinus*	24	24	223,037	3140	1.68	15.49
*Diporiphora* genus	155	148	80,865	1758	25.73	10.83
*D. bilineata*	47	47	141,406	8504	2.16	11.15

**TABLE 2:** Field and revised molecular species and lineage identifications (ID) demonstrating the number of new observations of species and occurrence records for *Diporiphora, Carlia*, and *Ctenotus* species from East Arnhem Land.

	Species	Field ID (*n*)	Molecular ID: Total new observations (*n*)	Lineage IDs	Number of new occurrence records in each Indigenous Protected Area (IPA)
*Carlia*	*Carlia* sp.	11*	0		
	*C. amax*	46	47	ETE, WTE, ECI	Marthakal (1), SEAL (4), Laynhapuy (6)
	*C. gracilis*	—	5	ETE, WTE	Laynhapuy (3), SEAL (1)
	*C. munda*	67	63		Laynhapuy (8), SEAL (10)
	*C. rufilatus*	1	0		
	*C. sexdentata*	4	14	TE	Marthakal (2), Laynhapuy (6)
	TOTAL	118	129		41
*Ctenotus*	*Ctenotus* sp.	33*	4*		
	*C.astictus*	—	6		Laynhapuy (1), SEAL (2)
	*C. essingtonii*	6	0		
	*Ct. quirinus*	1	25		Marthakal (1), Laynhapuy (6), SEAL (4)
	*Ct. inornatus*	10	17	N	Laynhapuy (6), SEAL (4)
	*Ct. robustus*	19	1	TE	Laynhapuy (1)
	*Ct. spaldingi*	1	9	NE	Marthakal (1), Laynhapuy (2), SEAL (1)
	*Ct. superciliaris*	—	5	E	Marthakal/Laynhapuy (1), SEAL (2)
	*Ct. vertebralis*		4		Laynhapuy (1), SEAL (1)
	TOTAL	37	66		34
*Diporiphora*	*Diporiphora* sp.	5*	6*		
	*D. bilineata*	48	40	ETE	Marthakal (1), Laynhapuy (10), SEAL (2)
	*D. lalliae*	3	0		Laynhapuy
	*D. magna*	—	2	ETE	SEAL (2)
	*D. sobria*	—	6	ETE	SEAL (3)
	TOTAL	49	48		18
Total records with molecular species level ID			243	Total new occurrence records	93

* missing data and/or numbers not included in counts. Lineage identifiers (ETE = Eastern Top End, WTE = Western Top End, ECI = English Company Islands, NE = North East, E = East, NW = North West, TE = Top End).

### 3.2 Carlia

We found evidence of four species encompassing seven lineages of *Carlia* that were distributed across East Arnhem Land: *C. amax* (Eastern Top End (ETE); Western Top End (WTE); English Company Islands (ECI)) (Figure 2a)*; C. gracilis* (ETE and WTE); *C. munda*, and *C. sexdentata* (Figure 3a; Table 2). The majority of *C. amax* (90%) and *C. gracilis* (80%) samples grouped within the geographically relevant ETE lineages from Potter et al. (2016) and Fenker et al. (2021). Individuals that grouped with the WTE lineages (*C. amax* and *C. gracilis* ) were found only in the south of the SEAL IPA. We found the first evidence that the *C. amax* ECI island lineage also occurs on the adjacent mainland, in the north east of the study region (Laynhapuy IPA and Marrthakal IPA shared management region). The majority of *C. amax* ETE individuals (~74%) from this study formed a genetically distinct clade relative to previously analysed samples with strong support for node separation (94 Bootstrap value) (Figure 2a). Collectively, these records extend known geographic ranges for *C. gracilis* (ETE and WTE lineages) and for the *C. amax* ECI lineage.

### 3.3 Ctenotus

We found evidence for seven species of *Ctenotus* distributed across East Arnhem Land: *Ct. inornatus, Ct. superciliaris, Ct. spaldingi, Ct. robustus, Ct. astictus, Ct. vertebralis*, and *Ct. quirinus*. For the *Ct. inornatus* species group (*Ct. inornatus, Ct. robustus, Ct. spaldingi, Ct. superciliaris*), grouping was concordant with previous studies where most individuals fit into previously defined species and/or lineages presumed to occur across East Arnhem Land (Prates et al. 2022, 2023) (Table 2, Figure 4a). As inferred from previous mtDNA studies (Rosauer et al. 2016), individuals of *Ct. quirinus* form a clade within the *Ct. essingtonii* species complex indicating that this complex requires further taxonomic analysis. We provide the first molecularly confirmed record of *Ct. quirinus* from Elcho Island which appears genetically distinct from mainland *Ct. quirinus* samples. This divergent sample was not included in population genetic analysis of *Ct. quirinus*. The sister group to the *Ct. essingtonii* complex is provisionally identified as *Ct. astictus* (*n* = 6) and *Ct. vertebralis* (*n* = 4). The former has not been included in any previous genetic study of *Ctenotus* and museum specimens with accompanying genetic data are needed to verify the records for this species. The identification of samples as *Ct. vertebralis* is based on inclusion of some samples from this study in preliminary phylogenetic analyses of all *Ctenotus* species by collaborators (J. Torkkola, S. Tiatragul, D. Rabosky, *pers. comm*.).

### 3.4 Diporiphora

We found evidence for three species across two species groups of *Diporiphora* distributed across East Arnhem Land: *D. bilineata* group ( *D. bilineata* and *D. magna* ) and *D. bennetti* group ( *D. sobria* ) (Table 1, Figure 5a). *D. magna* and *D. sobria* individuals aligned with the ETE (Eastern Top End) lineages described by Fenker et al. (2020, 2023) (Figure 5a), which accords with their geographic location in East Arnhem Land. For *D. bilineata* , genetic structure (PCA) and phylogenetic analyses indicated substantial genetic differentiation between all East Arnhem Land samples (from this and previous studies) from the rest of the Top End reference samples of the ETE lineage (50 Bootstrap value) (Figure 5)(Figure S13). In addition, two *D. bilineata* samples from southeast Arnhem Land formed a divergent clade together with previously analysed samples of this species from Groote Eylandt (indicated in light green in Figure 5a). These highly divergent samples were removed from the *D. bilineata* SNP dataset prior to population genetic analyses to ensure the assumptions of the stepping‐stone model (Kimura and Weiss 1964), requirements of EEMS analysis were met.

### 3.5 Genetic Structure and Diversity Within Lineages

#### 3.5.1 *C. amax* ETE

A PCA of *C. amax* (*n* = 38) from the ETE lineage suggested three main genetic clusters, with PCA 1 explaining 13.3% of the variation in the data (Figure 2d). The cross‐validation procedures for sNMF and PopCluster showed highest support for *K* = 3 populations (Figure S17a), corresponding with the PCA clusters: (1) Laynhapuy samples west of Arnhem Bay (‘Laynhapuy 1’) (*n* = 16); (2) Laynhapuy samples north and west of Blue Mud Bay (‘Laynhapuy 2’) (*n* = 12); and (3) SEAL IPA samples (‘SEAL’) (*n* = 10) (Figure 2b–d; Figure 18a). There was significant IBD for *C. amax* across the study region (Mantel test, *r* = 0.7258, *p* < 0.001, Figure S19a) and pairwise fixation index was highest between Laynhapuy 2 and SEAL populations (*F*_ST_ = 0.2470, 95% CI = 0.2357–0.2585, *p* < 0.001) and lowest between Laynhapuy 1 and 2 populations (*F*_ST_ = 0.1530, 95% CI = 0.1448–0.1616, *p* < 0.001) (Table S11).

#### 3.5.2 *C. munda*

A PCA of *C. munda* (*n* = 62) suggested slight genetic differentiation between Laynhapuy and SEAL samples as well as Laynhapuy samples from the Djarrakpi peninsula of Blue Mud Bay, with PC1 explaining 6.1% of the variation (Figure 3d). The cross‐validation procedures for sNMF and PopCluster showed highest support for *K* = 3 and *K* = 2 populations respectively (Figure S17b). The populations identified by sNMF corresponded with the genetic clusters in the PCA, including: (1) samples from the Djarrakpi Peninsula, Laynhapuy IPA (‘Laynhapuy 1’) (*n* = 12); (2) all other Laynhapuy IPA samples (‘Laynhapuy 2’) (*n* = 30); and (3) SEAL IPA samples (‘SEAL’) (*n* = 20) (Figure 3 b–d). PopCluster grouped the Laynhapuy 1 and 2 populations together, supporting divergence between SEAL and Laynhapuy samples, i.e., south and north of Blue Mud Bay as the main feature (Figure S18 b). There was significant IBD for *C. munda* across the study region (Mantel test, *r* = 0.7086, *p* < 0.001, Figure S19 b) and pairwise fixation index was highest between Laynhapuy 1 and SEAL populations (*F*_ST_ = 0.1167, 95% CI =0.1100–0.1238, *p* < 0.001) and lowest between Laynhapuy 2 and SEAL populations (*F*_ST_ = 0.0599, 95% CI = 0.0556–0.0639, *p* < 0.001) (Table S12).

#### 3.5.3 Ct. Quirinus

A PCA of *Ct. quirinus* (*n* = 24) revealed three main genetic clusters, differentiating between the Laynhapuy and SEAL samples and, as for *C. munda*, the Laynhapuy samples from the Djarrakpi peninsula, with PC1 explaining 19.1% of the variation (Figure 4d). The cross‐validation procedures for sNMF and PopCluster showed highest support for *K* = 2 populations (Figure S17c), including: (1) samples north of Blue Mud Bay (‘north’) (*n* = 12), and (2) samples west and south of Blue Mud Bay (‘south’) (*n* = 12) (Figure 4 c,d, Figure S18 c). IBD was significant for *Ct. quirinus* across the study region (Mantel test, *r* = 0.6547, *p* < 0.001,Figure S19c) and pairwise fixation index between the two genetic clusters was F_ST_ = 0.1146 (95% CI = 0.1081–0.1215, *p* < 0.001).

#### 3.5.4 *D. bilineata* ETE Lineage

A PCA of *D. bilineata* samples from the ETE lineage (*n* = 47) again showed support for genetic differentiation between Laynhapuy and the few SEAL samples [from the south‐east limit of the species' distribution (Melville et al. 2019)], and Laynhapuy samples from the Djarrakpi peninsula, with PC1 explaining 6.7% of variation (Figure 5d). The cross‐validation procedures of sNMF and PopCluster showed the highest support for *K* = 1 and *K* = 2 populations, respectively (Figure S17 d). Since *K* = 1 and *K* = 2 often cannot be distinguished (Janes et al. 2017), and PopCluster identified *K* = 2, we also explored *K* = 2 for sNMF as it had a similarly low CV error to *K* = 1. *K* = 2 for sNMF indicated the same two populations as PopCluster, differentiating between the: (1) ‘Laynhapuy’(*n* = 41); and (2) ‘SEAL’ (*n* = 6) IPA samples (Figure 5 c,d, Figure S18 d). Between these two clusters, *F*_ST_ = 0.1845, (95% CI =0.1743,0.19448, *p* < 0.001), and there was significant IBD across the study region (Mantel test, *r* = 0.580, p < 0.001, Figure S19 d).

### 3.6 Effective Migration Estimation

**FIGURE 6:**
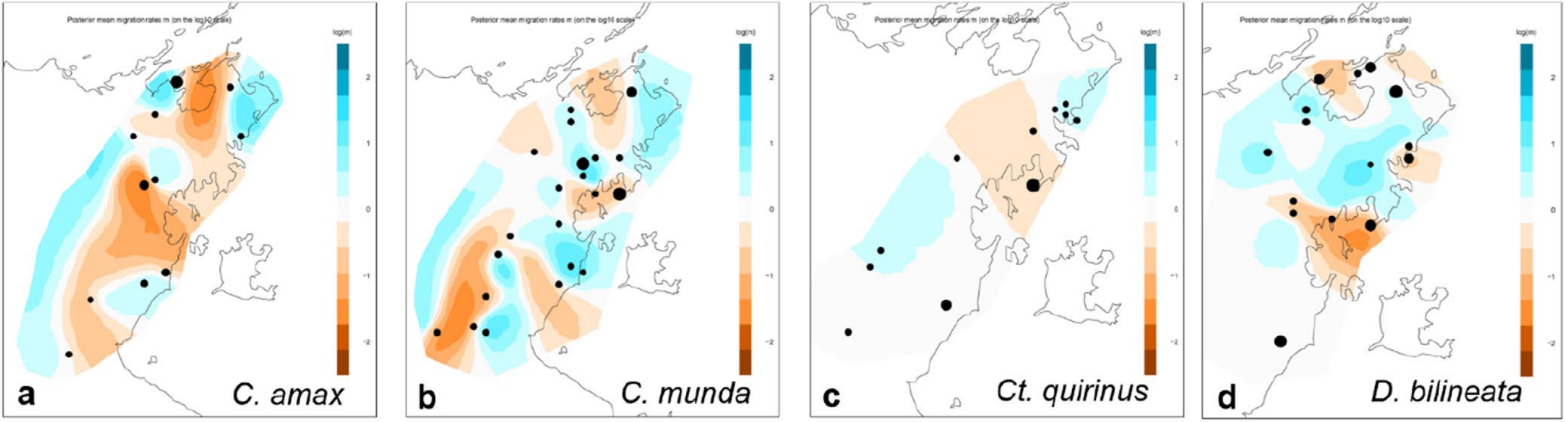
Mapped EEMS estimated effective migration rates for: (a) *C. amax;* (b) *C. munda*, (c) *Ct. quirinus*, and (d) *D. bilineata*. Orange regions indicate estimates of lower‐than‐average and blue regions represent estimates of greater‐than‐average effective migration across the landscape. Black dots represent sample locations, with the size correlated to the number of samples.

To complement the clustering results above, we applied the EEMS methods to provide continuous estimates of spatial variation in effective migration that are comparable across species. As a caveat, we note that several areas of low inferred effective migration have few if any samples. Regions of lower‐than‐average effective migration were indicated across major water bodies such as Arnhem Bay for *C. amax, C. munda* and *D. bilineata* (in the Laynhapuy and Marrthakal IPA) and Blue Mud Bay (Laynhapuy IPA) for all species. These lower‐than‐average regions around Arnhem Bay and Blue Mud Bay extended inland from the coast to varying extents across species and were most evident for *C. amax* (Figure 6a–d). Aside from these regions of lower‐than‐average effective migration that extend inland from the coast, EEMS indicated average or above‐average estimated migration across inland regions for all species, except for a central region of the SEAL IPA for *C. amax* and *C. munda* (Figure 6a,b). Effective migration for *Ct. quirinus* varied the least from average across the sampling region compared to other species (Figure 6a–d).

### 3.7 Cross‐Cultural Knowledge Transfer: Communicating the Results

The Yolŋu dhawurrpunaramirri (two‐way negotiation and discussion) approach identified key Yolŋu Matha (language) concepts that bridged the ontological divide between Yolŋu and Western scientific knowledge systems used to classify animals. Yolŋu social organisation was identified as an overarching source of metaphor to contextualise and explain the results of genetics analyses and bridge the divide in Yolŋu and Western scientific fauna classification in a culturally grounded way. Specifically, the Yolŋu concept of gurrkurr (venous system, and by metaphorical extension root system and ‘blood line’ or ancestry) emerged as a key metaphor to discuss phylogenetic trees (Campbell et al. 2025). Yolŋu terms: miṯtji (group of people), mala (group) and bäpurru (clan) were discussed to explain the Western concept of species, as there is no direct analogue for species in Yolŋu Matha (Campbell et al. 2025). Yolŋu animal classifications can be described as relational, totemic and ecologically embedded (Campbell et al. 2024, 2025). Yolŋu Yirralka Rangers and knowledge holders were pleased to be fed back the results of the genetics analyses and actively contributed to their contextualisation so they could be meaningfully communicated to other members of society. This part of the project has been reported in greater detail in Campbell et al. (2025).

## 4 Discussion

Our results provide the most detailed molecular evidence of diversity of terrestrial vertebrates available for East Arnhem Land, spanning the SEAL, Laynhapuy and Marthakal IPAs, at a species and lineage level. We obtained substantial new samples from collaborative field work, applied genetic methods to improve identifications in morphologically challenging genera of common lizards (*Carlia, Ctenotus* and *Diporiphora*) and generated 93 new occurrence records for species for the three IPAs by combining new evidence with prior genetic data from across northern Australia. We provide one of the first assessments, albeit preliminary, of genetic structuring of populations of terrestrial species in this highly diverse region of northern Australia. Across four species, our results show potential divergence of populations between the SEAL and Laynhapuy IPAs, north and south of Blue Mud Bay. Although sparse and non‐uniform sampling could be shaping this apparent divergence, we suggest if it is supported by future sampling the landscape features of this region could limit gene flow. We also provide one of the first examples of collaborative efforts to contextualise results of genetic research conducted in northern Australia with Indigenous research partners that respect Indigenous priorities for two‐way knowledge sharing published in academic literature (Campbell et al. 2025). These results are informative for the ongoing cross‐cultural monitoring and conservation of reptile fauna in Arnhem Land and were only made possible through collaborations with Indigenous Ranger groups in mutually beneficial fauna monitoring activities. Our research answers international calls for collaborative, mutually beneficial conservation practice with Indigenous research partners United Nations (UN) 2007; Garnett et al. 2018; Intergovernmental Science‐Policy Platform on Biodiversity and Ecosystem Services (IPBES 2019; Mc Cartney et al. 2023).

### 4.1 New and Revised Species Records

This project greatly increased the occurrence records and understanding of the distribution of *Carlia, Ctenotus*, and *Diporiphora* species in East Arnhem Land. For the Laynhapuy IPA we contributed the first scientifically published records of: four species of *Carlia*; four species of *Ctenotus*; and one species of *Diporiphora*. For the Marrthakal IPA we provide evidence that the ECI lineage of *C. amax*, identified as endemic to the English Company Islands by Potter et al. (2016), actually extends onto the mainland (Table 2, Figure S14). Our results also expanded the limited occurrence records of three species of *Carlia*, two species of *Ctenotus*, and one species of *Diporiphora*, previously recorded in the Laynhapuy‐Marthakal shared IPA region, and added records of two previously recorded species of *Carlia*, three species of *Ctenotus*, and one species of *Diporiphora* in the Marrthakal IPA (Table 2, Figure S14–S16). For the SEAL IPA, we provide the first records for three *Ctenotus* species and we add to limited previous sampling of two *Carlia*, two *Ctenotus*, and three *Diporiphora* species (Table 2, Figure S14–S16).

Our phylogenetic and genetic structure analyses were in some cases discordant from previous studies, revealing novel patterns of genetic structure in East Arnhem. We revealed that *C. amax* and *D. bilineata* in the Laynhapuy and Marthakal IPAs were genetically distinct from previously analysed samples of the respective ETE lineages from southeast Arnhem Land and the rest of the Top End as noted in Potter et al. (2016) and Fenker et al. (2021). The present *C. sexdentata* results differ from previous studies, likely because of limited taxon sampling. *C. sexdentata* is proposed to be two genetically divergent species (as in Figure 2, Bragg et al. 2018), with the Top End taxon (species) being more closely related to Papua New Guinea members of the *Ct*. *fusca* group (CM, *unpublished data*). We provide evidence for the need to taxonomically revise *Ct. quirinus* in the context of the *Ct. essingtonii* species complex, adding another species to the many across mammals, amphibians and reptile taxa across northern Australia requiring taxonomic revision following genetic analyses (Melville et al. 2019; Eldridge and Potter 2020; Catullo and Keogh 2021; Prates et al. 2023). We also provide the first molecularly confirmed record of *Ct. quirinus* from Elcho Island which appears genetically distinct from mainland and Groote Eylandt *Ct. quirinus* samples. *Ct. vertebralis* and *Ct. astictus* are tentative identifications. Morphological identification with voucher specimens alongside further molecular analyses is needed to disentangle taxonomic uncertainty.

Continued cross‐cultural sampling across Central and Western Arnhem Land (e.g., Arafura Swamp IPA and Mimal IPA), and the eastern margin of the Arnhem plateau (Warddeken IPA and Djelk IPA) is opportune. Genetic sampling here could allow for continued genomic efforts to better understand larger scale phylogeographic structure, its potential predictors, and the uniqueness of reptile taxa in this region. Application of our collaborative cross‐cultural survey practice in these IPAs could simultaneously deliver socio‐cultural benefits for Indigenous communities through incorporation of local aspirations and priorities (see Ens et al. 2016; Lilleyman et al. 2022; Campbell 2025).

### 4.2 Genetic Diversity Between Populations: North–South Genetic Differentiation

Our phylogenetic and population structure results suggest a potential restriction in gene flow driving a north–south genetic differentiation in reptile taxa around Blue Mud Bay. Similarly, the EEMS plots indicated lower than average connectivity around the Blue Mud Bay region across all species, albeit with variation in intensity and coverage (Figure 6a–d). However, this potential differentiation could be caused by the uneven sampling design arising from the co‐design process and logistical constraints. Increased sampling in northern SEAL IPA and southern Laynhapuy IPA to fill gaps in the current sampling could clarify any divergence in gene flow in this region. Most species showed strong (> 0.6) IBD, except for *D. bilineata* , which had the greatest gap in sampling between the Laynhapuy and SEAL IPA. Further, *F*_*ST*_ for Laynhapuy 2 and SEAL populations of *C. munda*, the most well‐sampled species, was low (< 0.1), suggesting high connectivity, despite the population structure results.

Nevertheless, if further sampling provides evidence for a north–south divergence, we note that *C. amax, C. munda, Ct. quirinus*, *and D. bilineata* are each associated with drier microhabitats, so the extensive floodplains and mangrove systems of Blue Mud Bay could be driving a north–south genetic differentiation. The south end of Blue Mud Bay also aligns not only with IPA but Australian bioregion boundaries (IBRA Version 7, [Commonwealth of Australia, Department of Climate Change, Energy, Environment and Water (DCCEEW) 2024)], marking the transition from ‘Arnhem coast’ to ‘Central Arnhem.’ If this genetic differentiation exists, it could align with a larger shift in climate, alongside geology, landforms and native vegetation that are used to determine bioregions.

### 4.3 Management Implications

*Carlia, Ctenotus* and *Diporiphora* are extremely difficult to identify in the field using morphological keys and local Yolŋu classifications, which do not delineate between similar‐looking groups. We provide the finest‐scale detail of the distribution of these species, which will increase the accuracy of future field identifications of species and contribute to enhanced understanding of the biodiversity value of these three IPAs. Conveniently for monitoring and management purposes, the north–south population divide around Blue Mud Bay, if confirmed with more sampling, aligns approximately with the boundary between the Laynhapuy and SEAL IPA management areas. Areas of inferred low population connectivity between the Laynhapuy and Marthakal IPAs also align with the Laynhapuy and Marthakal IPA joint management region on the northeast coastline of the mainland. Knowledge of these spatial separations and overlaps of species between management zones may encourage greater collaboration between IPAs and continued research of not only reptiles but also other species groups of local interest or concern.

By generating one of the highest‐resolution genomic datasets of any terrestrial fauna taxa in East Arnhem Land, the results of this study may provide insights into the expected population structure and connectivity of other low‐dispersal terrestrial species in this region for which sampling is incomplete or challenging, especially culturally significant species (see Campbell et al. 2024). Our results highlight the benefit of co‐analysing new samples with pre‐existing SNP datasets from across northern Australia that have undergone previous phylogenetic analysis. Such datasets now also exist for other species; therefore, there remains potential for expanding this approach across other taxa across northern Australia, including species with greater cultural significance, such as goannas, rock wallabies and quolls. This approach can also be applied nationally and internationally across other regions where similar extensive data gaps across Indigenous owned and/or managed lands exist.

### 4.4 Cross‐Cultural Research Approach

The present genetics study was part of a broader cross‐cultural collaboration (Ens et al. 2016; Daniels et al. 2022; Campbell et al. 2024; Campbell 2025) that aimed to fill biodiversity knowledge gaps in data‐poor regions of East Arnhem Land, advancing cross‐cultural partnerships, informing biodiversity conservation strategies and building two‐way knowledge and capacity of local Indigenous Ranger groups and researchers. Throughout the broader collaboration, researchers and rangers co‐adapted fauna sampling methods to negotiate mutually beneficial outcomes (Moritz et al. 2013; Ens et al. 2016; Campbell 2025; Campbell et al. 2025). This included embedding place‐based intergenerational and cross‐cultural knowledge transfer, with a focus on both Indigenous and Western animal classification and biocultural knowledge into the survey camp design. Indigenous‐led survey and sample site selection, as a means of reconnecting with and transferring knowledge of remote and culturally significant places, was also a key priority of Indigenous research partners (Ens et al. 2016).

Cross‐cultural two‐way knowledge sharing involved engaged and meaningful communication of the genetic results (aim 4) from researchers to Indigenous Rangers and community members. Here, and in Campbell et al. (2025) in greater detail, we demonstrate how local Indigenous research approaches can bridge ontological divides needed to address mutual incomprehension and work towards ethical two‐way knowledge sharing in cross‐cultural fauna research. Such approaches are especially important when knowledge systems are founded upon different worldviews and literal translation is not possible or meaningful, and where Indigenous research partners are not engaged in all steps of the project; here, the genetic analyses (Campbell et al. 2025).

Bridging the divide between different worldviews and languages is complex, and in this context was made possible through a trusting long‐term relationship between researchers and rangers. Nevertheless, equitable knowledge sharing should remain an aspiration for cross‐cultural researchers and wildlife ecologists more broadly, in line with recommendations for respectful and inclusive collaboration in conservation genetics projects both in Australia (Shaw et al. 2024; Campbell et al. 2025) and globally (Mc Cartney et al. 2022; Rayne et al. 2022), and in Indigenous‐led research protocols (AIATSIS 2020). Priorities of project co‐development cross‐cultural two‐way learning set the tone for an organic, negotiated, exploratory research approach. Research questions were not defined a priori by Western scientists but co‐developed together during the research practice. This resulted in a sampling design that was not uniform for all species and needs to be considered when interpreting results and inferences about gene flow and landscape connectivity that can be made from them, as we have expressed above. Further, because of the limited or non‐existent sampling of East Arnhem Land, this research was inherently exploratory in its approach from a Western science perspective and not in conflict with ethical co‐design protocol.

## 5 Conclusion

Here, we demonstrate how cross‐cultural fauna surveys can enhance understanding of reptile diversity across remote, Indigenous‐owned lands through the application of next‐generation sequencing and co‐analyses of SNP datasets. Further sampling across Indigenous‐owned and managed lands in Arnhem Land and the Top End could fill gaps in current sampling and would enhance the understanding of population structure and landscape genetics inferences that can be drawn from the results presented here. Engaging in cross‐cultural two‐way learning approaches can fulfil local Indigenous aspirations for ethical conservation practice. Thus, we recommend collaborative cross‐cultural surveys co‐led by researchers and Indigenous Rangers across Indigenous‐owned or managed, data‐poor regions of Australia, and beyond to better understand and support the biocultural diversity of Indigenous‐owned lands (Garnett et al. 2018; IPBES 2019; Mc Cartney et al. 2023). Further application of this approach to threatened and culturally significant species (Collier‐Robinson et al. 2019; Campbell et al. 2024) could enhance cross‐cultural benefits and help safeguard the interdependent biological and cultural diversity currently at risk globally (Maffi and Woodley 2012).

## Author Contributions

**Bridget L. Campbell:** conceptualization (supporting), data curation (lead), formal analysis (lead), funding acquisition (supporting), investigation (equal), methodology (equal), software (lead), visualization (lead), writing – original draft (lead). **Yirralka Rangers:** funding acquisition (supporting), investigation (equal), methodology (equal), resources (equal), writing – review and editing (supporting). **Yugul Mangi Rangers:** investigation (equal), methodology (supporting), resources (supporting). **Numburindi Rangers:** investigation (equal), methodology (supporting), resources (supporting). **Learning on Country – Laynhapuy Homelands School:** investigation (supporting). **Learning on Country – Shepherdson College:** investigation (supporting). **Rachael Y. Dudaniec:** conceptualization (equal), methodology (equal), supervision (equal), validation (equal), writing – review and editing (equal). **Craig Moritz:** conceptualization (equal), funding acquisition (supporting), investigation (supporting), methodology (equal), supervision (supporting), writing – review and editing (equal). **Jéssica Fenker:** data curation (equal), methodology (supporting), resources (equal), validation (supporting), writing – review and editing (supporting). **Emilie Ens:** conceptualization (equal), funding acquisition (lead), investigation (equal), methodology (equal), project administration (equal), supervision (equal), writing – review and editing (equal).

## Funding

This work was supported by Macquarie University, Higher Degree Research student fund, Nature Conservancy, Australian Academy of Science, Max Day Environmental Science Fellowship 2022. Department of Industry and Science, Australian Government, Citizen Science Grant CSG56008. Australian Research Council, ARC Linkage Project (2020) (LP200301589) with indu. Australian Government, Research Training Program.

## Conflicts of Interest

The authors declare no conflicts of interest.

## Supporting information


**Data S1:** ece373304‐sup‐0001‐supinfo.docx.

## Data Availability

Data and code used in these analyses are available via the Open Science Framework project link below for the review process. https://osf.io/eyw8j/?view_only=06b2a56ad7904fb7b750877d16641573. The BC (Biocultural) Notice is a visible notification that there are accompanying cultural rights and responsibilities that need further attention for any future sharing and use of this material or data. The BC Notice recognises the rights of Indigenous Peoples to permission the use of information, collections, data and digital sequence information (DSI) generated from the biodiversity or genetic resources associated with traditional lands, waters, and territories. Following publication, data will be available, subject to permission from Indigenous data custodians. Access can be requested via the Open Science Framework platform here: doi 10.17605/osf.io/eyw8j 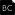.
