# Supplementary material for: Collaborative Conservation Genetics of Cryptic Reptile Taxa From Northern Australia
Source: Ecol Evol. 2026 Apr 9;16(4):e73304. doi: 10.1002/ece3.73304 (PMC13067160; doi:10.1002/ece3.73304)
Supplement: Supplementary file 1 — Data S1: ece373304‐sup‐0001‐supinfo.docx. [file ECE3-16-e73304-s001.docx]

# Supplementary Material: Collaborative conservation genetics of cryptic reptile taxa from Northern Australia

Bridget L. Campbell^1^, Yirralka Rangers^2^, Yugul Mangi Rangers^3^, Numburindi Rangers^3^, Learning on Country - Shepherdson College^4^, Rachael Y. Dudaniec^1^, Craig Moritz^5^, Jéssica Fenker^6^, Emilie Ens^1^

1. School of Natural Sciences, Macquarie University, Sydney, Australia.
2. Laynhapuy Aboriginal Corporation, Yirrkala, Northern Territory, Australia.
3. South East Arnhem Land Indigenous Protected Area, Northern Territory, Australia.
4. Galiwinku, Elcho Island, Northern Territory, Australia.
5. Research School of Biology, Australian National University, Canberra, Australia.
6. Museums Victoria Research Institute, Museums Victoria, Melbourne, Australia.

**Corresponding author:** Bridget Campbell, [bridget.campbell@mq.edu.au](mailto:bridget.campbell@mq.edu.au)

# Text

## S1. Study region information

The present study was undertaken across three IPAs in East Arnhem Land that span around ~37,000 km2. Arnhem Land was declared as Aboriginal Land under the Commonwealth’s Aboriginal Land Rights (Northern Territory) Act (1976) and has been stewarded by Indigenous traditional custodians since time immemorial. The Marthakal IPA (~3,230 km2) declared in 2016, is managed by the Gumurr Marthakal Rangers and spans the Elcho Island Group, Wessel Islands, English Company Islands and nearby mainland regions around Arnhem and Buckingham Bay. The Laynhapuy IPA (~14,728 km2), declared in 2006, is managed by the Yirralka Rangers, and includes overlapping management regions with the Gumurr Marthakal, and spans from islands north of the mainland to the bottom of the Blue Mud Bay region. The SEAL IPA (~ 19,000 km2) is managed by the Yugul Mangi and Numburindi Rangers and is located on the western Gulf of Carpentaria on the Northern Territory mainland. It spans from a shared management zone with the Laynhapuy IPA in the north around the Blue Mud Bay region, down to the Roper River. These IPAs were declared following direction of Indigenous Elders and leaders who articulated their customary obligation to care for their ancestral estates, and contemporary want to engage with Western science and technology to manage introduced threats.

This region of East Arnhem Land is dominated by tropical eucalypt savanna woodlands, intersected by pockets of paperbark (melaleuca) forests, swamps, rocky escarpments, and edged by coastal dune systems and large floodplains. This region includes internationally significant wetlands for migratory species and is habitat for a range of threatened species, including on pest-free island refuges (Yirralka Rangers 2017). The region spans four Australian bioregions and is typified by low topographic relief, excluding the sandstone escarpment in the southwest. Rainfall decreases from the northern coast towards the arid centre of Australia (Woinarski et al. 2007). The climate is monsoonal with high temperatures year-round and frequent cyclones in the wet season, when biodiversity sampling is generally unfeasible.

## S2. Diversity Arrays Technology Sequencing

SNPs were identified and genotyped using the DArTseq method based on combining complexity reduction and next-generation sequencing methods (see Jaccoud et al. 2001). *Carlia* and *Diporiphora* samples were sequenced using DArTseq low-density genotyping protocol (0.8 mln reads), and high-density genotyping protocol (1.2 mln reads) was used to sequence *Ctentous* samples, given greater genome size. Several restriction enzyme combinations were tested to determine restriction enzymes that could effectively select and digest low-copy genomic fragments for each genus. Fragments were amplified using PCR and sequenced using Illumina Hiseq2500. The National Centre for Biotechnology Information (NCBI) database was used to search for matches with bacterial, fungal and human sequences to detect and remove cross-contaminated data. Following this removal, DArTsoft14 software was used to call SNPs on the remaining sequences (Jaccoud et al. 2001). A series of proprietary filters were then applied to select reliable SNP markers. This included testing for Mendelian distribution of alleles, to ensure that true allelic variants from the paralogous sequences were called. After proprietary filtering, DArT provided the resultant biallelic SNP dataset with metadata for each locus.

## References

Fenker, J., Tedeschi L.G., Melville, J. & Moritz, C. 2021. Predictors of phylogeographic structure among codistributed taxa across the complex Australian monsoonal tropics. Molecular ecology 30:4276-4291.

Jaccoud, D., Peng, K., Feinstein, D. & Kilian, A. (2001). Diversity Arrays: a solid state technology for sequence information independent genotyping. Nucleic Acids Research, 29, e25-e25.

Jolly, C., Schembri, B. & Macdonald, S. (2023). Field Guide to the Reptiles of the Northern Territory. CSIRO Publishing, Collingwood, Australia.

Melville, J., Smith, K., Horner, P. & Doughty, P. (2019). Taxonomic revision of dragon lizards in the genus' Diporiphora'(Reptilia: Agamidae) from the Australian monsoonal tropics. Memoirs of Museum Victoria, 78, 23-55.

Potter, S., Bragg, J.G., Peter, B.M., Bi, K. & Moritz, C. (2016). Phylogenomics at the tips: inferring lineages and their demographic history in a tropical lizard, Carlia amax. Molecular Ecology, 25, 1367-1380.

Rosauer, D.F., Blom, M. P. K., Bourke, G., Catalano, S., Donnellan, S., Gillespie, G., Mulder, E., Oliver, P. M., Potter, S., Pratt, R. C., Rabosky, D. L., Skipwith, P. L. & Moritz, C. (2016). Phylogeography, hotspots and conservation priorities: an example from the Top End of Australia. Biological conservation, 204, 83-93.

Woinarski, J., Mackey, B., Nix, H. & Traill, B. (2007). The nature of northern Australia: its natural values, ecological processes and future prospects. ANU Press, Canberra, Australia.

Yirralka Rangers. 2017. Laynhapuy Indigenous Protected Area Management Plan (2017-2022). Laynhapuy Homelands Aboriginal Corporation, Yirrkala, Northern Territory.

# Tables

**Table S1.** Filtering parameters and results of the *Carlia* genus-level dataset.

| Filter parameters | SNP count | Ind Count |
| --- | --- | --- |
| Unfiltered | 59302 | 338 |
| Monomorphs | 59128 | 338 |
| Reproducibility 90% | 59128 | 338 |
| Depth of Coverage (lower =8, upper =60) | 41516 | 338 |
| Loci by Individual (20%) | 41516 | 336 |
| Loci by Callrate (60%) | 13316 | 336 |
| Minor Allele Frequencines (0.02) | 7672 | 336 |
| Filter Secondaries | 5399 | 336 |

| Filter parameters | SNP count | Ind count |
| --- | --- | --- |
| Unfiltered | 157563 | 38 |
| Monomorphs | 60212 | 38 |
| Reproducibility 90% | 60212 | 38 |
| Depth of Coverage (lower =10, upper=60) | 33981 | 38 |
| Individual by Callrate (20%) | 33981 | 38 |
| Loci by Callrate (80%) | 19556 | 38 |
| Minor Allele Frequency (0.02) | 19556 | 38 |
| Filtering Secondaries | 14061 | 38 |
| Hardy-Weinberg Equilibrium (HWE) (p=0.001) | 9230 | 38 |
| Linkage Disequilibrium | 5429 | 38 |

**Table S2.** Filtering parameters and results from *C. amax*.

**Table S3.** Filtering parameters and results from *C. munda*.

| Filter parameters | SNP count | Ind count |
| --- | --- | --- |
| Unfiltered | 203759 | 63 |
| Monomorphs | 107444 | 63 |
| Reproducibility 90% | 107444 | 63 |
| Depth of Coverage (lower=10, upper=60) | 53960 | 63 |
| Individual by Callrate (20%) | 53960 | 62 |
| Loci by Callrate (80%) | 27451 | 62 |
| Minor Allele Frequency (0.02) | 16686 | 62 |
| Filtering Secondaries | 12141 | 62 |
| Hardy-Weinberg Equilibrium (HWE) (p=0.001) | 5865 | 62 |
| Linkage Disequilibrium | 5361 | 62 |

| Filter parameters | SNP count | Ind Count |
| --- | --- | --- |
| Unfiltered | 223037 | 86 |
| Monomorphs | 222440 | 86 |
| Reproducibility 90% | 222440 | 86 |
| Depth of Coverage (lower= 10, upper=50) | 116935 | 86 |
| Loci by Individual (20%) | 116935 | 86 |
| Loci by Callrate (60%) | 21435 | 86 |
| Minor Allele Frequencines (0.02) | 17180 | 86 |
| Filter Secondaries | 12448 | 86 |

**Table S4.** Filtering parameters and results of the *Ctenotus* genus-level SNP dataset.

**Table S5.** Filtering parameters and results of the *Ct. quirinus*.

| Filter parameters | SNP count | Ind count |
| --- | --- | --- |
| Unfiltered | 223037 | 24 |
| Monomorphs | 15963 | 24 |
| Reproducibility 90% | 15963 | 24 |
| Depth of Coverage (lower =10, upper=60) | 8557 | 24 |
| Individual by Callrate (20%) | 8557 | 24 |
| Loci by Callrate (80%) | 6852 | 24 |
| Minor Allele Frequency (0.02) | 6852 | 24 |
| Filtering Secondaries | 6473 | 24 |
| Hardy-Weinberg Equilibrium (HWE) (p=0.001) | 5056 | 24 |
| Linkage Disequilibrium | 3140 | 24 |

**Table S6.** Filtering parameters and results of the *Diporiphora* genus-level SNP dataset.

| Filter parameters | SNP count | Ind count |
| --- | --- | --- |
| Unfiltered | 80865 | 155 |
| Monomorphs | 55956 | 155 |
| Reproducibility 90% | 55956 | 155 |
| Depth of Coverage (lower =8, upper=20) | 24723 | 155 |
| Loci by Individual (20%) | 33337 | 148 |
| Loci by Callrate (60%) | 4923 | 148 |
| Minor Allele Frequency (0.02) | 2637 | 148 |
| Filtering Secondaries | 1758 | 148 |

**Table S7.** Filtering results for *D. bilineata.*

| Filter parameters | SNP count | Ind count |
| --- | --- | --- |
| Unfiltered | 141406 | 47 |
| Monomorphs | 79718 | 47 |
| Reproducibility 95% | 79718 | 47 |
| Depth of Coverage (lower =8, upper=20) | 28061 | 47 |
| Loci by Callrate (85%) | 21645 | 47 |
| Minor Allele Frequency (0.02) | 15996 | 47 |
| Filtering Secondaries | 11687 | 47 |
| HWE (0.01) | 9571 | 47 |
| Linkage Disequilibrium | 8504 | 47 |

**Table S8.** Field ID of *Carlia, Ctenotus* and *Diporiphora* samples sequenced and analysed using ddRAD from this study and C.M. and J.F. reference samples.

|  | Current study | C.M. ref | J.F. ref |
| --- | --- | --- | --- |
| Carlia |  |  |  |
| Carlia sp. | 11 | 2 | 0 |
| C. amax | 46 | 18 | 95 |
| C. gracilis | 0 | 3 | 28 |
| C. munda | 67 | 5 | 50 |
| C. rufilatus | 1 | 7 | 1 |
| C. sexdentata* | 4 | 19 | 0 |
| Total | **129** | **54** | **174** |
| Ctenotus |  |  |  |
| Ctenotus sp. | 33 | 0 | NA |
| Ct. astictus* | 4 | 0 | NA |
| Ct. essingtonii | 6 | 10 | NA |
| Ct. quirinus | 1 | 1 | NA |
| Ct. inornatus | 10 | 2 | NA |
| Ct. robustus | 19 | 2 | NA |
| Ct. spaldingi | 1 | 3 | NA |
| Ct. superciliaris | 0 | 2 | NA |
| Ct. vertebraḻis* | 6 | 0 | NA |
| Total | **70** | **20** | **NA** |
| Diporiphora |  |  |  |
| Diporiphora sp. | 5 | 0 | 9 |
| D. bilineata | 46 | 10 | 54 |
| D. gracilis | 0 | 0 | 2 |
| D. granulifera | 0 | 0 | 7 |
| D. magna | 0 | 0 | 41 |
| D. margateyae | 0 | 0 | 3 |
| D. lalliae | 3 | 0 | 0 |
| D. albilabris | 0 | 0 | 13 |
| D. bennetti | 0 | 0 | 5 |
| D. perplexa | 0 | 0 | 19 |
| D. sobria | 0 | 0 | 39 |
| Total | **54** | **10** | **192** |

*indicates species identification is tentative and requires voucher specimens.

**Table S9.** *Ctenotus* sp. reference samples from C.M. and their respective molecular identity and reference in literature.

| Sample reference ID | Field species ID | Molecular species ID and lineage in previous study | Reference to previous study |
| --- | --- | --- | --- |
| CCM5477 | *spaldingi* | *spaldingi NE* | Prates et al 2022, 2023 |
| CCM2866 | *robustus* | *robustus NW* | Prates et al 2022, 2023 |
| CCM2518 | *vertebralis* | *superciliaris EN* | Prates et al 2022, 2023 |
| CCM4920 | *essingtonii* | *inornatus N* | Prates et al 2022, 2023 |
| CCM2497 | *spaldingi* | *spaldingi NE* | Prates et al 2022, 2023 |
| CCM4016 | *spladingi* | *spaldingi NE* | Prates et al 2023 |
| CCM0956 | *robustus* | *robustus NW* | Prates et al 2022, 2023 |
| CCM2565 | *vertebralis* | *superciliaris EN* | Prates et al 2022, 2023 |
| CCM3712 | *quirinus* | *quirinus* | Rosauer et al 2016 |

**Table S10.** Population genetics statistics for *D. bilineata* (K=2), *C. amax* (K=3), *C. munda* (K=3) and *Ct. quirinus* genetic clusters identified by sNMF*.*

| Species | Genetic clusters | Number of individuals (n) | F_IS_ |
| --- | --- | --- | --- |
| D. bilineata | North | 41 | 0.2673 |
|  | South | 6 | 0.2360 |
| C. amax | Laynhapuy 1 | 15 | 0.3643 |
|  | Laynhapuy 2 | 13 | 0.4805 |
|  | SEAL | 10 | 0.4085 |
| C. munda | Laynhapuy 1 | 12 | 0.1841 |
|  | Laynhapuy 2 | 31 | 0.2817 |
|  | SEAL | 19 | 0.3331 |
| Ct. quirinus | North | 12 | 0.3518 |
|  | South | 12 | 0.3264 |

F_IS_= Inbreeding coefficient.

**Table S11.** Pairwise *F_ST_* for K=3 genetic clusters of *C. amax* identified via sNMF (WC; 1000 iterations and 95% CI)*.*

|  | Laynhapuy 2 | SEAL |
| --- | --- | --- |
| Laynhapuy 1 | 0.1530 | 0.2337 |
| SEAL | 0.2470 | NA |

**Table S12.** Pairwise *F_ST_* for K=3 genetic clusters of *C. munda* identified via sNMF (WC; 1000 iterations and 95% CI)*.*

|  | Laynhapuy 2 | SEAL |
| --- | --- | --- |
| Laynhapuy 1 | 0.0890 | 0.1167 |
| SEAL | 0.0599 | NA |

## References

Prates, I., Hutchinson, M.N., Singhal, S., Moritz, C. & Rabosky, D.L. 2023. Notes from the taxonomic disaster zone: Evolutionary drivers of intractable species boundaries in an Australian lizard clade (Scincidae: Ctenotus). Molecular ecology, 00, 1-25.

Prates, I., Singhal, S., Marchán-Rivadeneira, M.R., Grundler, M.R., Moritz, C., Donnellan, S.C. & Rabosky, D.L. (2022). Genetic and Ecogeographic Controls on Species Cohesion in Australia’s Most Diverse Lizard Radiation. The American naturalist, 199, E57-E75.

Rosauer, D.F., Blom, M. P. K., Bourke, G., Catalano, S., Donnellan, S., Gillespie, G., Mulder, E., Oliver, P. M., Potter, S., Pratt, R. C., Rabosky, D. L., Skipwith, P. L. & Moritz, C. (2016). Phylogeography, hotspots and conservation priorities: an example from the Top End of Australia. Biological conservation, 204, 83-93.

# Figures

| 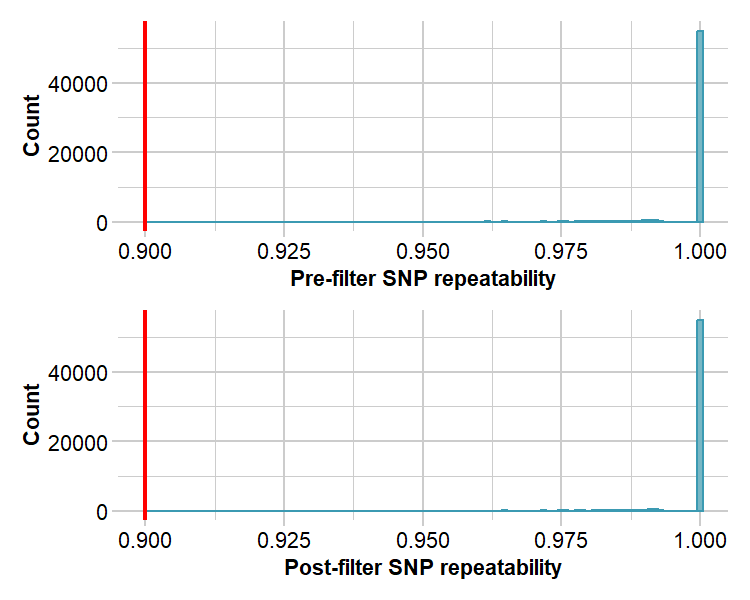  **a)** | **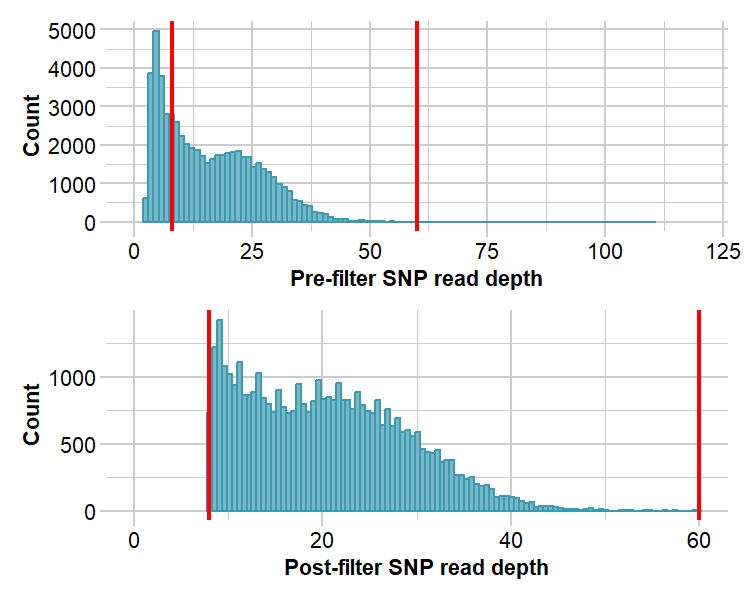**  **b)** |
| --- | --- |
| **c)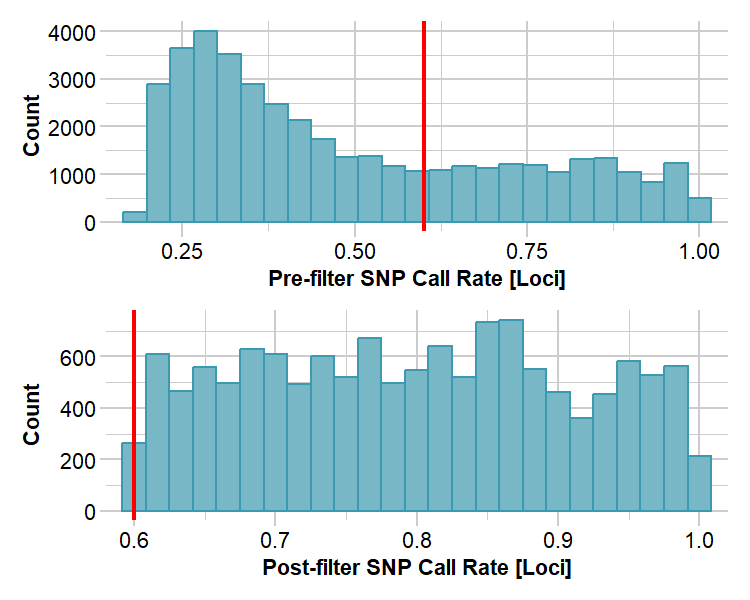** | **d)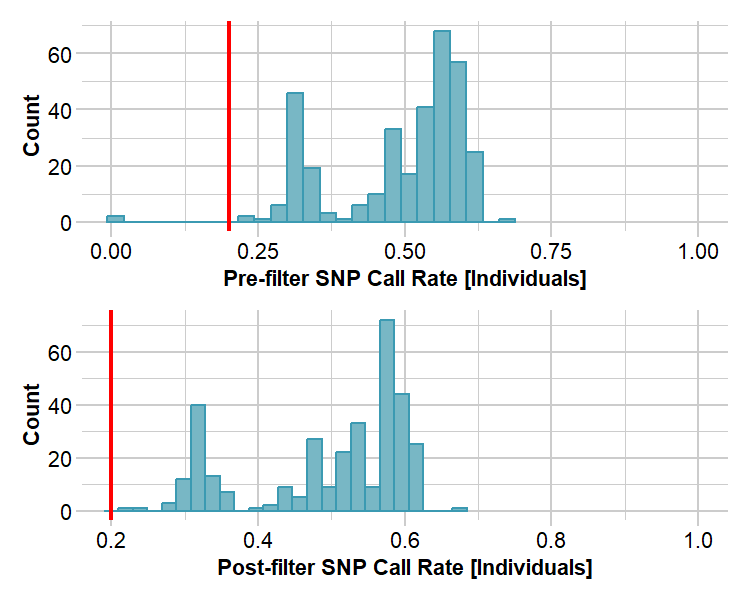** |
| **e)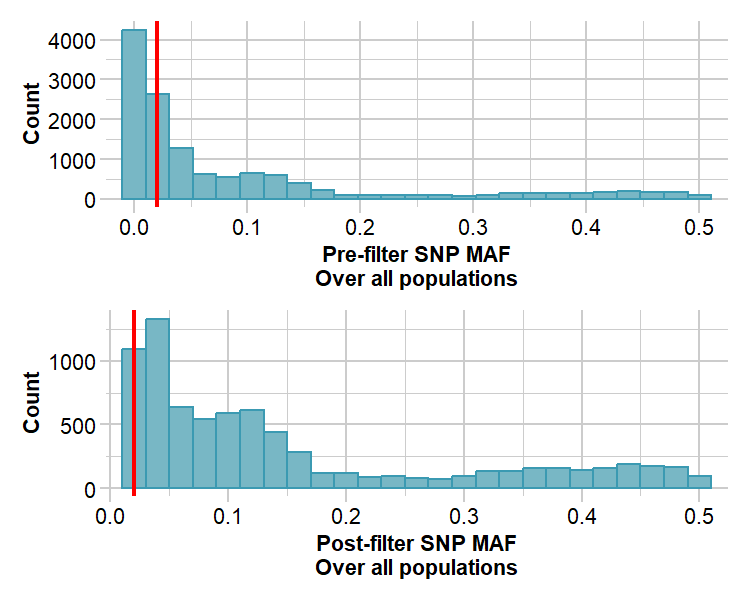** | **f)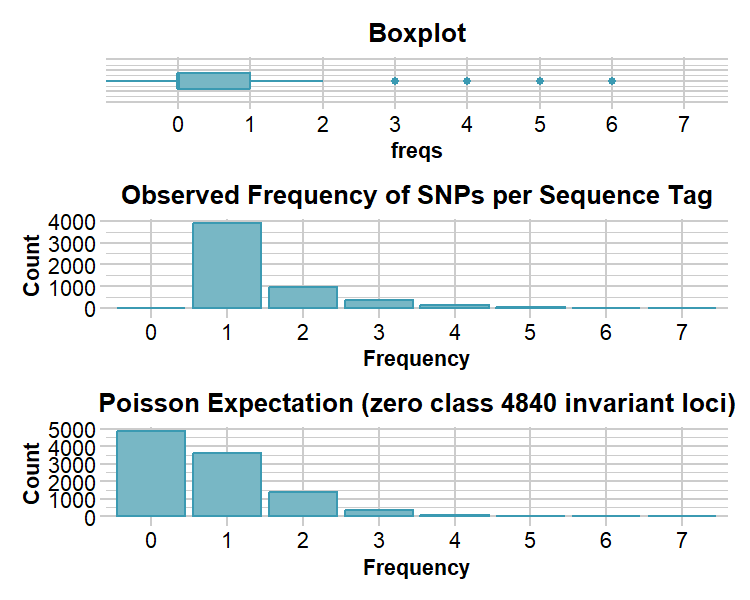** |
| **Figure S1.** *Carlia* genus-level dataset filtering results of: a) reproducibility (≥90%); b) read depth (8 ≥ ≤60); c) call rate by loci (≥0.60); d) call rate by individual (≥0.20); e) minor allele frequency (≥0.02) and f) the report summary of indicating secondaries (SNPs that share a sequence tag and are likely linked) that were removed from analyses. Red bars represent the filter thresholds. | |

| **a)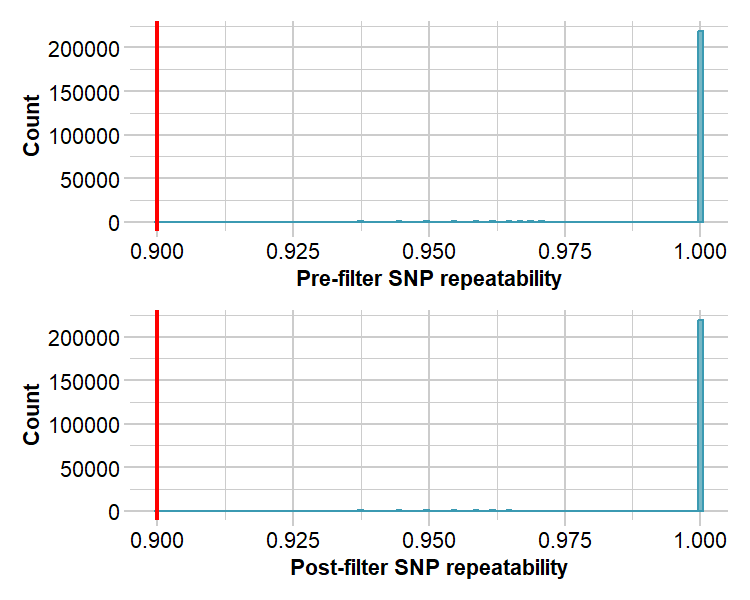** | **b)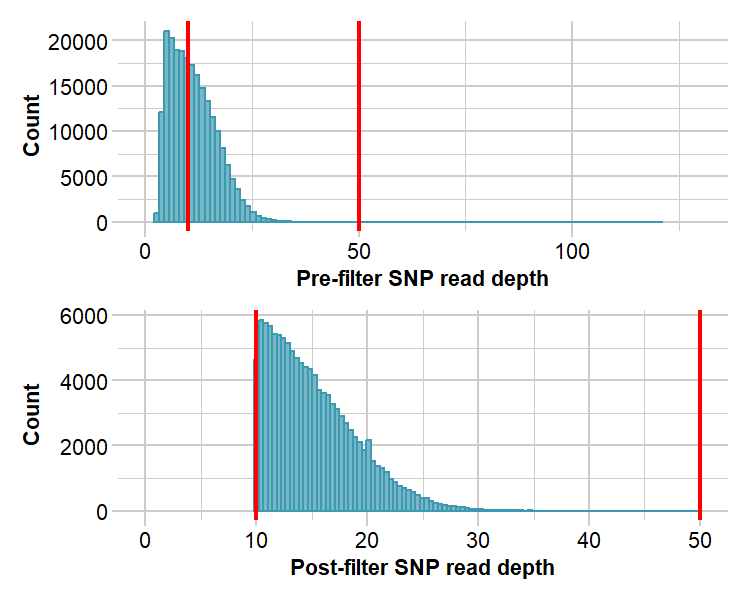** |
| --- | --- |
| **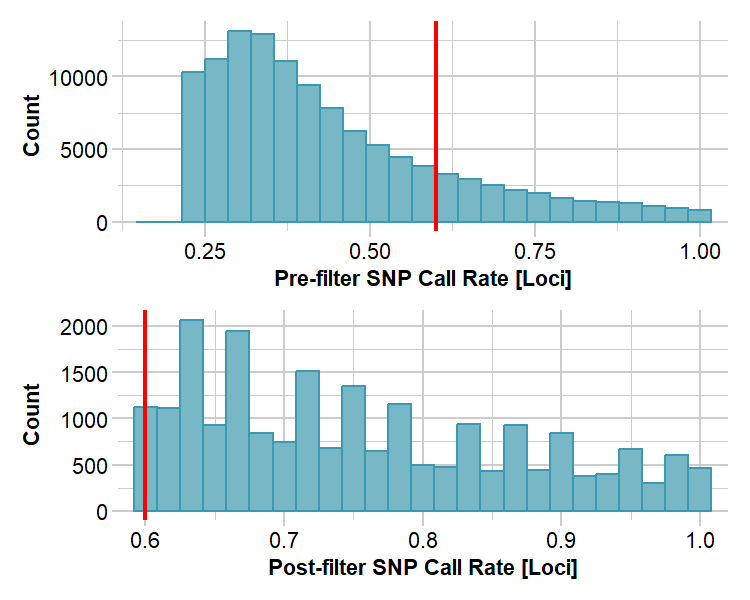**  **c)** | **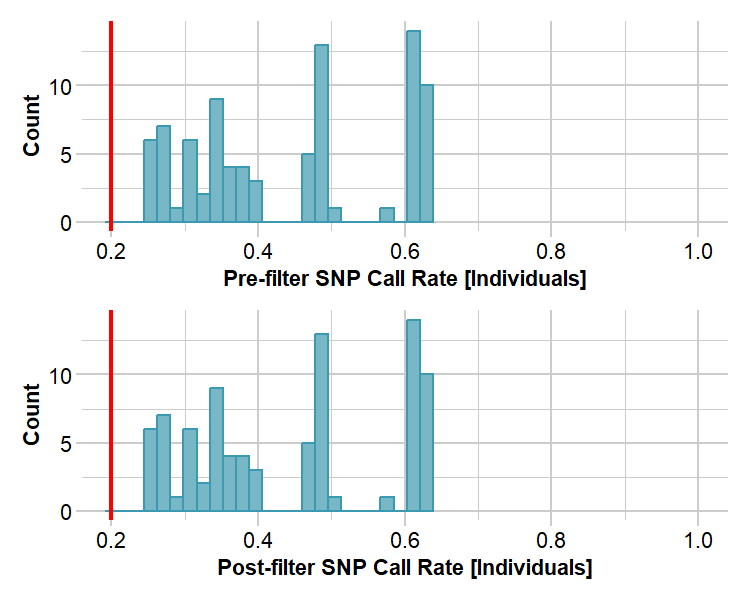**  **d)** |
| 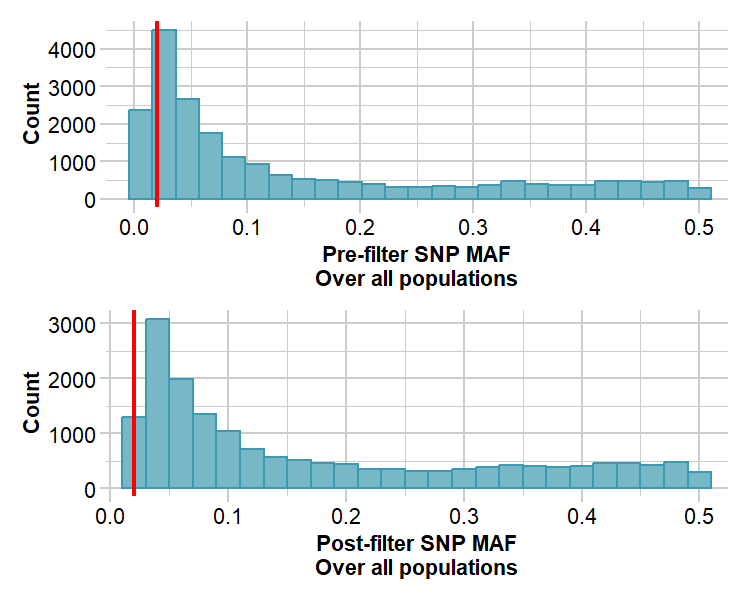  **e)** | **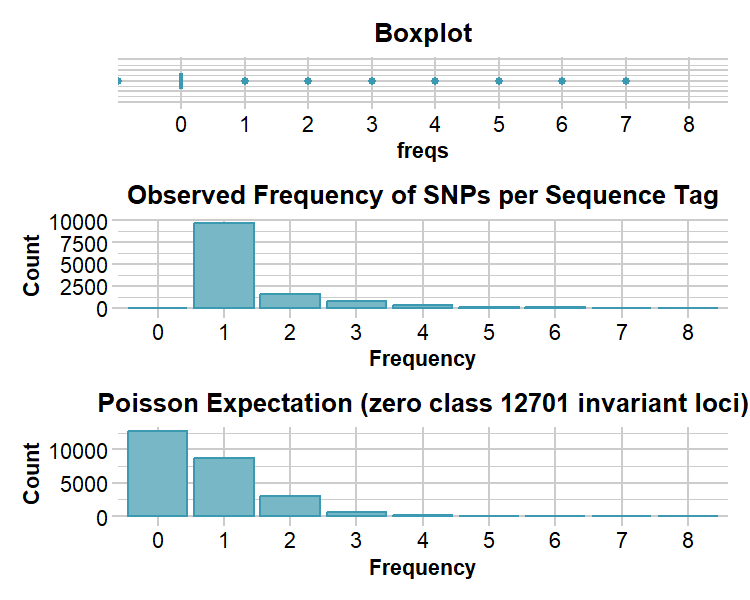**  **f)** |
| **Figure S2.** *Ctenotus* genus-level dataset filtering results of: a) reproducibility (≥90%); b) read depth (10 ≥ ≤50); c) call rate by loci (≥0.60); d) call rate by individual (≥0.20); e) minor allele frequency (≥0.02) and f) the report summary of indicating secondaries (SNPs that share a sequence tag and are likely linked) that were removed from analyses. Red bars represent the filter thresholds. | |

| **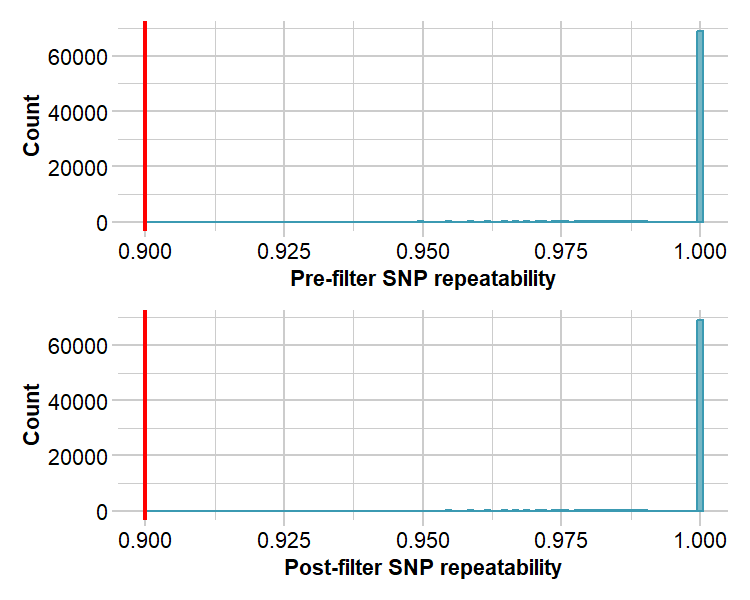**  **a)** | **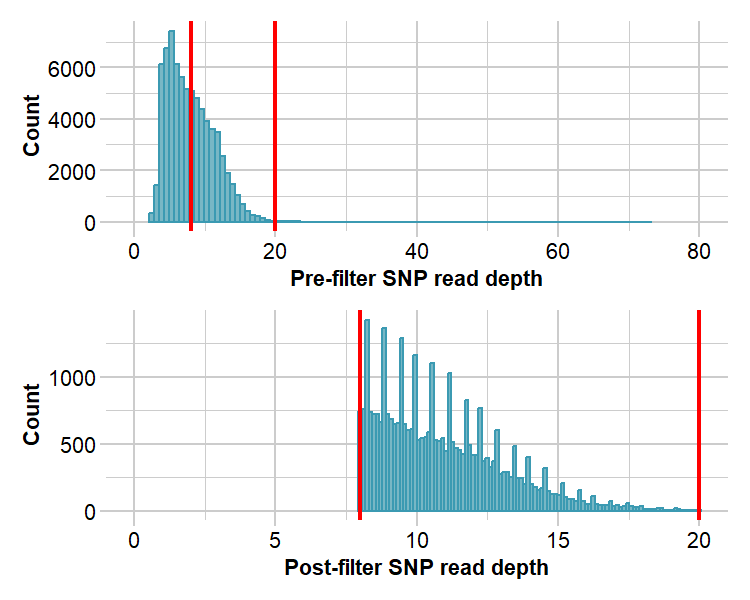**  **b)** |
| --- | --- |
| **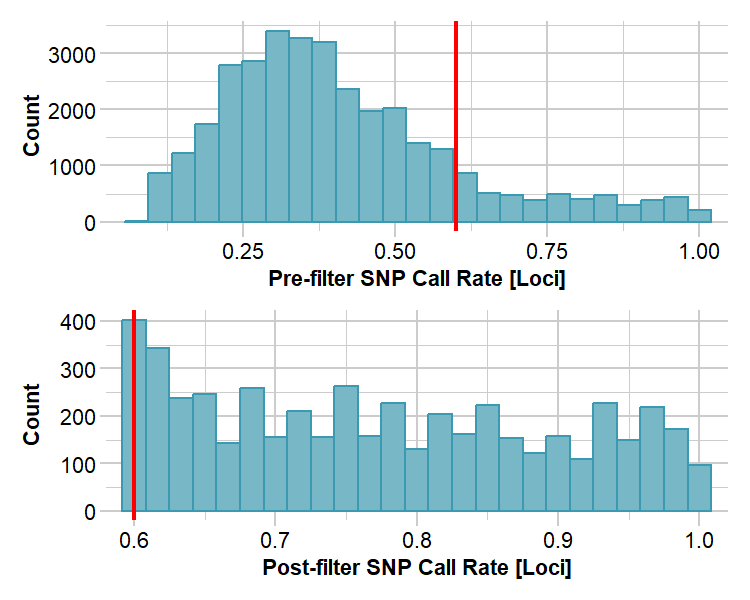**  **c)** | **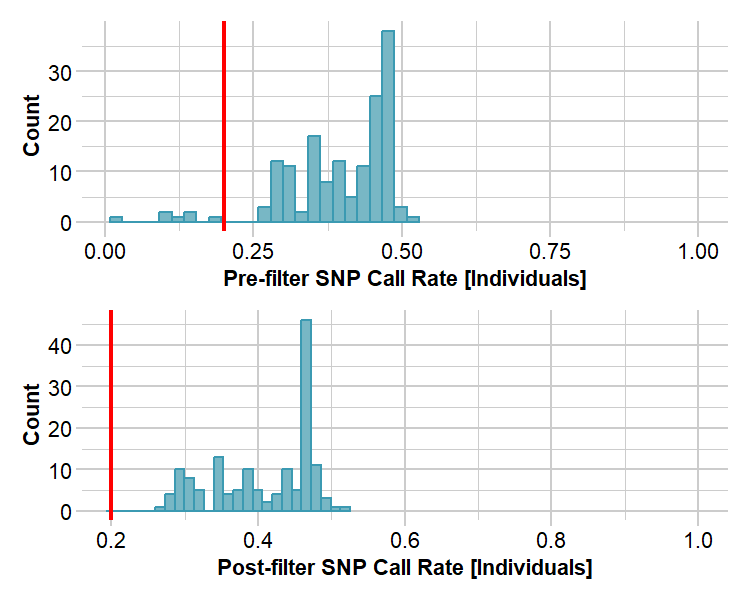**  **d)** |
| 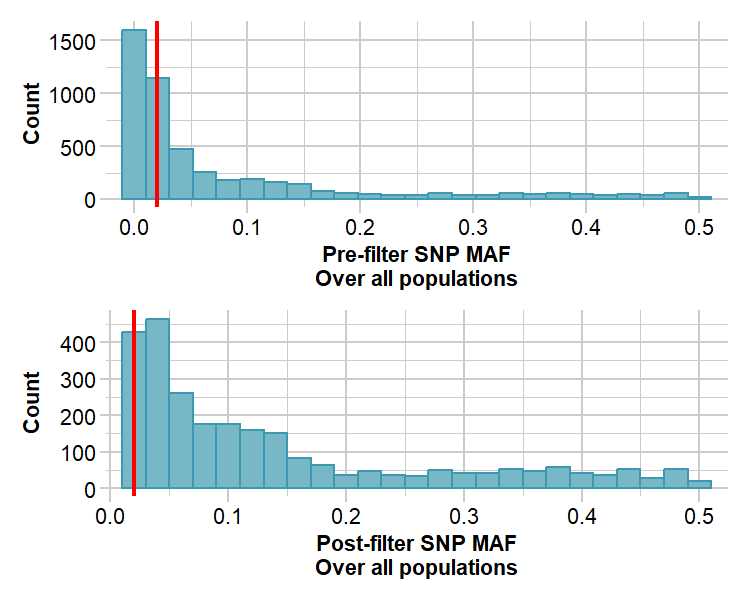  **e)** | **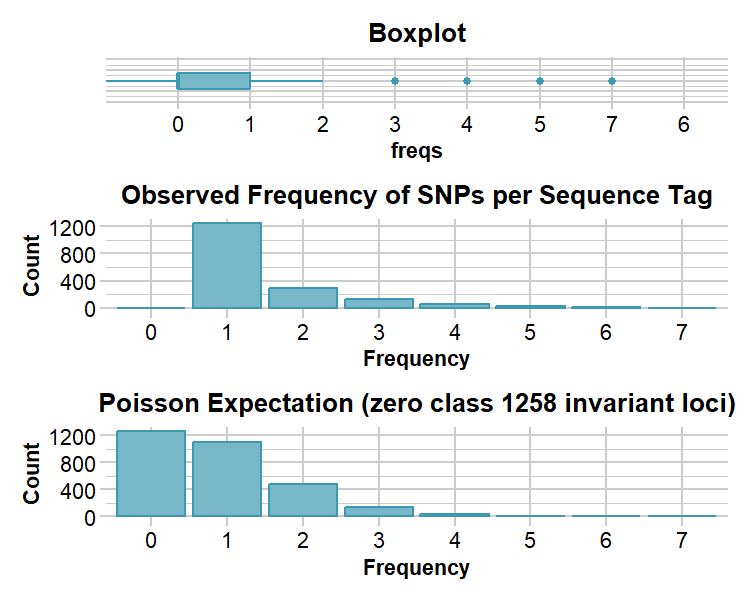**  **f)** |
| **Figure S3.** *Diporiphora* genus-level dataset filtering results of: a) reproducibility (≥90%); b) read depth (8 ≥ ≤20); c) call rate by loci (≥0.60); d) call rate by individual (≥0.20); e) minor allele frequency (≥0.02) and f) the report summary of indicating secondaries (SNPs that share a sequence tag and are likely linked) that were removed from analyses. Red bars represent the filter thresholds. | |

| 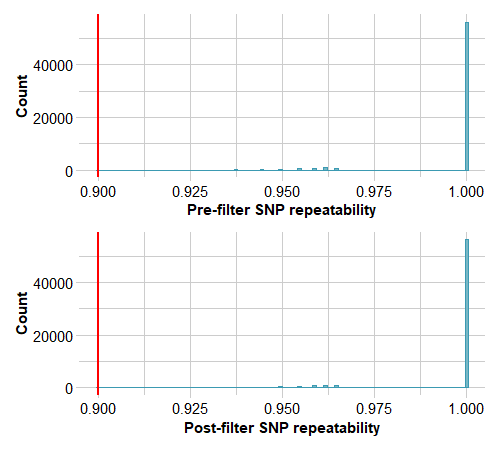  **a)** | **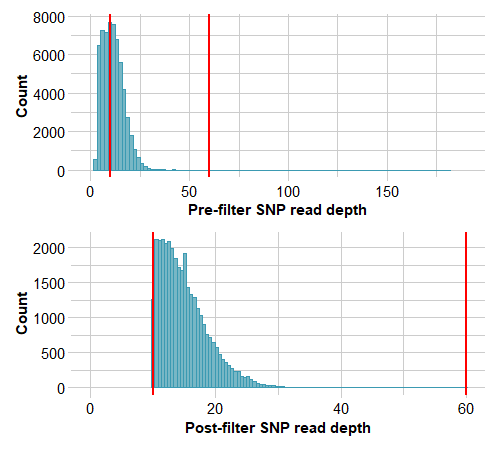**  **b)** |
| --- | --- |
| 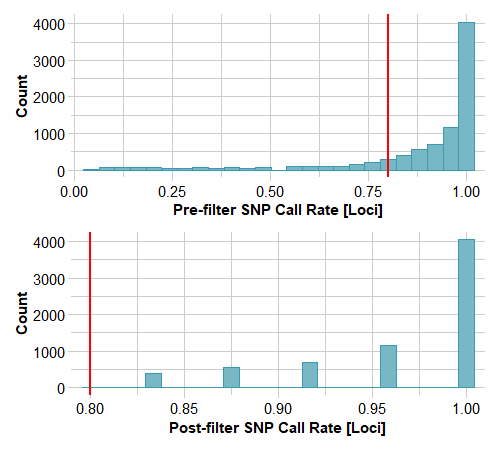  **c)** | 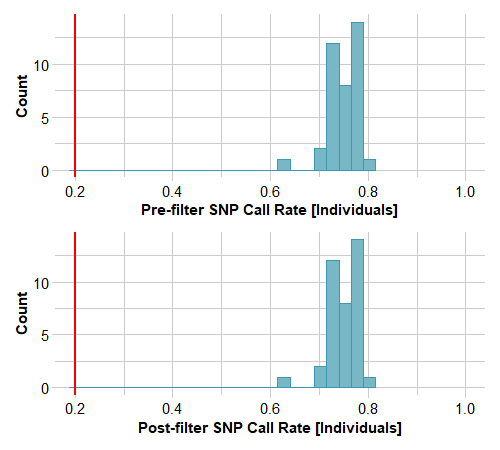  **d)** |
| 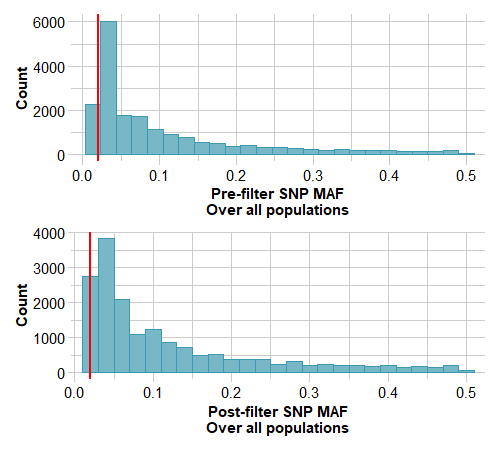  **e)** | **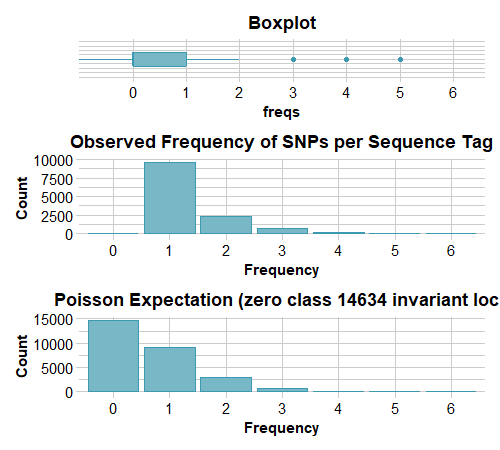**  **f)** |
| **Figure S4.** *C. amax* (ETE) dataset filtering results of: a) reproducibility (≥90%); b) read depth (10 ≥ ≤60); c) call rate by loci (≥0.75); d) call rate by individual (≥0.20); e) minor allele frequency (≥0.02) and f) the report summary of indicating secondaries (SNPs that share a sequence tag and are likely linked) that were removed from analyses. Red bars represent the filter thresholds. | |

| **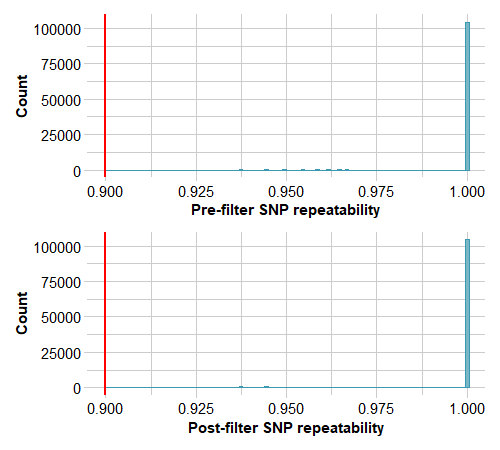**  **a)** | **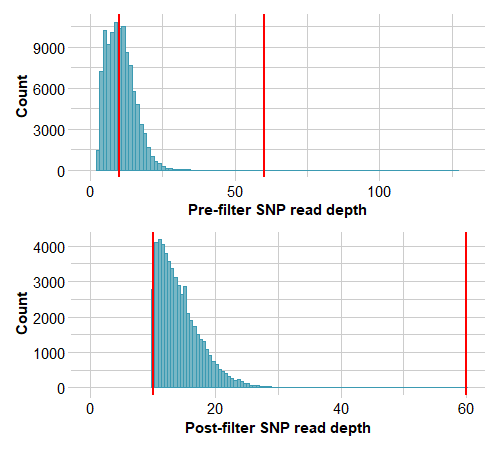**  **b)** |
| --- | --- |
| **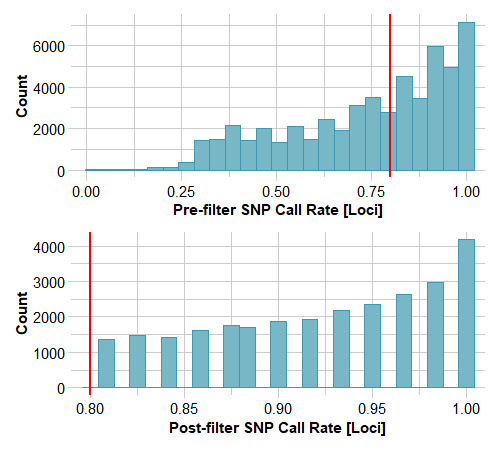**  **c)** | **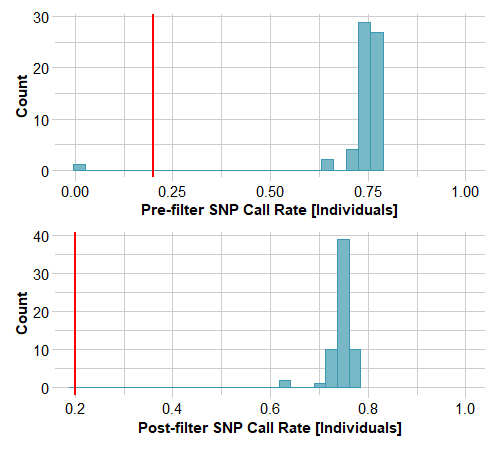**  **d)** |
| **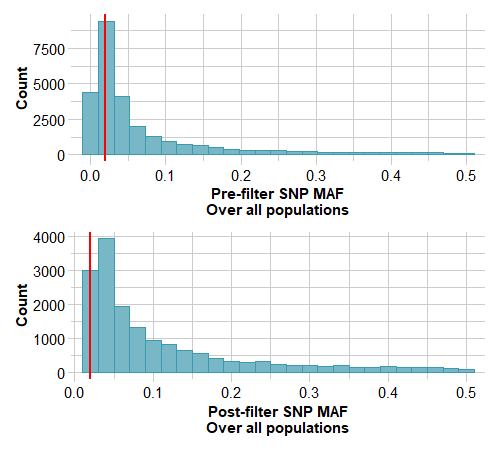**  **e)** | **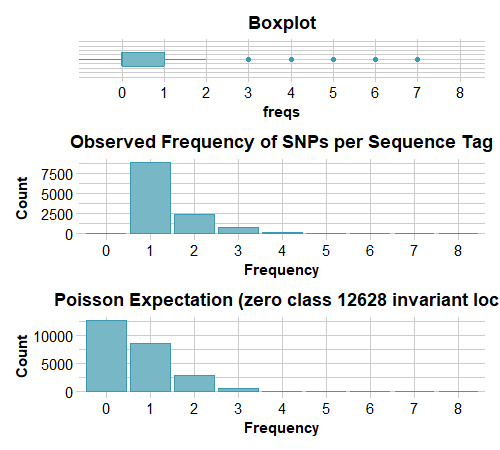**  **f)** |
| **Figure S5.** *C. munda* dataset filtering results of: a) reproducibility (≥90%); b) read depth (10≥ ≤60); c) call rate by loci (≥0.80); d) call rate by individual (≥0.20); e) minor allele frequency (≥0.02) and f) the report summary of indicating secondaries (SNPs that share a sequence tag and are likely linked) that were removed from analyses. Red bars represent the filter thresholds. | |

| \| **a)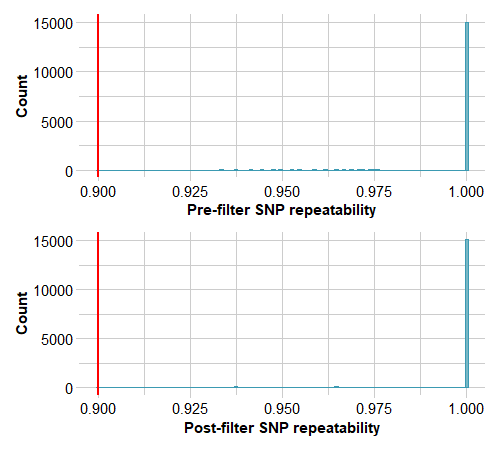** \| **b)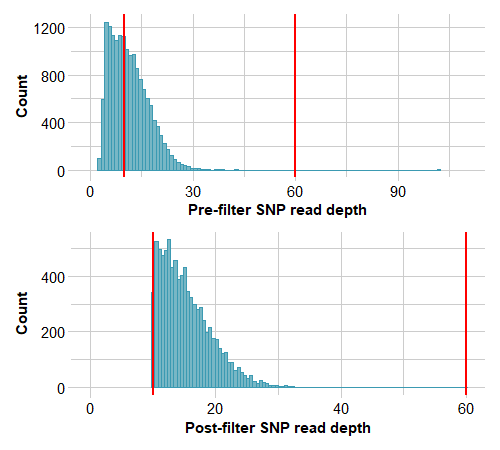** \| \| --- \| --- \| \| **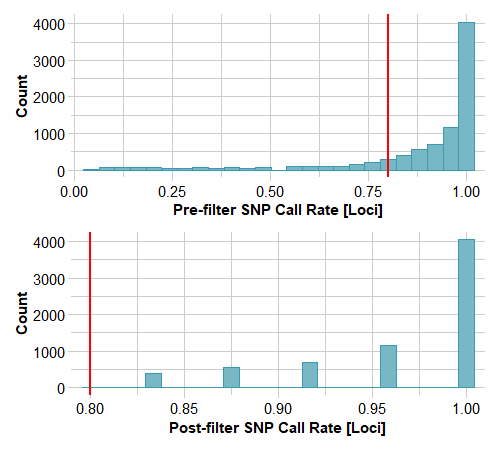**  **c)** \| **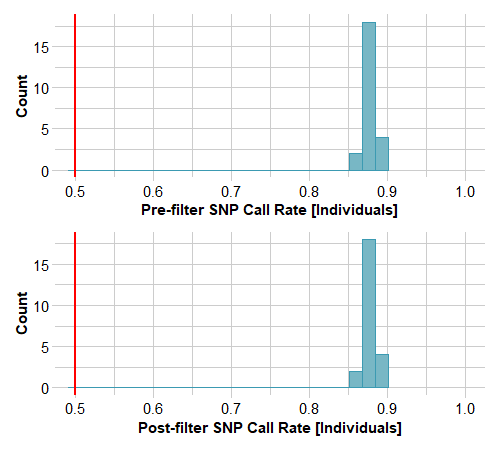**  **d)** \| \| 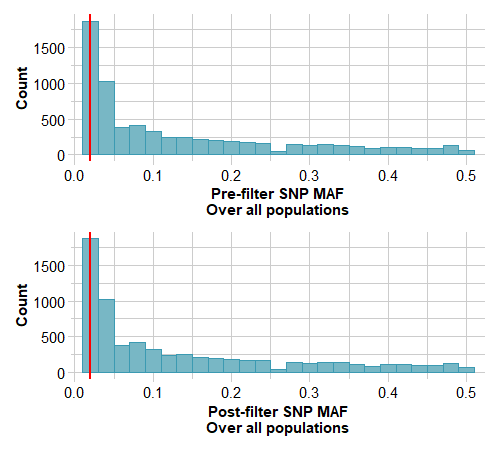  **e)** \| **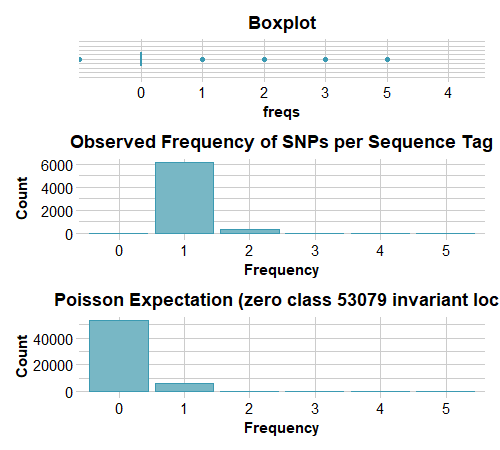**  **f)** \| \| **Figure S6.** *Ct. quirinus* dataset filtering results of: a) reproducibility (≥90%); b) read depth (8 ≥ ≤60); c) call rate by loci (≥0.80); d) call rate by individual (≥0.50); e) minor allele frequency (≥0.02) and f) the report summary of indicating secondaries (SNPs that share a sequence tag and are likely linked) that were removed from analyses. Red bars represent the filter thresholds. \| \| |  |
| --- | --- | --- | --- | --- | --- | --- | --- | --- | --- |

| **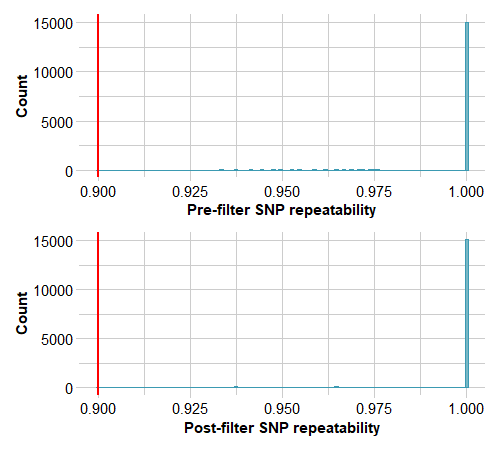**  **a)** | **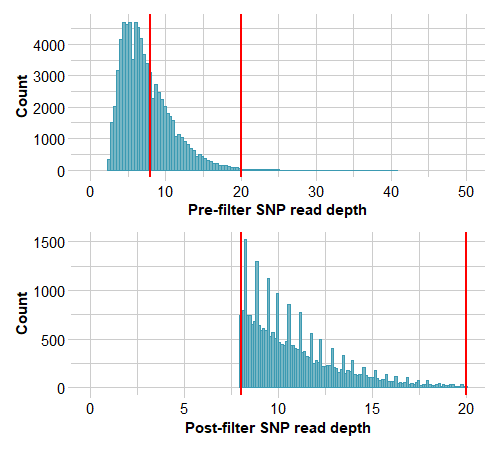**  **b)** |
| --- | --- |
| **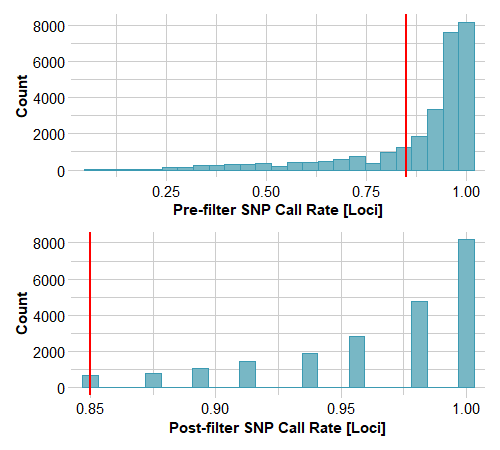**  **c)** | **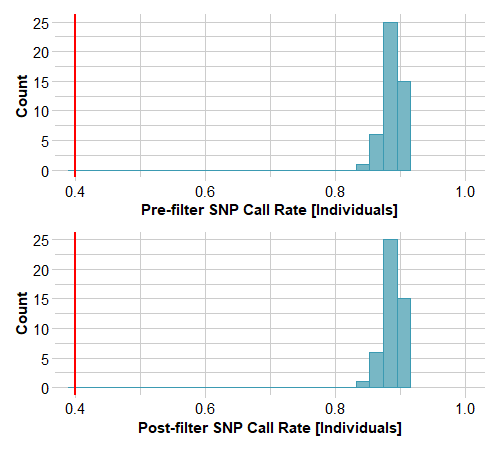**  **d)** |
| **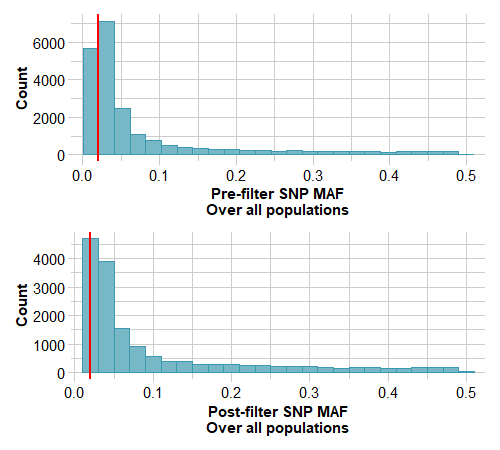**  **e)** | **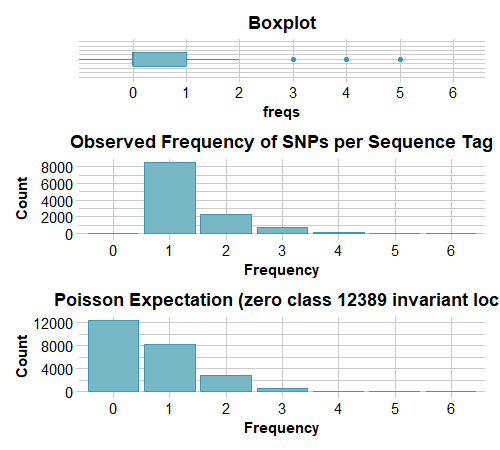**  **f)** |
| **Figure S7.** *D. bilineata* (ETE) dataset filtering results of: a) reproducibility (≥95%); b) read depth (8 ≥ ≤20); c) call rate by loci (≥0.80); d) call rate by individual (≥0.40); e) minor allele frequency (≥0.02) and f) the report summary of indicating secondaries (SNPs that share a sequence tag and are likely linked) that were removed from analyses. Red bars represent the filter thresholds. | |
| **a)**  **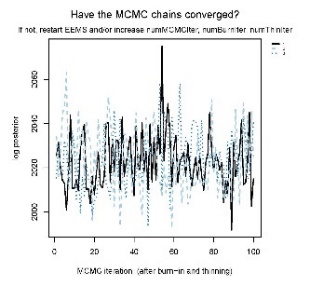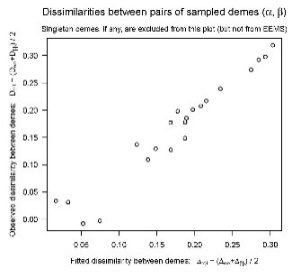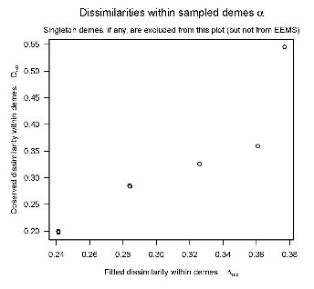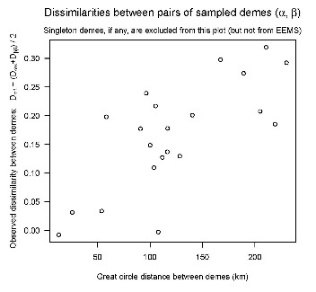** | |
| **b)**  **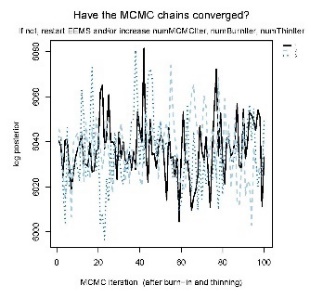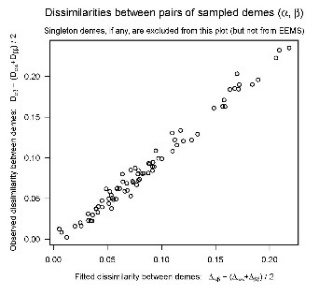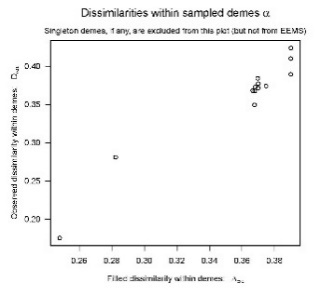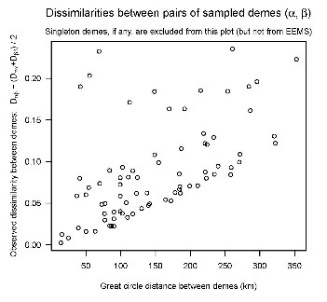** | |
| **c)**  **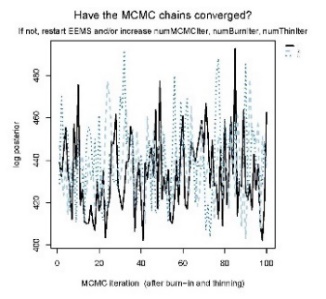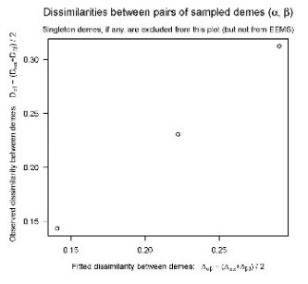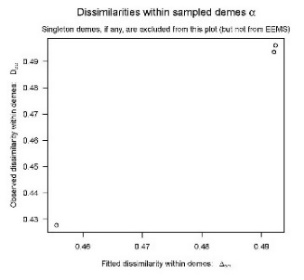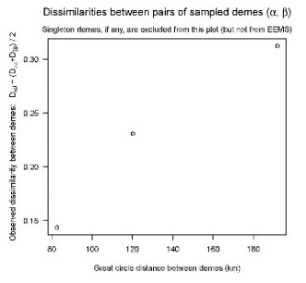** | |
| **d)**  **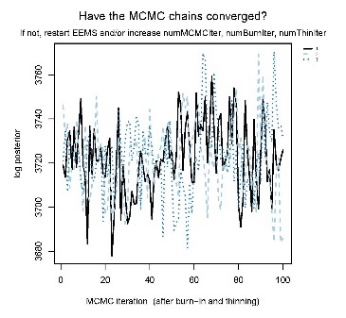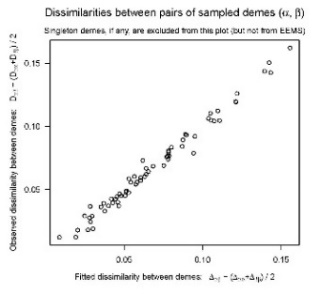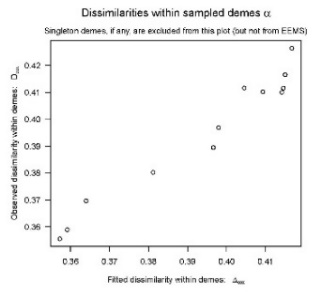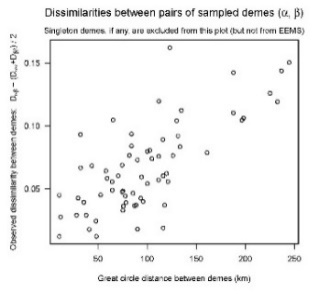** | |
| **Fig S8.** EEMS (estimated effective migration surfaces) diagnostic plots for a) *C. amax,* b) *C. munda,* c) *Ct. quirinus* and d) *D. bilineata.* | |

#
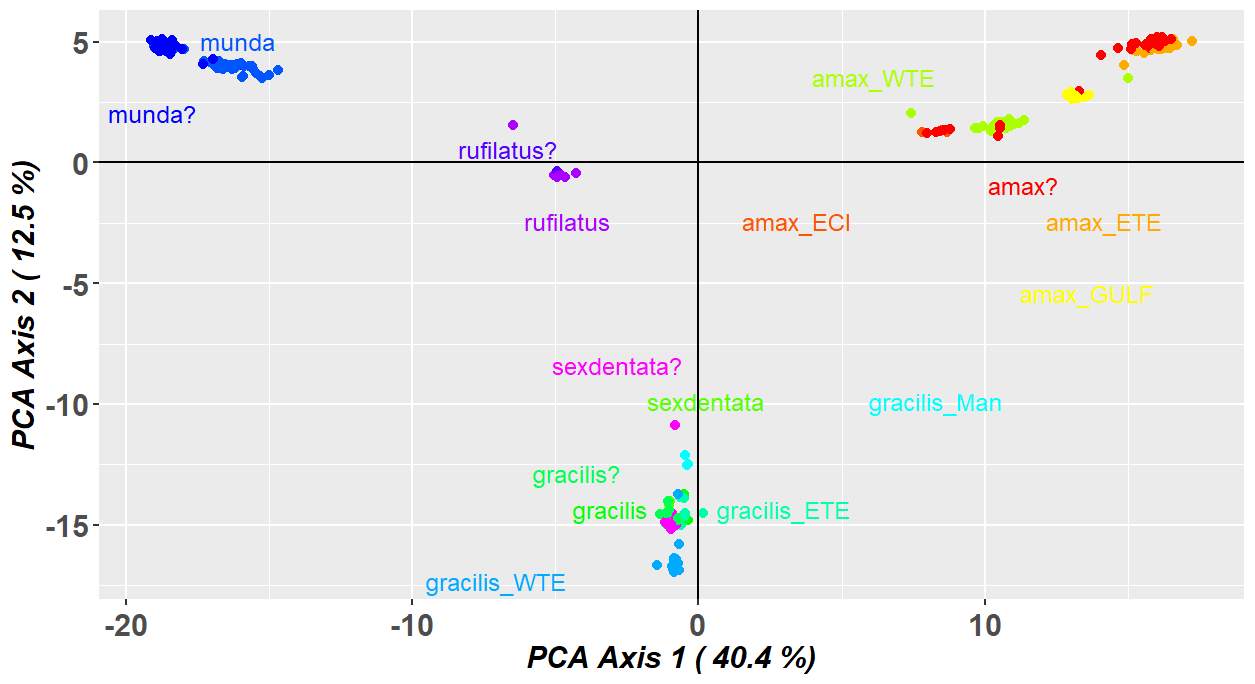


**Figure S9.** PCA of the *Carlia* genus SNP data (n=336). Species names followed by question marks indicate samples from this study that were tentatively allocated to species groups using PCA and confirmed with phylogenetic analyses (*amax?, gracilis?, munda?, rufilatus?, sexdentata?)*. Reference samples from previous analyses do not have question marks and display lineage allocations where relevant (WTE =Western Top End, ETE = Eastern Top End, Man = Maningrida, ECI = English Company Islands, GULF= Gulf of Carpentaria).


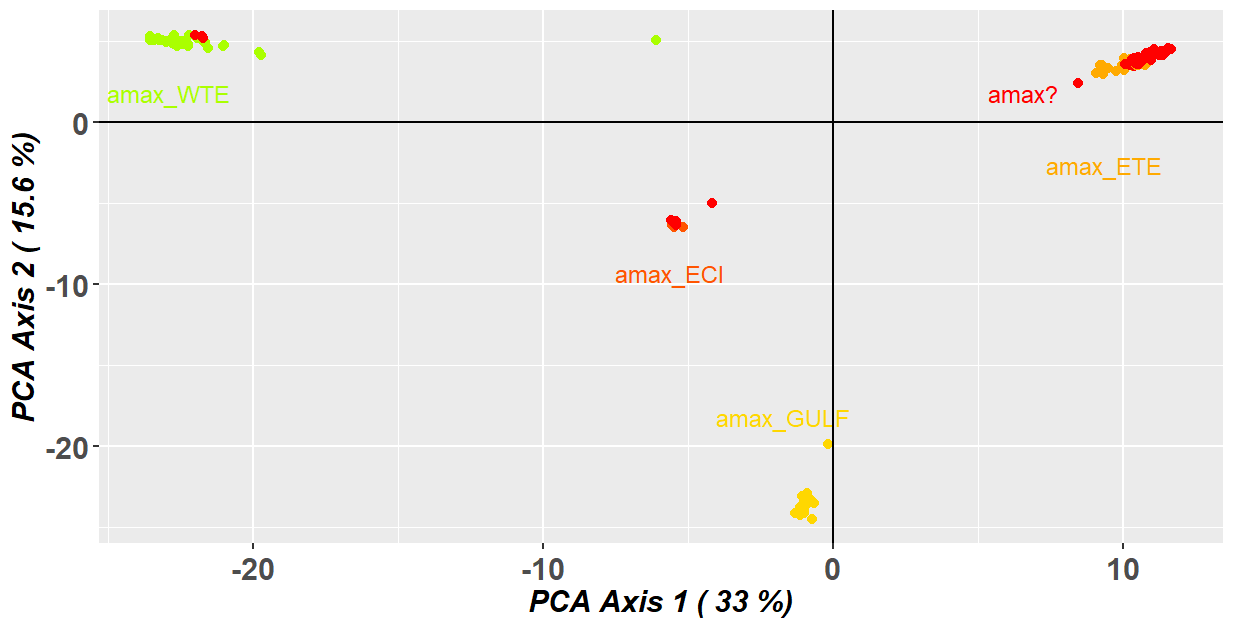


**Figure S10.** PCA of *Carlia amax* SNP data indicating samples from this study (‘amax?), with’ reference samples genetically allocated to lineages (WTE =Western Top End, ETE= Eastern Top End, ECI = English Company Islands, GULF = Gulf of Carpentaria).


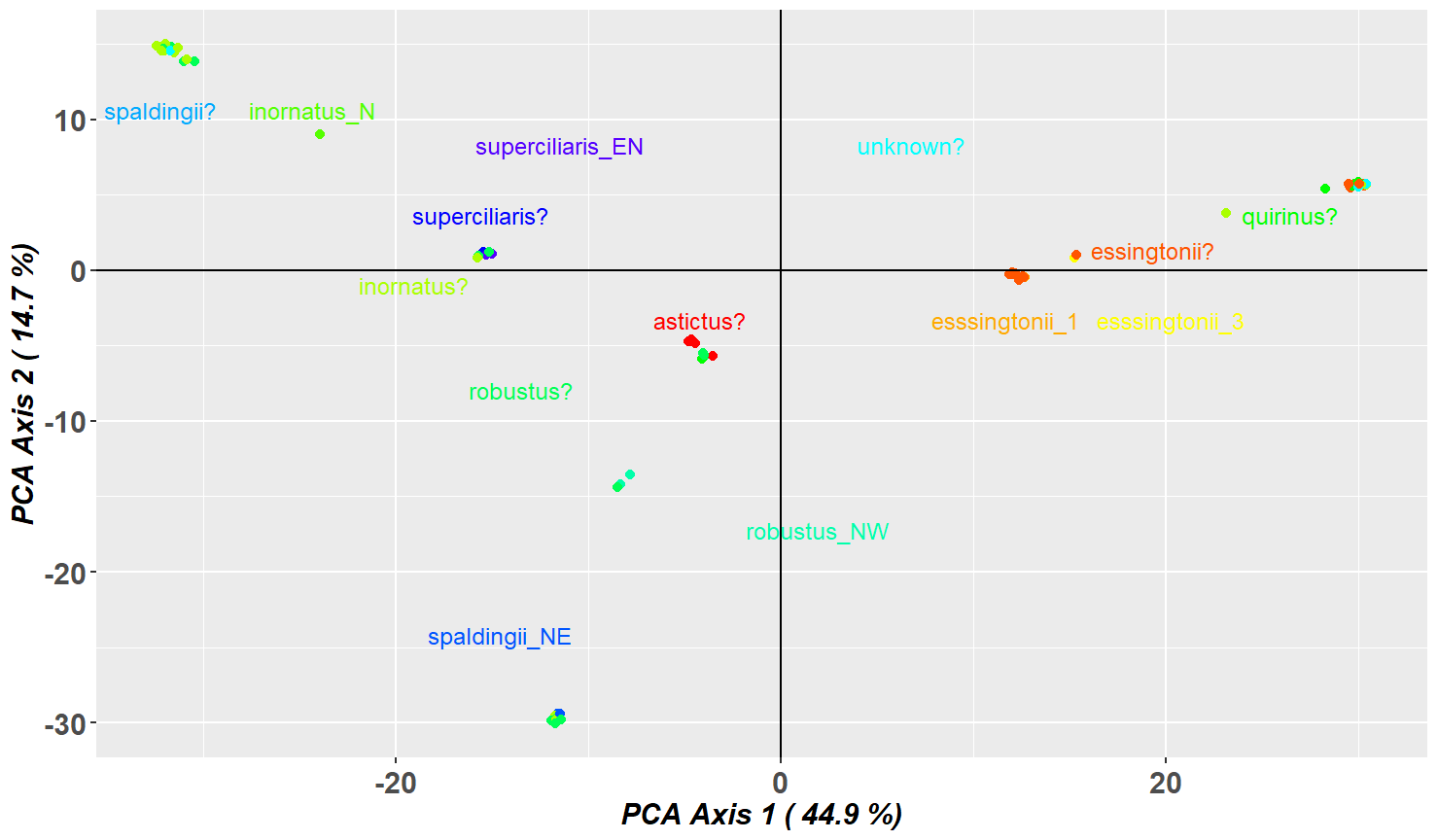


**Figure S11.** PCA of *Ctenotus* genus SNP data. Species names followed by question marks indicate samples from this study that were tentatively allocated to species groups using this PCA and confirmed with phylogenetic analyses. Reference samples from previous analyses do not have question marks and display lineage allocations where relevant based on geographic distribution (N = North, NE= North East, NW = North West, EN =East North), except for *Ct. essingtonii* which has numbered lineages (1, 2 and 3) which can have overlapping geographic distributions.


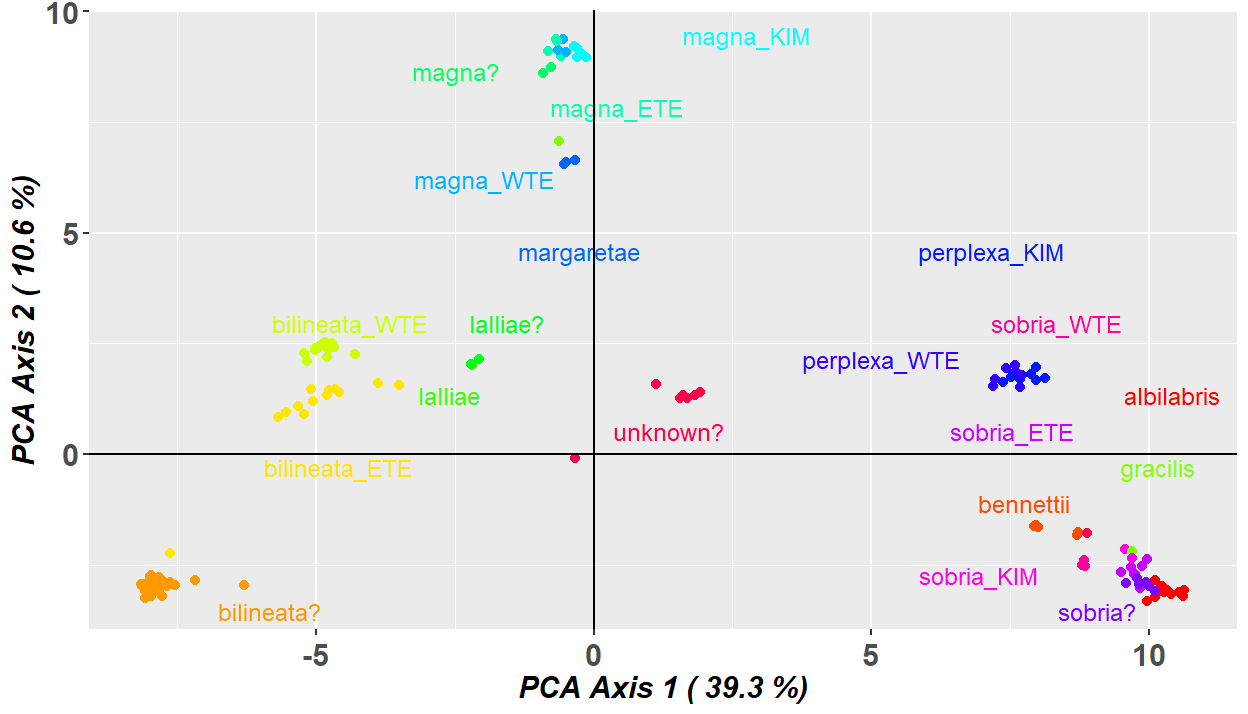


**Figure S12.** PCA of *Diporiphora* genus SNP data. Species names followed by question marks indicate samples from this study that were tentatively allocated to species groups using this PCA and confirmed with phylogenetic analyses (bilineata?, lalliae?, sobria?, magna?, unknown?). Reference samples from previous analyses do not have question marks and display lineage allocations where relevant based on their geographic distribution (WTE =Western Top End, ETE= Eastern Top End, KIM =Kimberely region).


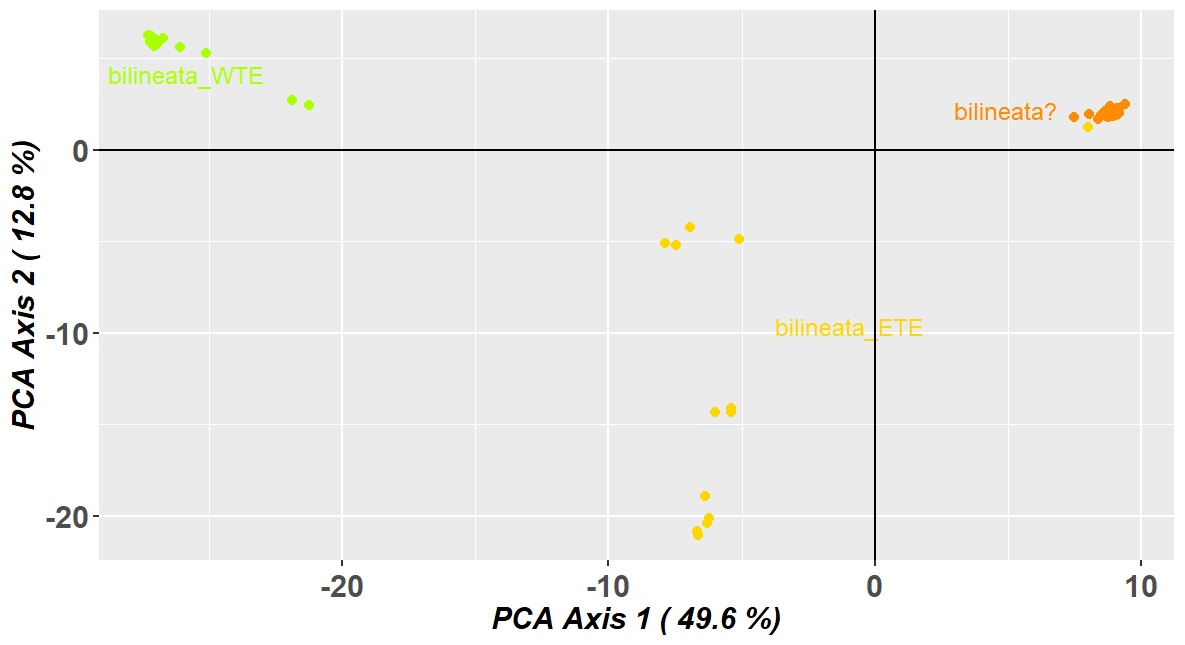


**Figure S13.** PCA of *Diporiphora bilineata* SNP data. Label ‘bilineata?’ indicate samples from this study. Reference samples from previous analyses do not have question marks and display lineage allocations where relevant based on their geographic distribution (WTE =Western Top End, ETE= Eastern Top End).

**e**

**d**

**b**

**c**

**a**


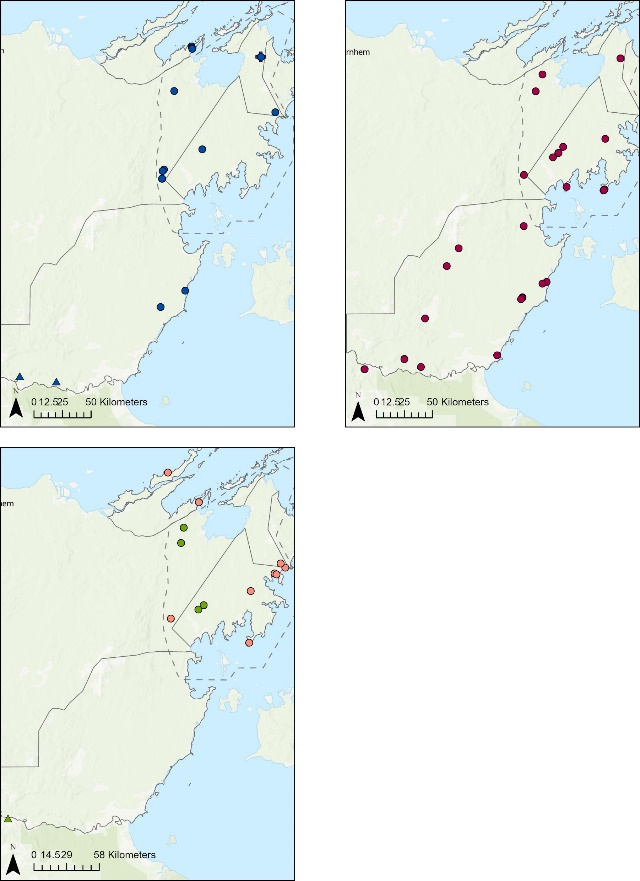

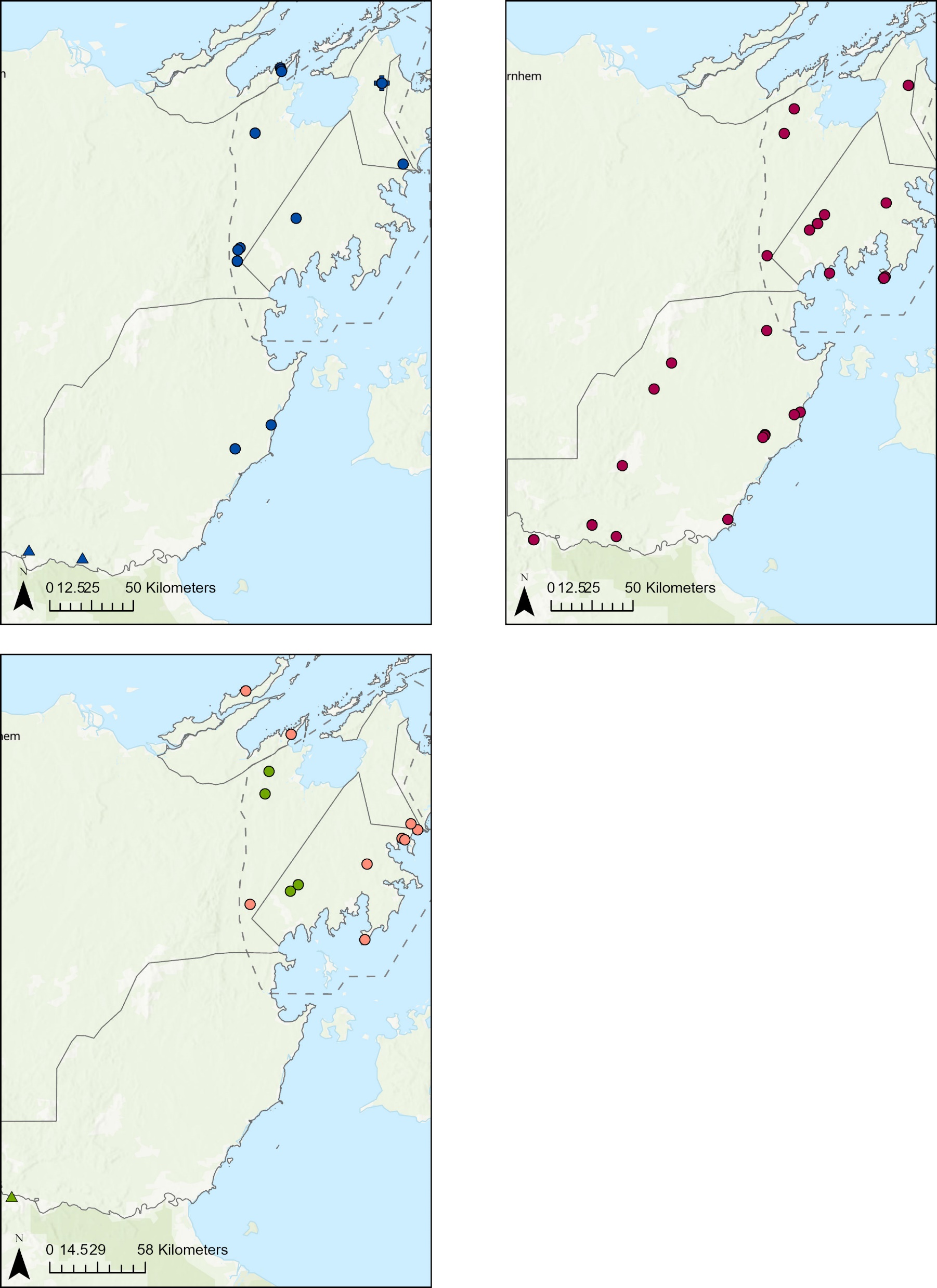


*C. amax*

*C. munda*


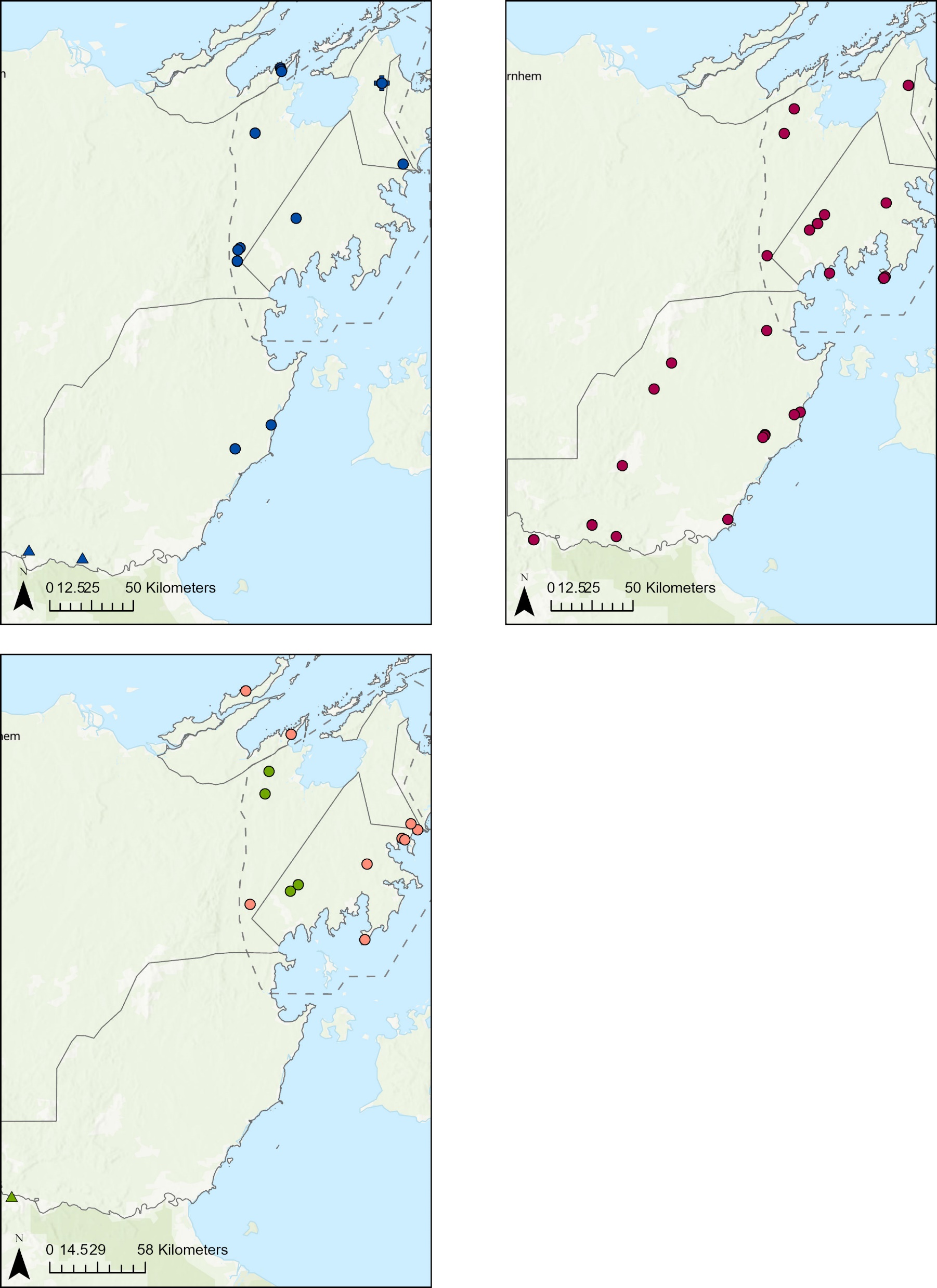


*C. gracilis*

*C. sexdentata*


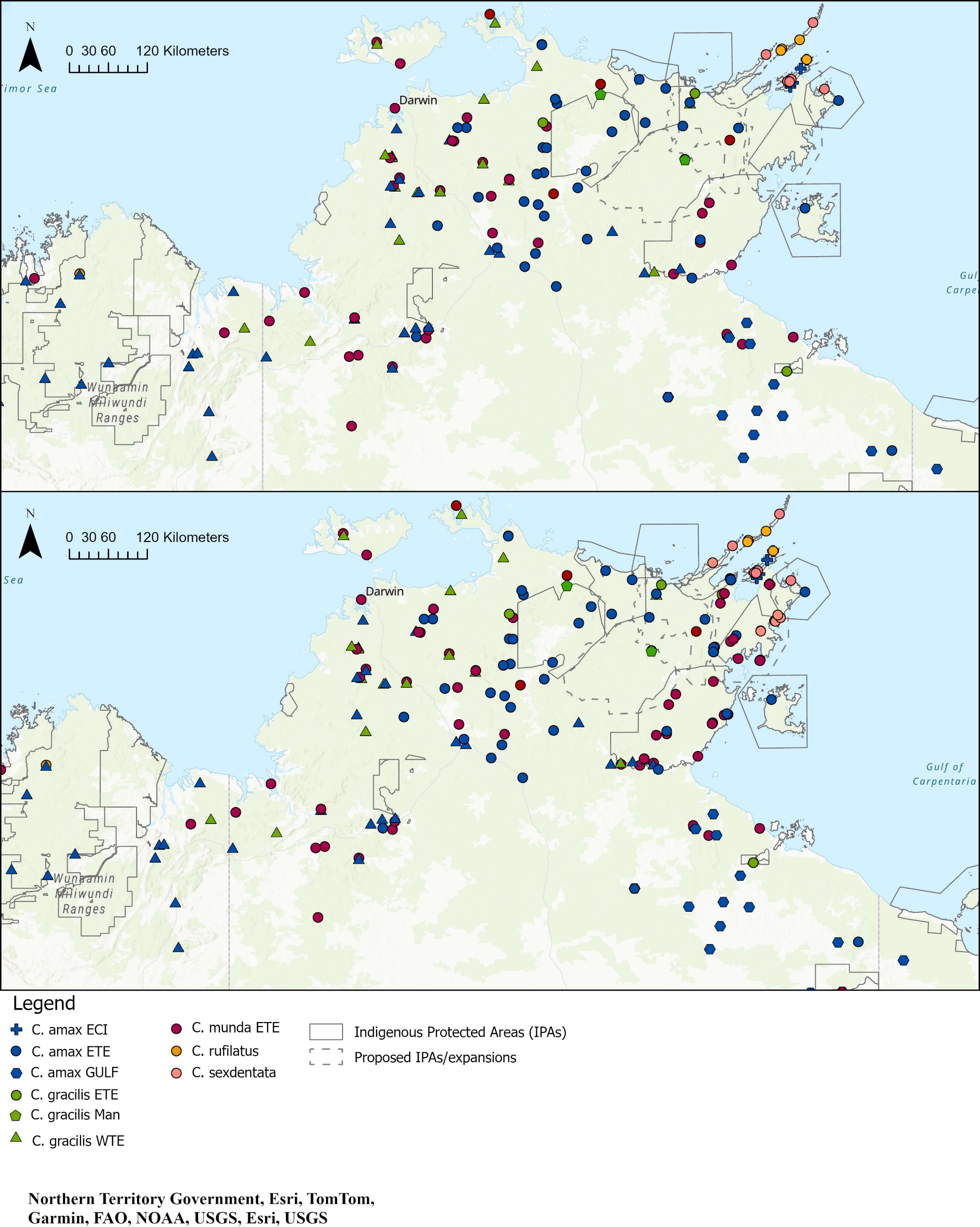


**Figure S14**. Map of *Carlia* **a)** reference samples **b)** reference and study samples from across the Top End, and individual species maps of **c)** *C. amax,* **d)** *C. munda* and **e)** *C. gracilis* and *C. sexdentata* collected from north East Arnhem Land.


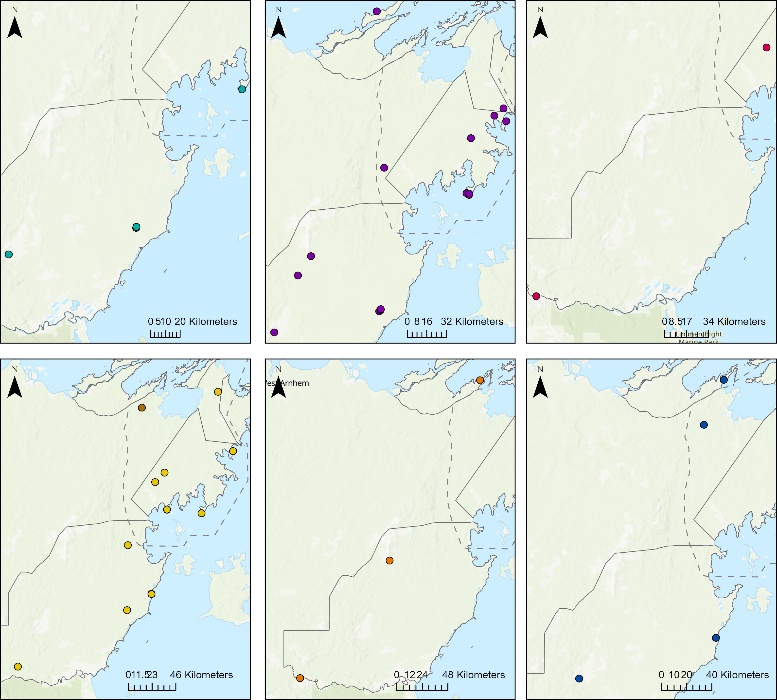


*Ct. quirinus*


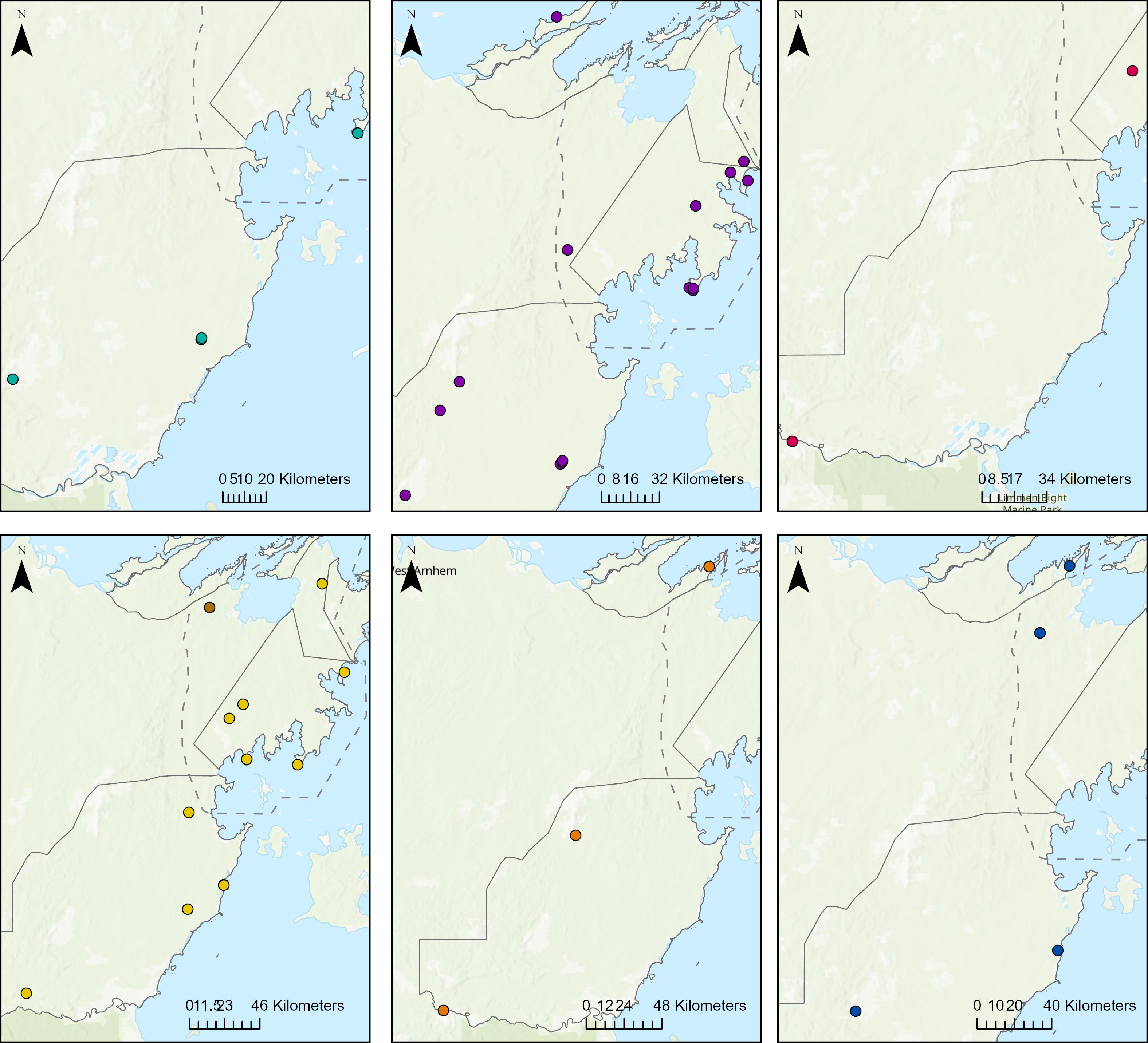


*Ct. spaldingi*


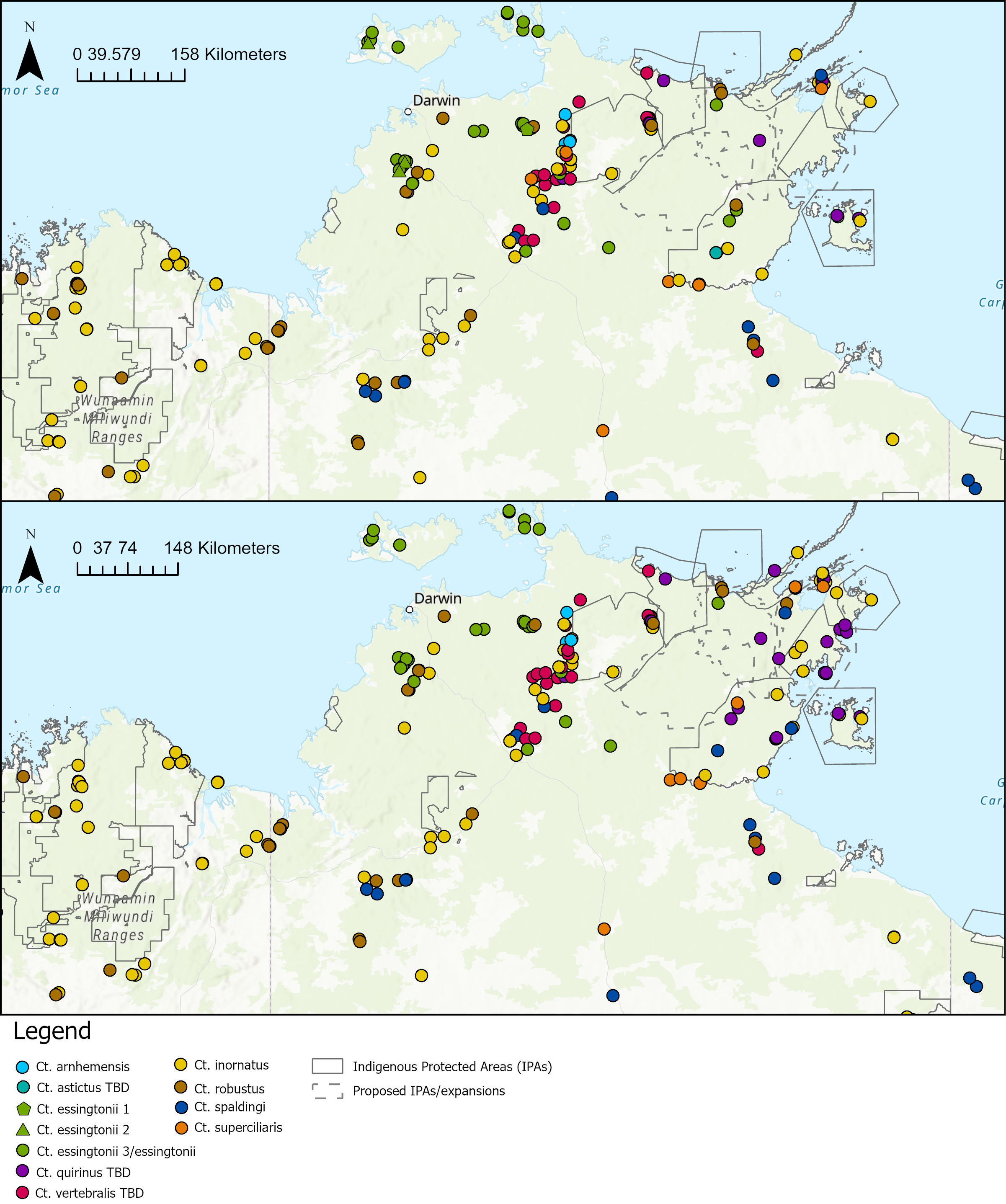

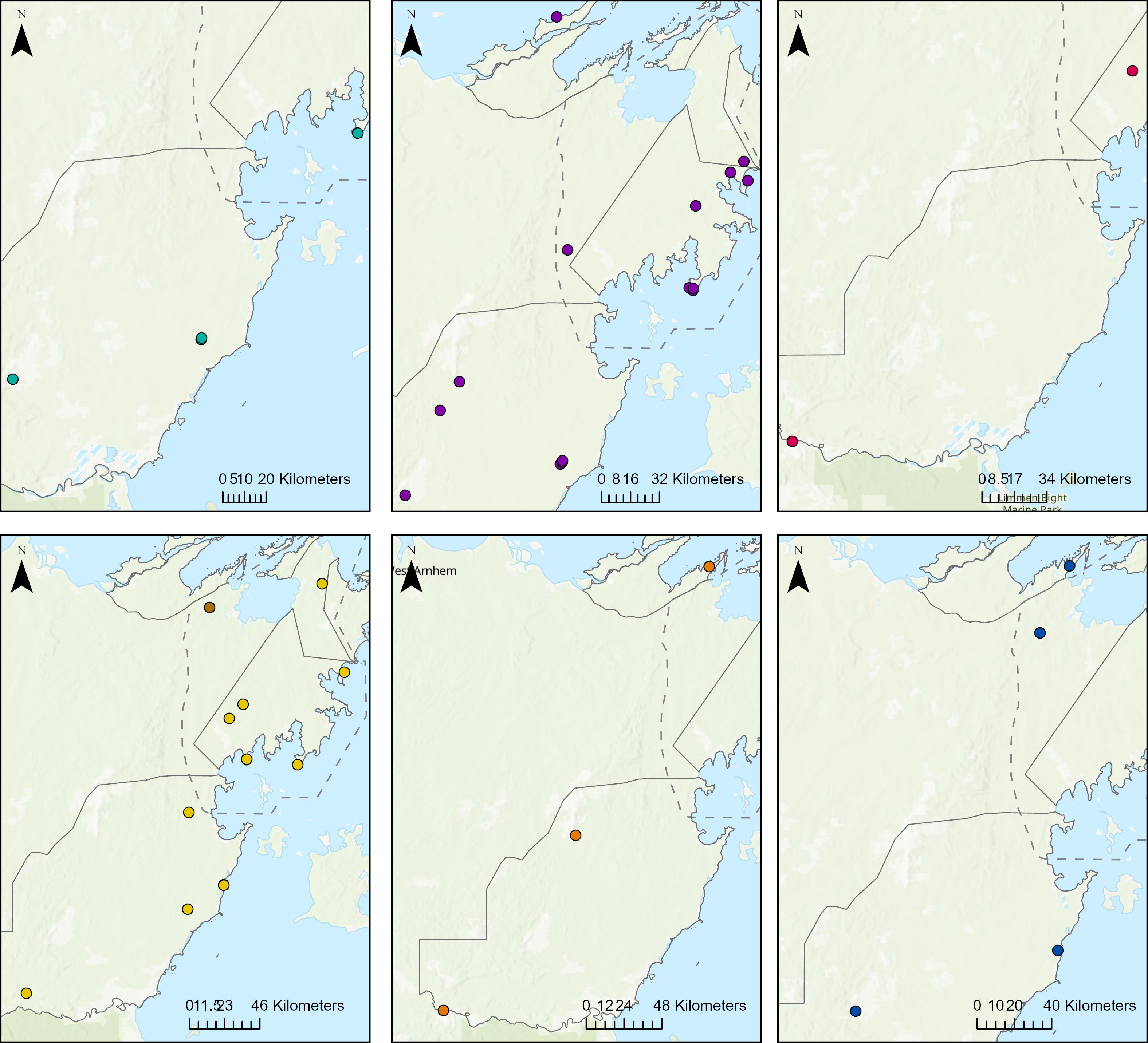


*Ct. astictus*


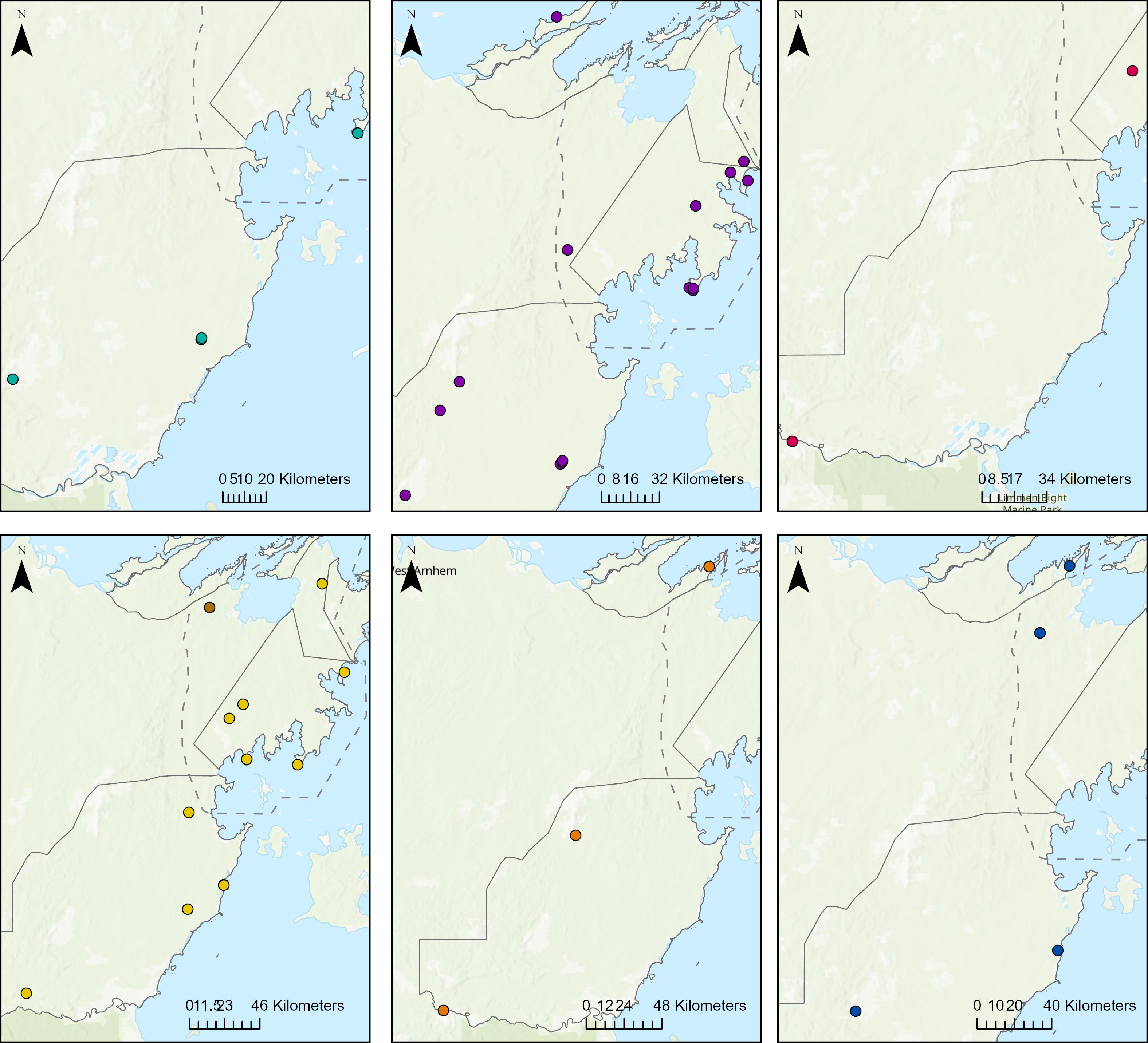


*Ct. vertebralis*


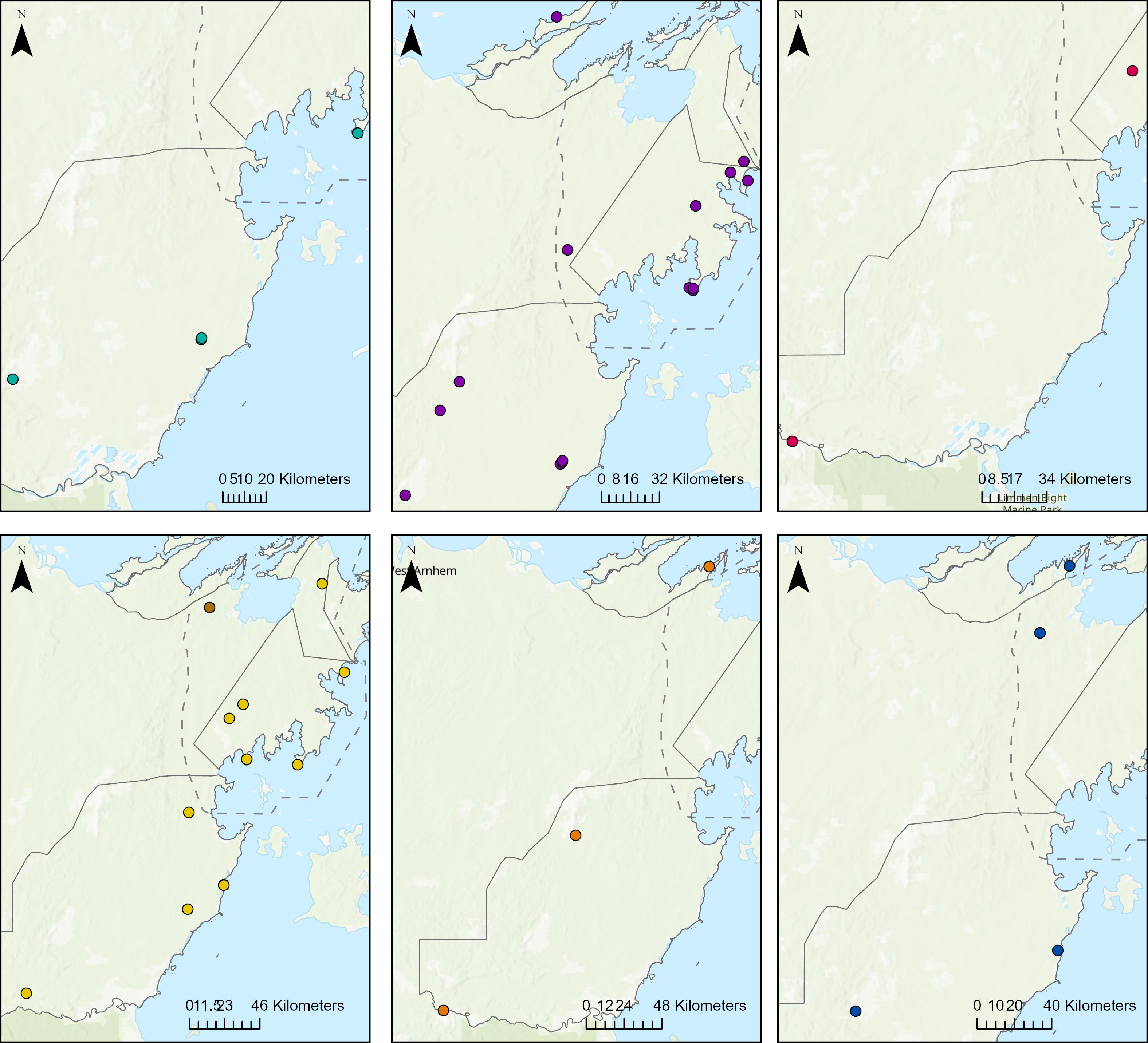


*Ct. inornatus*

*Ct. robustus*


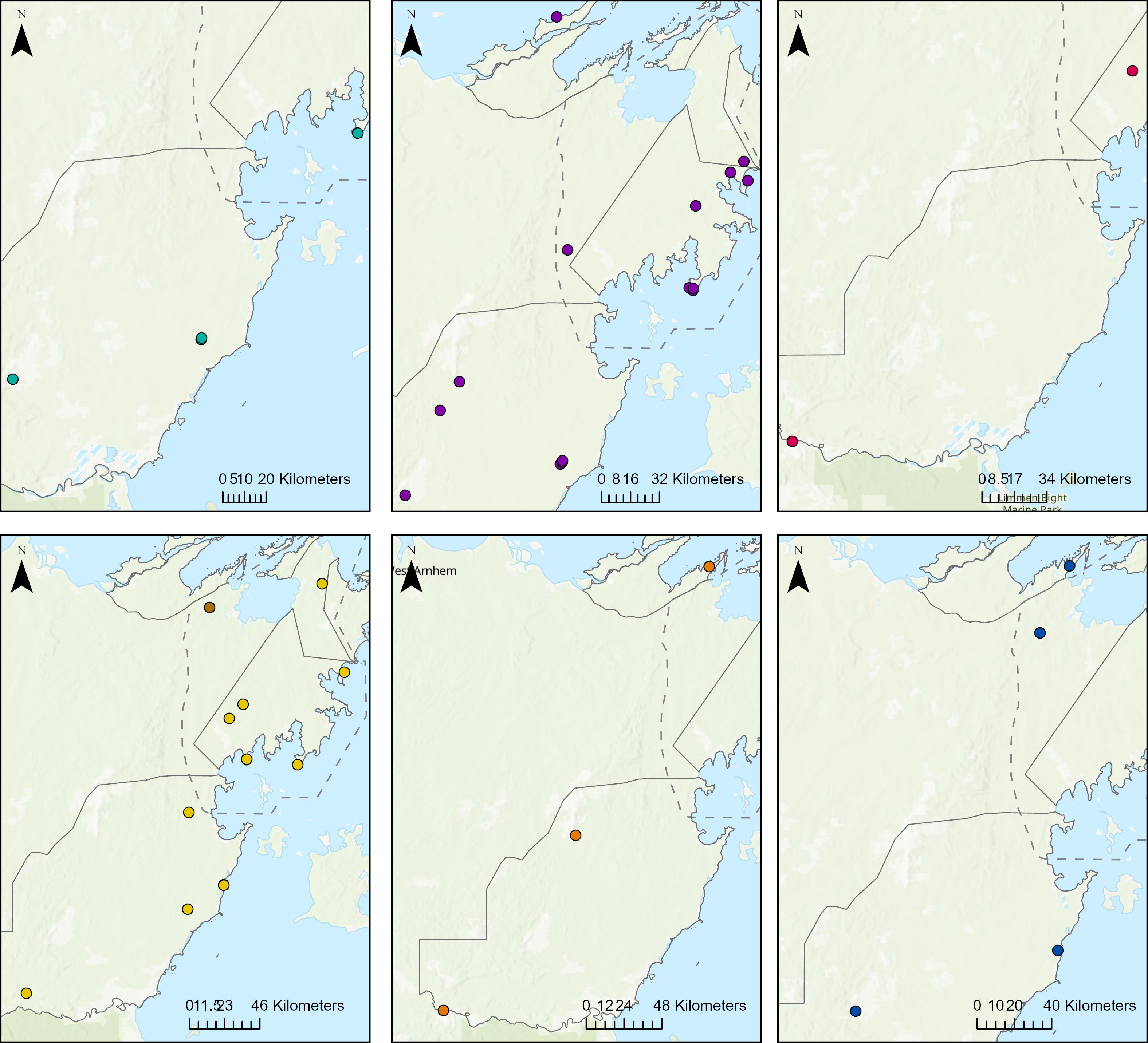


*Ct. superciliaris*

**a**

**b**

**c**

**d**

**e**

**f**

**g**

**h**

**Figure S15**. Map of *Ctenotus* **a)** reference samples and known *Ctenotus* samples from XX database **b)** reference and study samples from across the Top End, and individual species maps of **c)** *Ct. astictus,* **d)** *Ct. spaldingi,* **e)** *Ct. quirinus* and *Ct. superciliaris,* **f)** *Ct. vertebralis,* **g)** *Ct. inornatus* and *Ct. robustus* collected from north East Arnhem Land.


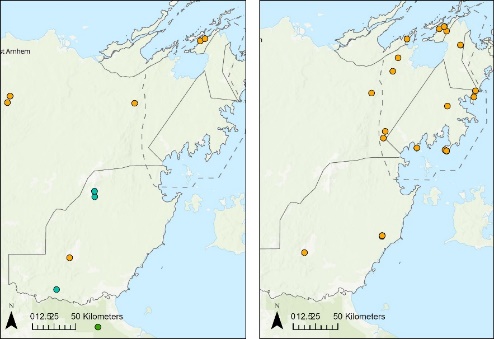

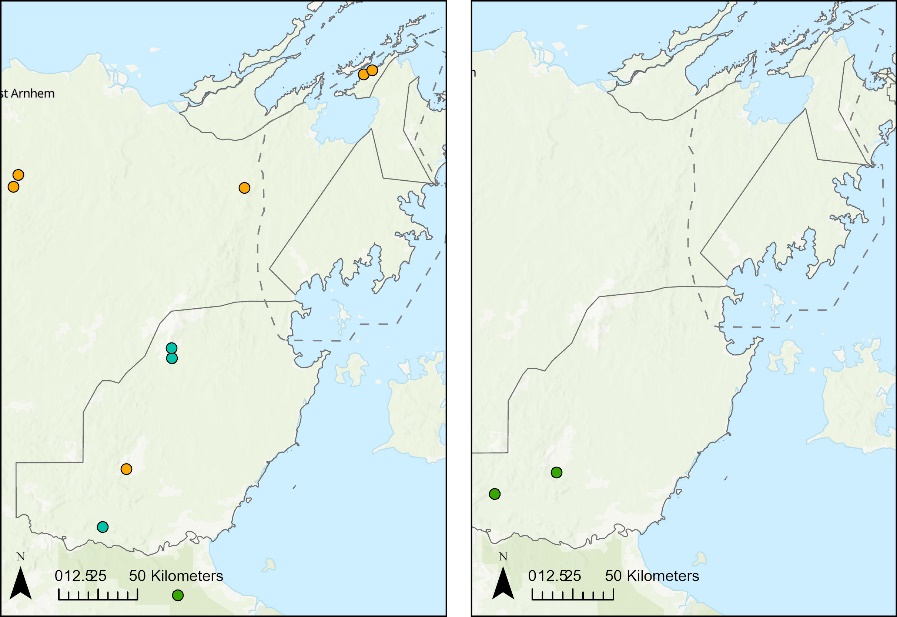

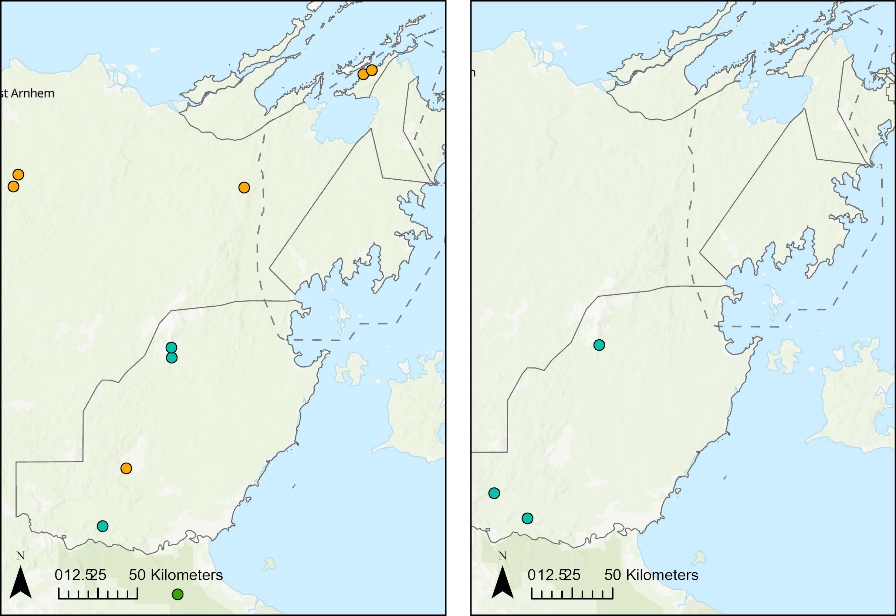

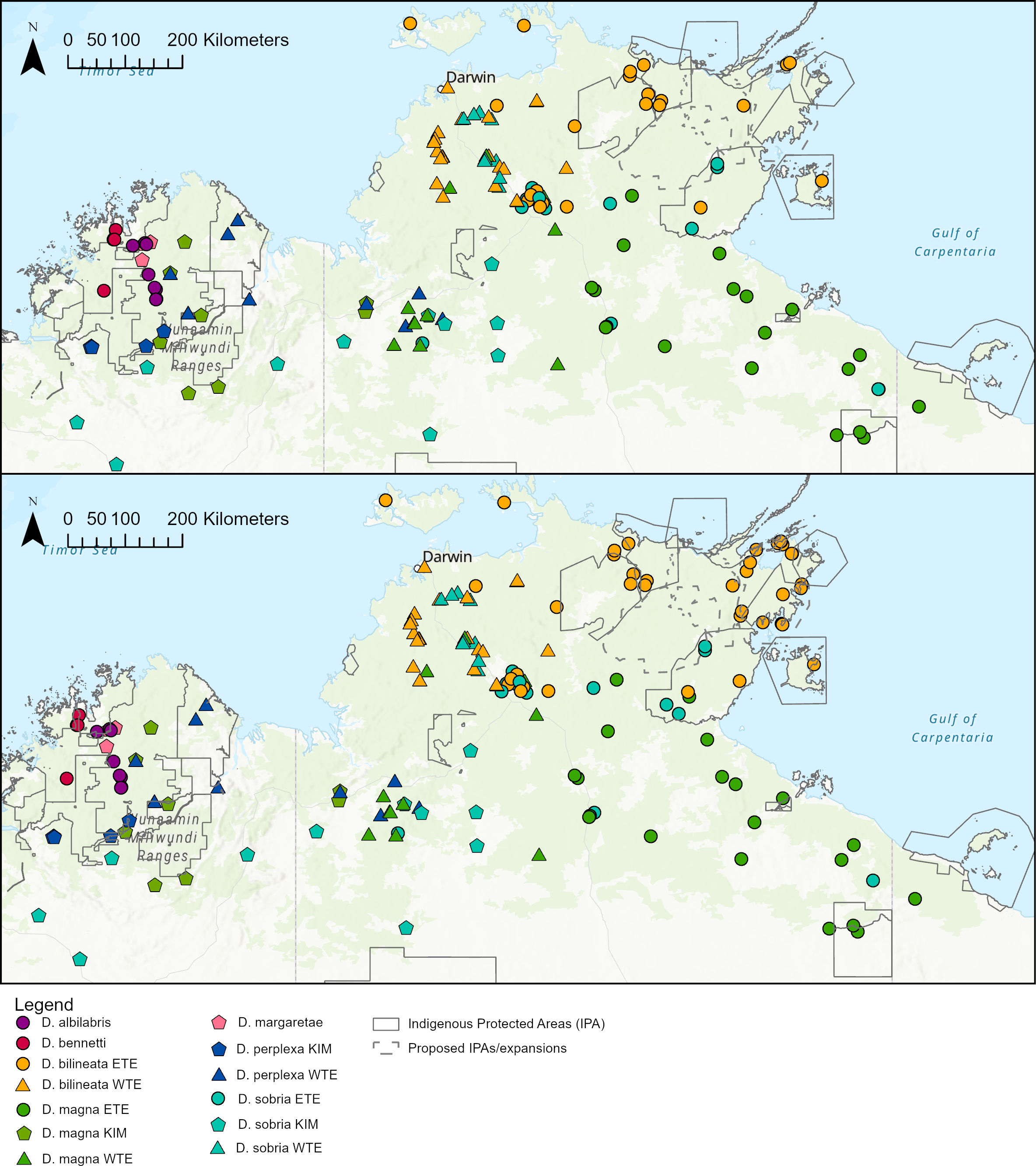


*D. bilineata*

*D. magna*

*D. sobria*

**a**

**b**

**c**

**d**

**e**

**Figure S16**. Map of *Diporiphora* **a)** reference samples **b)** reference and study samples from across the Top End, and individual species maps of **c)** *D. bilineata,* **d)** *D. magna* and **e)** *D. sobria* collected from north East Arnhem Land.


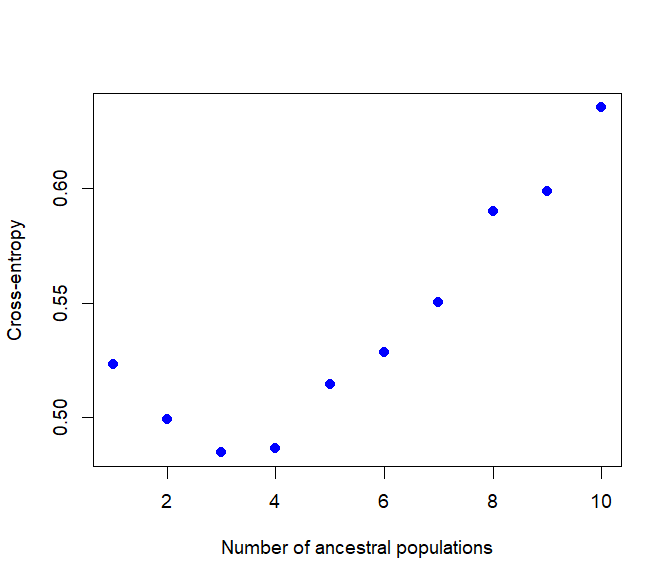

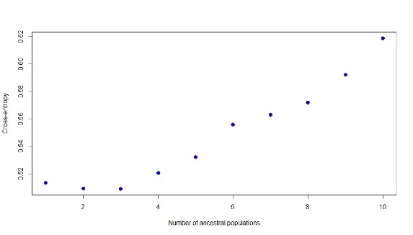

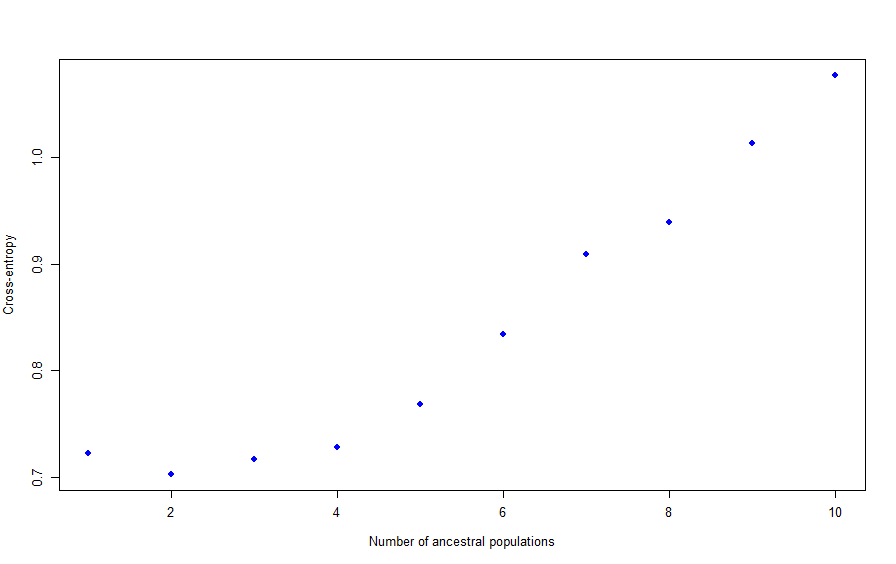

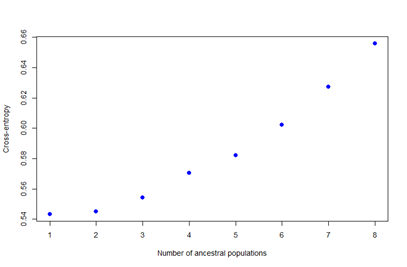

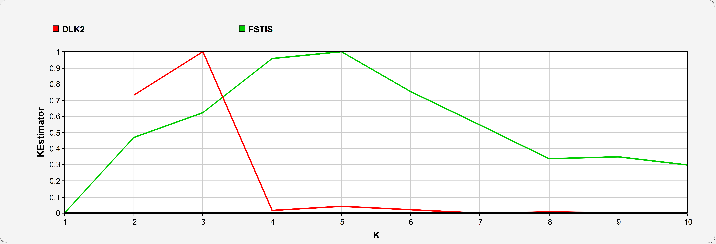

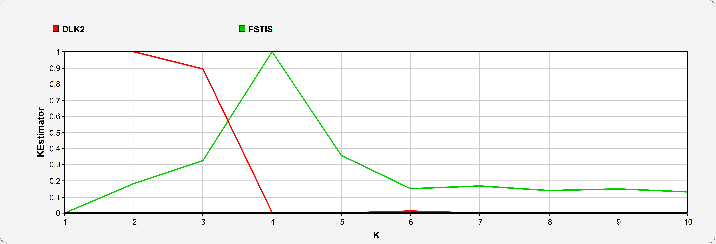

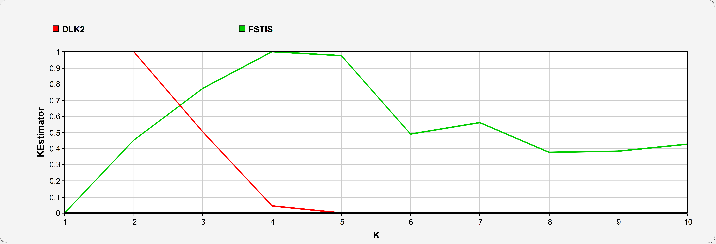

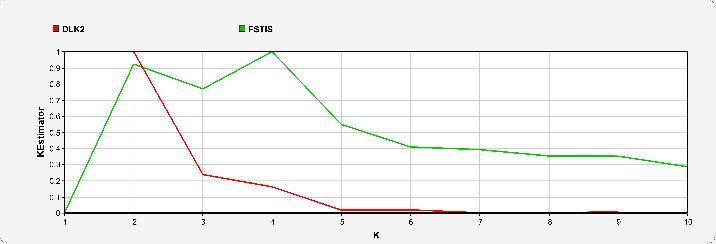


**a)**

**b)**

**c)**

**d)**

**Fig S17.** sNMF and PopCluster diagnostic plots for a) *C. amax, ­*b) *C. munda,* c) *Ct. quirinus* and d) *D. bilineata* samples from East Arnhem Land.

| **a)**  **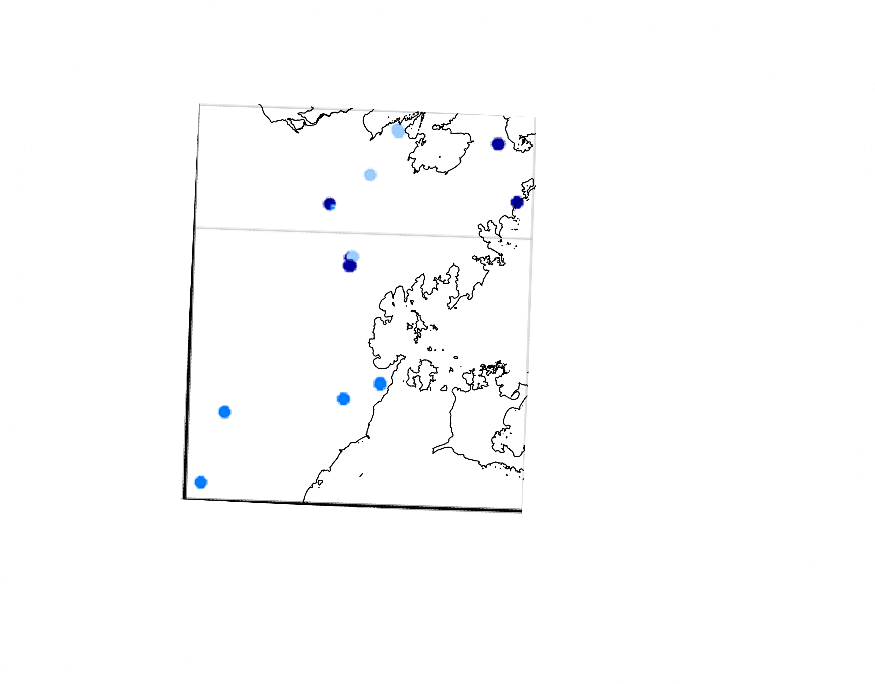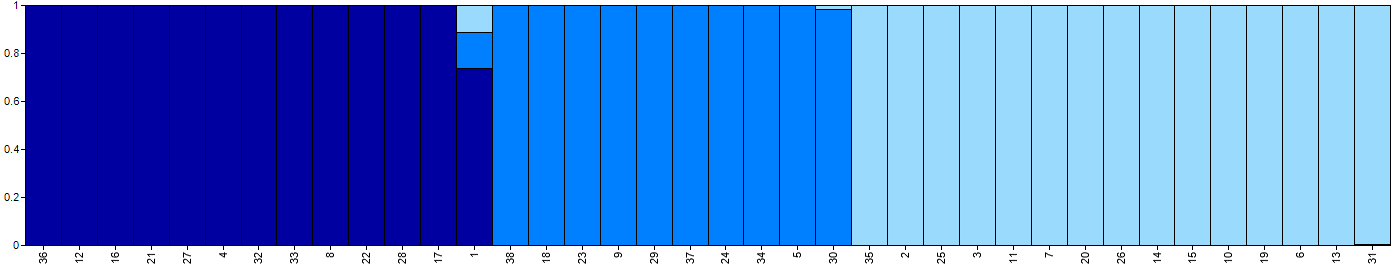** | **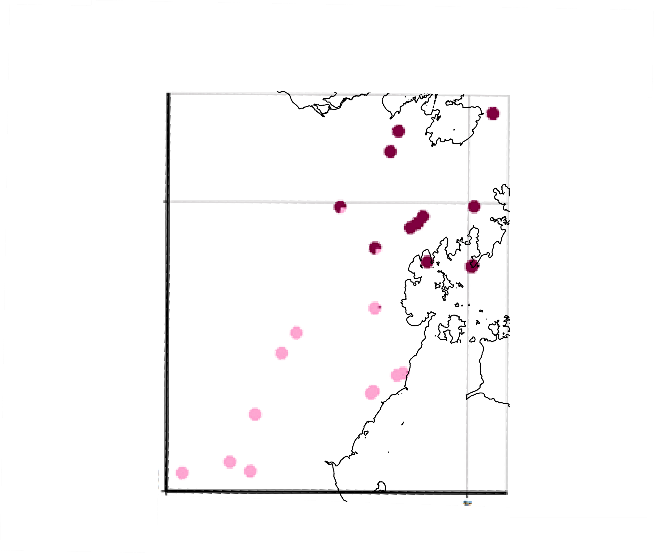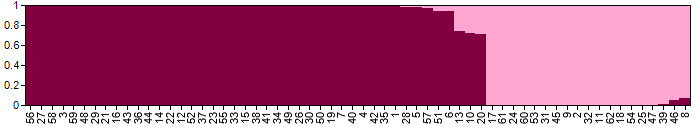b)** |
| --- | --- |
| **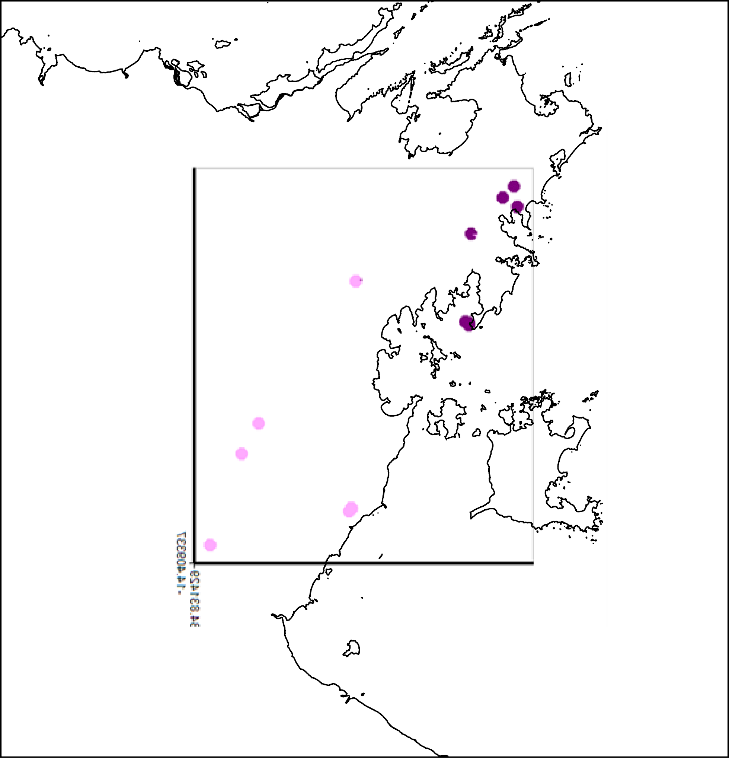c)**  **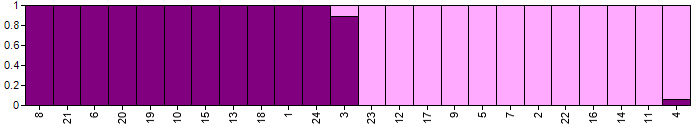** | **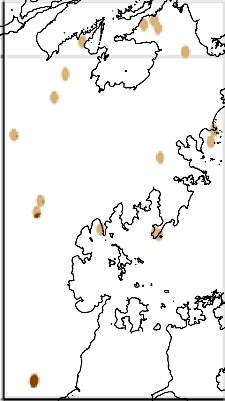d)**  **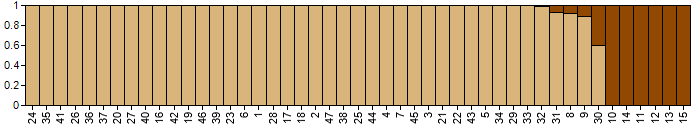** |
| **Fig S18.** PopCluster population structure analyses for a) *C. amax,* b) *C. munda,* c) *Ct. quirinus,* d) *D. bilineata.* | |

| **a)**  **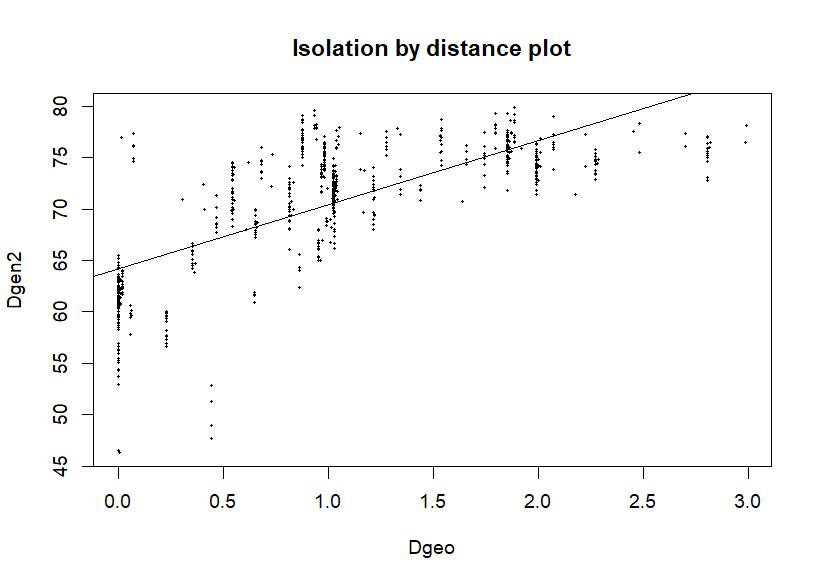** | **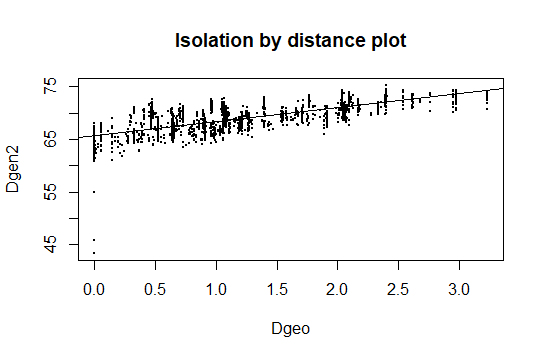b)** |
| --- | --- |
| **c)**  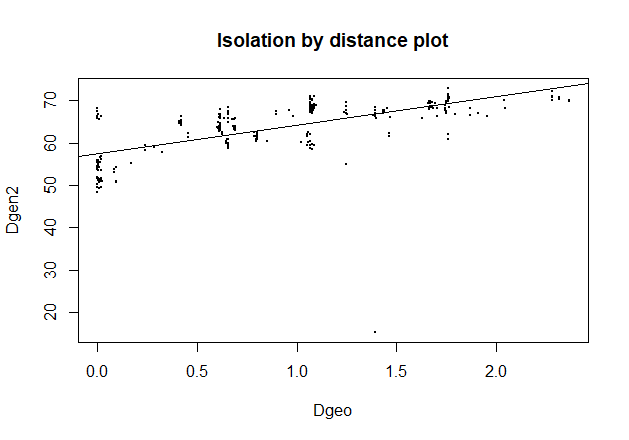 | **d)**  **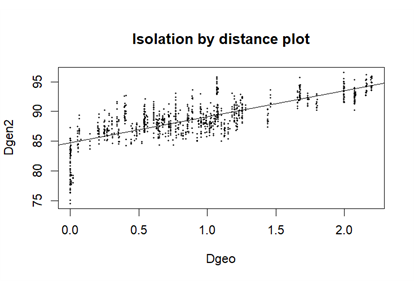** |
| **Fig S19.** Isolation by distance (IBD) plots for a) *C. amax,* b) *C. munda,* c) *Ct. quirinus* and d) *D. bilineata* samples from East Arnhem Land. | |
